# Benzoxazolinone-Based Propionyl Thiosemicarbazides as Multi-Target-Directed Ligands for Alzheimer’s Disease: Cholinesterase and MAO Inhibition, Docking, and Molecular Dynamics

**DOI:** 10.1021/acsomega.5c12925

**Published:** 2026-07-13

**Authors:** Hayrünnisa Taşci, Ahmet Avcı, Nadire Özenver, Begüm Nurpelin Sağlık Özkan, Birsen Tozkoparan, Nesrin Gökhan Kelekçi

**Affiliations:** † Department of Pharmaceutical Chemistry, Faculty of Pharmacy, EBYU, Erzincan 24100, Turkey; ‡ Department of Pharmaceutical Chemistry, Faculty of Pharmacy, 37515 Hacettepe University, Ankara 06100, Turkey; § Department of Pharmacognosy, Faculty of Pharmacy, Hacettepe University, Ankara 06100, Turkey; ∥ Department of Pharmaceutical Chemistry, Faculty of Pharmacy, 52944 Anadolu University, Eskişehir 26470, Turkey

## Abstract

Alzheimer’s disease (AD) benefits from multitarget-directed ligands (MTDLs) that can enhance cholinergic transmission while attenuating monoamine-oxidase-linked oxidative stress. Here, we report a benzoxazolinone-based propionyl thiosemicarbazide series, synthesized and fully characterized by infrared (IR) spectroscopy, ^1^H nuclear magnetic resonance (NMR), and high-resolution mass spectra (HRMS). The compounds showed consistent submicromolar inhibitory activity across AChE, BChE, MAO-A, and MAO-B *in vitro*. Several AChE potencies approached the reference donepezil, and selected BChE activities were within an order of magnitude of tacrine. Notably, 4bk′ (5-Me/benzyl) inhibited three targets (IC_50_: 0.029 ± 0.001 μM for AChE, IC_50_: 0.071 ± 0.003 μM for BChE, IC_50_: 0.048 ± 0.002 μM for MAO-B), and 4af′ (5-Cl/phenyl) showed a balanced profile (IC_50_: 0.025 ± 0.001 μM for AChE, IC_50_: 0.056 ± 0.002 μM for BChE, IC_50_: 0.095 ± 0.003 μM for MAO-A), 4ac′ (5-Cl/propyl) combined potent AChE and MAO-B inhibition (IC_50_: 0.035 ± 0.001 μM for AChE, IC_50_: 0.045 ± 0.002 μM for MAO-B), whereas 4ag′ (5-Cl/4′-Cl-phenyl) was strongly MAO-B-selective (IC_50_: 0.041 ± 0.001 μM for MAO-B). Antioxidant capacity was pronounced for para-substituted analogues. The efficient compounds 4bk′, 4af′, 4ac′, and 4ag′ presented quite low toxicity on healthy cells (cell survival % was above %70 for 4bk′, 4af′, and 4ac′, while it was around 64% for 4ag′) even when they were applied at 100 times higher concentrations than their IC_50_ values, which indicates that they are safe at effective doses. Moreover, assessment of the compounds at a concentration of 10 μM demonstrated no cytotoxic effects on either healthy BV-2 microglial cells or H9c2 rat myoblastoma cells. Docking and 100 ns molecular dynamics (MD) simulations (AChE: 4EY7; MAO-B: 2V5Z) supported stable binding for dual-active representatives (RMSD ∼1.5–2.8 Å). In silico ADME (QikProp) indicated compliance with Lipinski’s Rule of Five and Jorgensen’s Rule of Three. Collectively, this scaffold is tunable from MAO-B-selective to balanced MTDL profiles suitable for further AD-relevant optimization.

## Graphical abstract

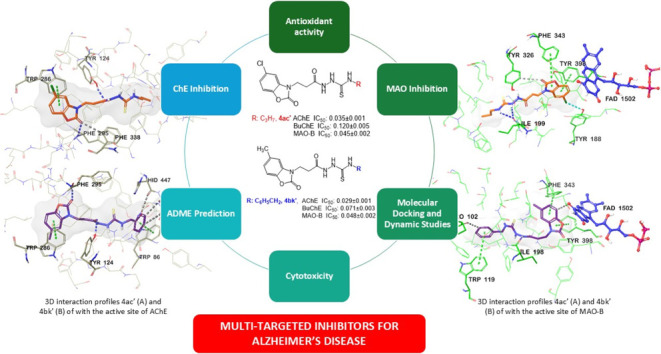

## Introduction

Alzheimer’s disease (AD) is a progressive, irreversible neurodegenerative disorder characterized by cognitive decline, memory impairment, and behavioral disturbances, primarily affecting the elderly population.^1^ The pathogenesis of AD is multifactorial, involving the accumulation of extracellular amyloid-beta (Aβ) plaques, intracellular neurofibrillary tangles composed of hyperphosphorylated tau protein, synaptic dysfunction, chronic neuroinflammation, and oxidative stress.^2^

Central to the cognitive deficits observed in AD is the disruption of the cholinergic neurotransmission system. Acetylcholine (ACh) is a crucial neurotransmitter involved in learning and memory processes. However, its synaptic availability is limited due to rapid hydrolysis by acetylcholinesterase (AChE) and butyrylcholinesterase (BChE) enzymes. Elevated activity of AChE and BChE contributes to reduced cholinergic signaling, exacerbating cognitive impairment. Consequently, AChE inhibitors represent the cornerstone of current symptomatic AD therapy, aimed at enhancing cholinergic transmission.^3–5^

In addition to cholinesterases, monoamine oxidases (MAOs), particularly the MAO-B isoform, play a significant role in AD pathology. MAO-B catalyzes the oxidative deamination of monoaminergic neurotransmitters such as dopamine, generating reactive oxygen species (ROS) as byproducts, thereby contributing to oxidative stress and neuronal damage. Increased MAO-B activity has been documented in AD brains and is implicated in promoting neurodegeneration. Therefore, MAO-B inhibitors are investigated as potential neuroprotective agents in AD and other neurodegenerative disorders.^6–8^

Oxidative stress impairs cellular function particularly in neurons when reactive species react with cellular constituents such as DNA, RNA, amino acids, carbohydrates, lipids, and proteins.^9^ Antioxidants mitigate this stress by acting as effective electron donors that neutralize excess free radicals and help restore redox balance.^10^ Consequently, antioxidants play a key role in the treatment of neurological diseases, including AD.^11^

Thiosemicarbazide and its derivatives have emerged as versatile scaffolds for modulating cholinesterases (AChE/BChE) and monoamine oxidases (MAO-A/MAO-B), two enzymatic families central to symptomatic management and disease-modifying strategies in neurodegeneration. The thiosemicarbazide moiety provides a polarized thioamide (−NH–C­(=S)–NH−) that can act as both hydrogen-bond donor and acceptor, while adjacent imine/aryl extensions (e.g., hydrazone formation) tune planarity, lipophilicity, and electronic density. In AChE, conjugated thiosemicarbazide hybrids can span the catalytic anionic site (CAS) and the peripheral anionic site (PAS), combining π–π stacking within the aromatic gorge with hydrogen bonding along the acyl pocket, thereby enabling dual-site engagement often associated with enhanced potency and anti-β-amyloid aggregation effects. In MAO, the same conjugation and heteroatom patterning favor productive placement within the FAD-containing cavity, supporting π–π contacts with the aromatic cage and H-bonding that can bias isoform selectivity (frequently toward MAO-B). Substituent effects on the aryl appendage (electron withdrawal substituents, halogens) further modulate binding energy and selectivity across both targets. Taken together, thiosemicarbazide-based chemotypes offer a chemically flexible platform for designing multitarget ligands that couple cholinesterase inhibition with MAO modulation in Alzheimer’s-relevant pharmacology.^12–20^ In line with their multimodal potential, several thiosemicarbazide-containing chemotypes display dual or multitarget activities relevant to neurodegeneration.^21–27^ Extending beyond enzyme inhibition profiles, thiosemicarbazone derivatives engineered as multitarget-directed ligands for Alzheimer’s disease such as pyridoxal 4-*N*-(1-benzylpiperidin-4-yl)­thiosemicarbazone (PBPT), compound 17, combine antiamyloid and antioxidant actions with autophagy induction and moderate AChE inhibition, underscoring the capacity of this scaffold to engage multiple disease-relevant pathways^28^ (Figure 1).

**Figure 1:**
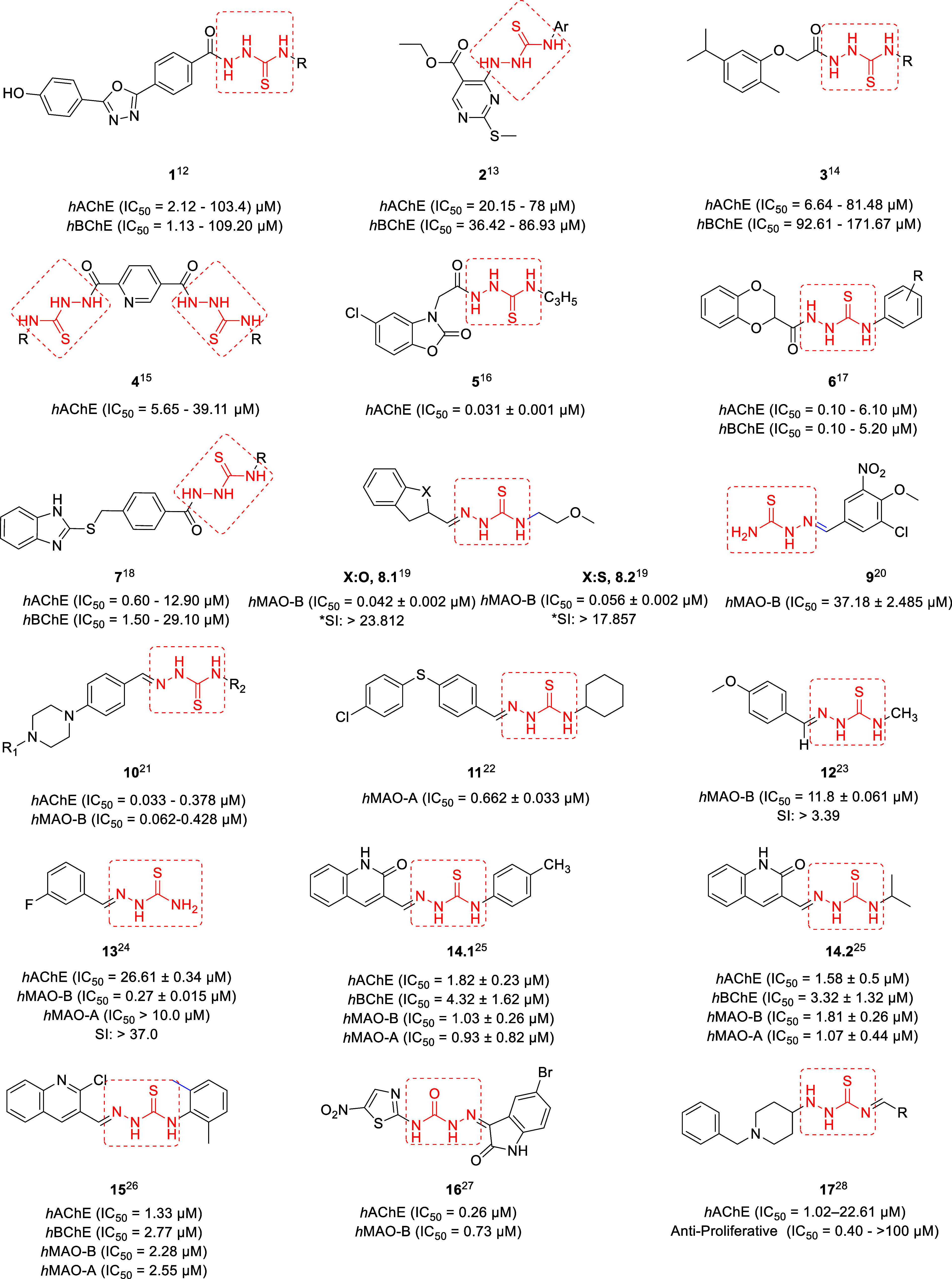
Some thiosemicarbazide derivative compounds designed as selective and/or multitarget cholinesterase and monoamine oxidase inhibitors (selectivity index-SI: *h*MAO-A/*h*MAO-B).

Classic anti-Alzheimer’s drugs such as donepezil, which contains an indan-1-one group, and riluzole, a benzothiazole derivative, exemplify how fused aromatic–heterocyclic scaffolds can serve as efficient pharmacophores for neurodegenerative modulation. Inspired by these structural motifs, medicinal chemistry studies have explored isosteric analogs in which benzo-fused frameworks are replaced by electronically comparable heterocycles such as benzimidazole,^29,30^ benzothiazole,^31–33^ and benzoxazole.^10^ Over the past several years, our research group and others have synthesized and biologically evaluated a variety of benzoxazolinone derivatives as antidepressant and cholinesterase inhibitors^16,34^ (Figure 2). In light of literature findings, the incorporation of a benzoxazolinone moiety into the thiosemicarbazide core was considered to provide a promising scaffold for the development of novel molecules possessing structural features capable of mimicking both acetylcholine and serotonin. Such dual resemblance may therefore confer enhanced potential for the simultaneous inhibition of cholinesterase and monoamine oxidase enzymes (Figure 2). In the development of such multitarget-directed ligands (MTDLs), the selection of targets must be carefully balanced to avoid undesirable off-target effects. While MAO-B inhibition is a key neuroprotective strategy, significant concurrent inhibition of MAO-A is generally avoided to mitigate the risk of tyramine-induced hypertensive crises, commonly known as the “cheese effect”. Furthermore, although BChE inhibition is recognized as a relevant target in advanced stages of Alzheimer’s Disease, the therapeutic priority remains focused on achieving potent and balanced AChE/MAO-B dual inhibition or targeted AChE/BChE/MAO-B triple inhibition. Based on these considerations, we hypothesized that hybrid molecules integrating the thiosemicarbazide backbone with a benzoxazolinone moiety may act as dual or even triple inhibitors of AChE, BChE, and MAO while simultaneously exerting antioxidant effects. Therefore, in the present study, we designed, synthesized, and biologically evaluated a new series of thiosemicarbazide–benzoxazolinone hybrids to explore their potential as multitarget-directed agents for the treatment of neurodegenerative diseases.

**Figure 2:**
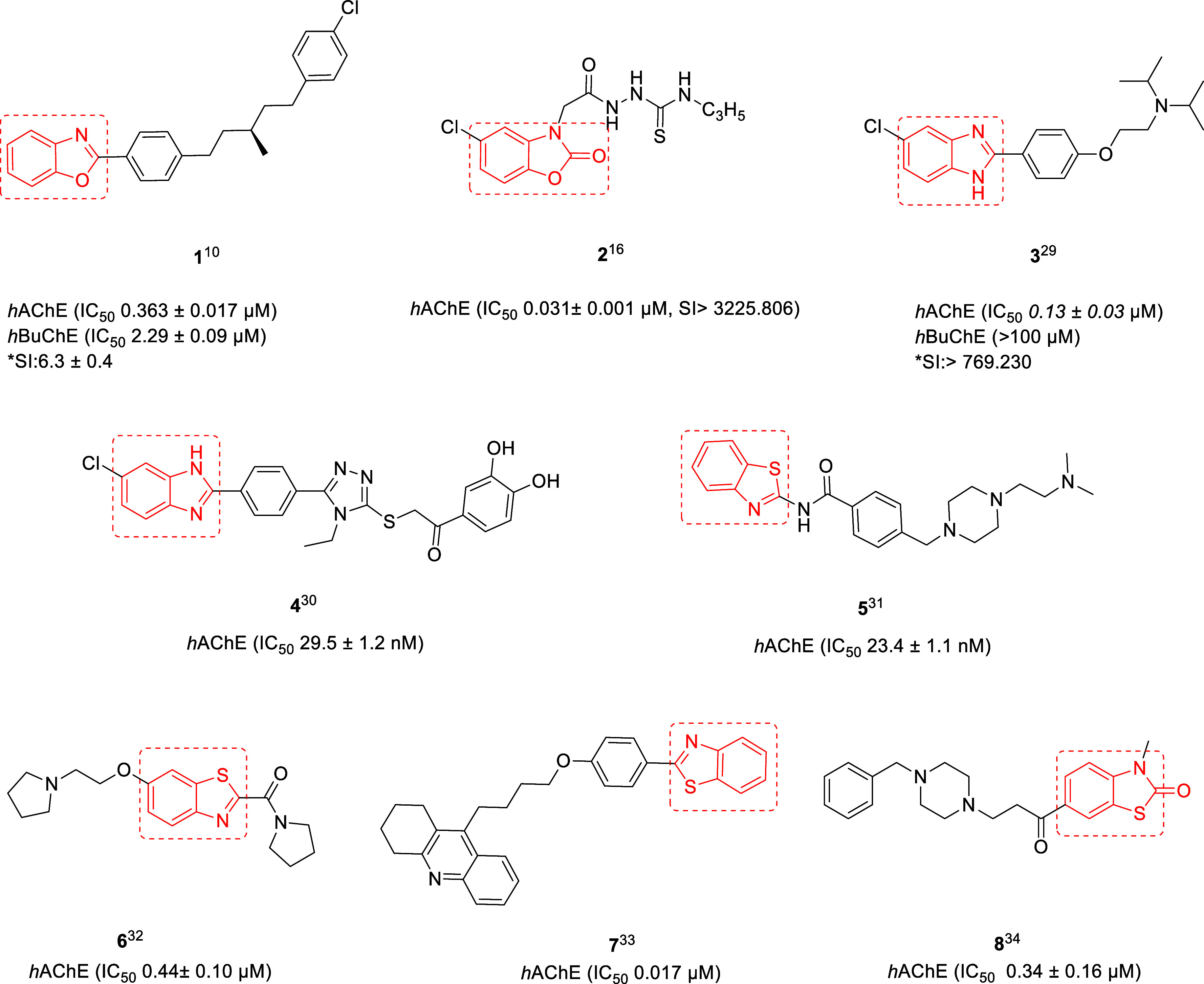
Some benzimidazole, benzothiazole, and benzoxazole derivatives are designed as cholinesterase inhibitors (selectivity index-SI: *h*BChE/*h*AChE).

## Results and Discussion

### Chemistry and Structural Elucidation

Scheme 1 illustrates the synthetic pathway used to obtain derivatives **4aa′**–**4bk’**. The target compounds were obtained in moderate yields in the final step, and the products were purified by recrystallization from suitable solvents. Structural assignments were corroborated by infrared spectroscopy, ^1^H-NMR, and ESI-MS. IR, ^1^H NMR, and ESI–MS data collectively confirm the structures of all benzoxazolinone–propionyl thiosemicarbazides bearing either a 5-methyl or 5-chloro substituent and diverse N-4 groups (alkyl, cyclohexyl, benzyl, or phenyl/*p*-substituted phenyl). Two diagnostic carbonyl absorptions are observed: the benzoxazolinone lactam C=O at 1760–1780 cm^–1^ and the carbazide C=O at 1680–1716 cm^–1^. A thioamide ν­(C=S) band at 1324–1381 cm^–1^, together with the absence of ν­(S–H), indicates that the solid-state tautomer is predominantly thione. Broad ν­(N–H) bands at 3200–3530 cm^–1^ arise from hydrogen-bonded amide and thioamide protons.

**Scheme 1:**
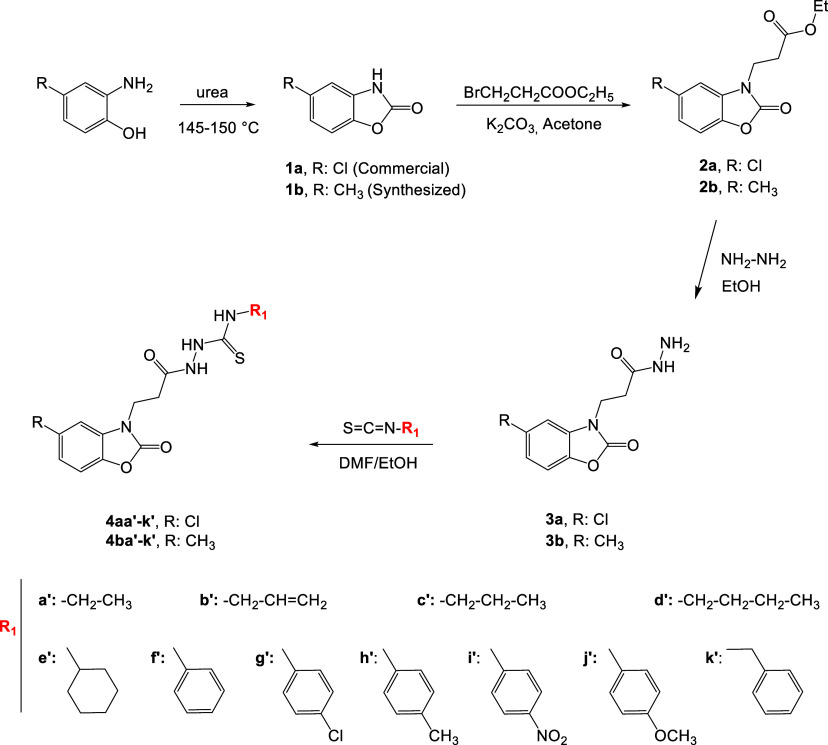
Synthesis of Thiosemicarbazide Derivatives

An A_2_X_2_-type ethylene spacer appears as two triplets at δ = 2.63–2.72 ppm (N–CH_2_) and δ = 4.02–4.07 ppm (CH_2_–CO). The benzoxazolinone 5-methyl group resonates as a singlet at δ ≈2.34 ppm. Three exchangeable NH singlets fall between δ ≈7.8 and 10.1 ppm, with chemical shifts modulated by hydrogen bonding and N-4 substitution (allyl, benzyl, cyclohexyl, OCH_3_, etc.). Chlorinated analogues display the characteristic [M + H]^+^/[M + H + 2]^+^ isotope cluster, while their nonchlorinated counterparts show only the monoisotopic [M + H]^+^ and [M + Na]^+^ ions. When all spectral data are evaluated as a whole, they confirm the structures of the compounds.

### Biological Evaluation

#### Cholinesterase and Monoamine Oxidase Inhibitory Activities

The inhibitory activities of the synthesized compounds against acetylcholinesterase (AChE), butyrylcholinesterase (BChE), monoamine oxidase A (MAO-A), and monoamine oxidase B (MAO-B) were determined using modified Ellman and fluorometric methods, respectively.^35^ Several members of the series showed submicromolar inhibition of AChE and/or MAO-B, with additional activity against BChE and, in some cases, MAO-A (Table 1). The acetylcholinesterase inhibitory effects of the compounds were found to be particularly remarkable.

**Table 1:**
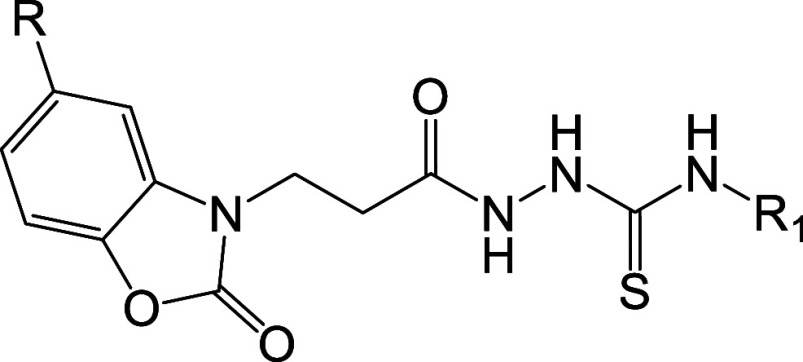

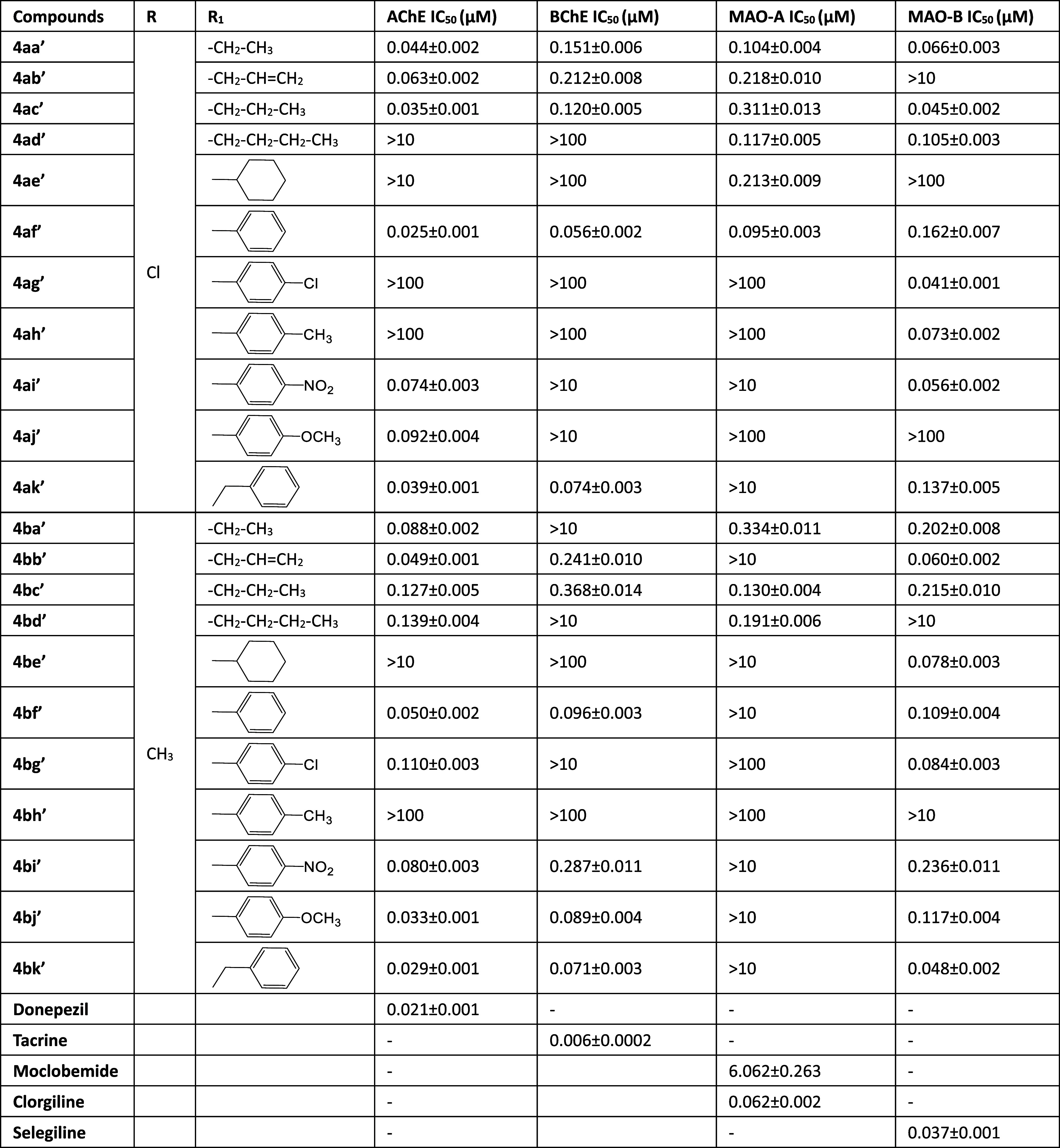
Inhibitory Activity (IC_50_, μM) of the Compounds against Recombinant Human AChE, BChE, MAO-A, and MAO-B Enzymes^a^

a The test results were expressed as the mean of quadruplicate assays.

Among the most broadly active analogues, the **4bk′** (CH_3_/benzyl) analogue showed a three-target profile (AChE 0.029 ± 0.001 μM; BChE 0.071 ± 0.003 μM; MAO-B 0.048 ± 0.002 μM), making it a high-priority candidate. The **4af**′ (Cl/phenyl) inhibited AChE, BChE, and MAO-A in the sub-0.10 μM range (AChE 0.025 ± 0.001 μM; BChE 0.056 ± 0.002 μM; MAO-A 0.095 ± 0.003 μM), with modest activity on MAO-B (0.162 ± 0.007 μM). This balanced, low-nanomolar profile marked **4bk′** and **4af′** as a credible multitarget-directed ligand (MTDL) leads within the series. Notably, compounds **4aa′** (Cl/ethyl; AChE: 0.044 ± 0.002, MAO-B: 0.066 ± 0.003), **4ac′** (Cl/propyl; AChE: 0.035 ± 0.001 μM, MAO-B: 0.045 ± 0.002 μM), **4ai′** (Cl/4-NO_2_phenyl; AChE: 0.074 ± 0.003, MAO-B: 0.056 ± 0.002), and **4bb′** (CH_3_/allyl; AChE: 0.049 ± 0.001, MAO-B: 0.060 ± 0.002) concurrently exhibited potent AChE and MAO-B inhibition. This dual inhibitory profile represents an attractive AChE/MAO-B phenotype with potential not only for symptomatic improvement but also for mitigating oxidative stress.

Compounds **4ak′** (Cl/benzyl), **4bf′** (CH_3_/phenyl), and **4bj′** (CH_3_/4-OCH_3_-phenyl) showed dual activity against both cholinesterases. For acetylcholinesterase (AChE), several analogues (for example, **4af′** (Cl/phenyl; 0.025 ± 0.001 μM) and **4bk′** (CH_3_/benzyl) 0.029 ± 0.001 μM) approach the potency of donepezil (IC_50_ = 0.021 ± 0.001 μM). **4ab′** (Cl/allyl; 0.063 ± 0.002 μM), **4aj′** (Cl/4-OCH_3_-phenyl; 0.092 ± 0.004 μM), **4ba′** (CH_3_/ethyl; 0.088 ± 0.002 μM), and **4bi′** (CH_3_/4-NO_2_-phenyl; 0.080 ± 0.003 μM) are within ∼3–4×. For butyrylcholinesterase (BChE), inhibition was weaker than that of tacrine (0.006 ± 0.0002 μM) but still remained notable for **4af′** (Cl/phenyl; 0.056 ± 0.002 μM), **4ak′** (Cl/benzyl; 0.074 ± 0.003 μM), **4bf′** (CH_3_/phenyl; 0.096 ± 0.003 μM), **4bj′** (CH_3_/4-OCH_3_-phenyl; 0.089 ± 0.004 μM), and **4bk′** (CH_3_/benzyl; 0.071 ± 0.003 μM).

Compound **4ag′** (Cl/4-Cl-phenyl) was found to be essentially MAO-B selective (MAO-B: 0.041 ± 0.001 μM; AChE/BChE/MAO-A >100 μM) and exhibited the closest activity to selegiline (0.037 ± 0.001 μM). Similarly, compounds **4ah′** (Cl/4-Me-phenyl), **4be′** (CH_3_/cyclohexyl), and **4bg′** (CH_3_/4-Cl-phenyl) also appeared to be selective toward MAO-B, although they were approximately 2-fold less potent than selegiline (0.037 ± 0.001 μM).

Conversely, **4ae′** (Cl/cyclohexyl) was comparatively MAO-A-skewed (0.213 ± 0.009 μM) while remaining inactive on the other targets at the highest tested concentrations.

A 5-chloro (Cl) substituent often enhanced potency, particularly for MAO-B, relative to the 5-methyl (CH_3_) counterpart. This is clear for matched pairs such as propyl (**4ac′** vs **4bc′**: MAO-B 0.045 vs 0.215 μM; AChE 0.035 vs 0.127 μM) and 4-Cl-phenyl (**4ag′** vs **4bg′**: MAO-B 0.041 vs 0.084 μM). An exception was the benzyl pair, where **4bk′** (CH_3_/benzyl) surpasses **4ak′** (Cl/benzyl) for AChE (0.029 vs 0.039 μM) and MAO-B (0.048 vs 0.137 μM), indicating that the electronic/steric interplay between the core and the N^4^-substituent can invert the trend. For phenyl analogues, **4af′** (Cl/phenyl) was more potent than **4bf′** (CH_3_/phenyl) on AChE and BChE (0.025 vs 0.050 μM; 0.056 vs 0.096 μM), again consistent with a beneficial effect of the chloro substituent in this subset.

Within the aliphatic series, chain elongation beyond propyl was detrimental: comparing Cl/ethyl → Cl/propyl → Cl/butyl showed an AChE optimum at propyl (0.044 → 0.035 → >10 μM) and a similar pattern for MAO-B (0.066 → 0.045 → 0.105 μM), suggesting that overfilling the pocket or increasing flexibility penalizes binding. The cyclohexyl group largely abolished activity on AChE, BChE, and MAO-B (Cl series), likely due to steric bulk. Interestingly, allyl displayed a core-dependent switch: Cl/allyl was inactive on MAO-B (>10 μM), but CH_3_/allyl recovered MAO-B potency (0.060 ± 0.002 μM), again pointing to context-dependent fit.

For Cl/phenyl derivatives, *para*-chloro (**4ag′**) induced MAO-B selectivity (AChE/BChE/MAO-A >100 μM; MAO-B 0.041 ± 0.001 μM), while para-methyl (**4ah′**) maintained MAO-B activity with weak cholinesterase/MAO-A inhibition. For CH_3_-core analogues, *para*-methoxy (**4bj′**) unexpectedly improved AChE potency relative to the chloro phenyl (0.033 vs 0.110 μM), with respectable BChE and MAO-B activity, indicating a favorable H-bonding/electronic contribution in this scaffold.

Taken together, these SAR trends support the notion that benzoxazolinone–thiosemicarbazide hybrids can be tuned across a spectrum from single-target selectivity (e.g., MAO-B-selective **4ag′**) to balanced MTDL profiles (e.g., **4bk′**, **4ac′**, **4aa′**, **4af′**). However, as a strategic priority for AD therapy, the most significant therapeutic advantage lies in achieving specific multitarget combinations rather than broad-spectrum inhibition. In this context, dual AChE/MAO-B inhibitors (such as **4ac′**) and triple AChE/BChE/MAO-B inhibitors (such as **4ak′** and **4bk′**) emerge as high-priority candidates for further preclinical screening, as they provide a balanced modulation of the cholinergic and oxidative pathways while minimizing potential off-target effects associated with MAO-A inhibition. This is precisely the design space envisioned for AD, where cholinergic enhancement and attenuation of MAO-mediated oxidative stress are both desirable.

### Antioxidant Activity

#### DPPH (1,1-Diphenyl-2-picrylhydrazyl) Radical Cation Scavenging Activity

The 1,1-diphenyl-2-picrylhydrazil (DPPH) radical scavenging activity assay is one of the most popular and frequently used methods depending on spectrophotometric measurements of the capacity of antioxidants to scavenge DPPH radicals.^36^ DPPH radical scavenging capacities of the compounds were tested at 12.5, 25, 50, 100, and 200 μg/mL concentrations. The scavenging percents of the compounds on the DPPH^+^ radical are displayed in Table 2. All the compounds exhibited concentration-dependent DPPH^+^ radical scavenging activity. IC_50_ values of the DPPH^+^ radical scavenging capacity are summarized in Table 2. The compounds **4ah′**, **4ba′**, **4bd′**, **4bf′**, **4bh′**, and **4bi**′ revealed better activity with lower IC_50_ values in all the tested compounds.

**Table 2:** Antioxidant Properties of the Compounds^a^

compounds	DPPH^+^ radical scavenging IC_50_ (μg/mL)	ABTS^+^ radical scavenging activity (mg TE/g compound)	CUPRAC (mg GAE/g compound)
**4aa′**	13.09 (38.19 μM)	42.56 ± 1.54	2.19 ± 0.33
**4ab′**	33.65 (94.84 μM)	41.04 ± 2.18	1.76 ± 0.35
**4ac′**	27.53 (77.15 μM)	41.93 ± 1.54	2.33 ± 0.05
**4ad′**	30.53 (82.32 μM)	38.22 ± 0.08	2.18 ± 0.16
**4ae′**	36.57 (92.14 μM)	37.29 ± 1.99	2.27 ± 0.16
**4af′**	26.65 (68.19 μM)	45.48 ± 0.51	2.27 ± 0.36
**4ag′**	18.83 (44.28 μM)	44.63 ± 0.15	2.18 ± 0.25
**4ah′**	12.15 (30.01 μM)	45.75 ± 0.20	1.97 ± 0.01
**4ai′**	26.33 (60.41 μM)	41.29 ± 1.58	2.55 ± 0.42
**4aj′**	15.38 (36.54 μM)	43.17 ± 2.03	2.70 ± 0.10
**4ak′**	70.59 (174.35 μM)	38.53 ± 0.23	1.87 ± 0.10
**4ba′**	12.07 (37.44 μM)	41.68 ± 0.13	2.17 ± 0.24
**4bb′**	14.14 (42.29 μM)	37.59 ± 0.63	1.69 ± 0.23
**4bc′**	15.92 (47.32 μM)	42.97 ± 0.04	2.06 ± 0.09
**4bd′**	12.12 (34.59 μM)	40.72 ± 0.34	2.62 ± 0.38
**4be′**	32.42 (86.12 μM)	39.89 ± 0.90	2.84 ± 0.11
**4bf′**	11.66 (31.48 μM)	46.27 ± 0.62	2.90 ± 0.06
**4bg′**	13.26 (32.75 μM)	44.03 ± 1.84	2.49 ± 0.16
**4bh′**	11.92 (31.01 μM)	46.35 ± 0.57	3.06 ± 0.00
**4bi′**	12.5 (30.09 μM)	44.88 ± 0.12	1.97 ± 0.49
**4bj′**	17.03 (42.53 μM)	42.19 ± 0.38	3.06 ± 0.09
**4bk′**	73.34 (190.77 μM)	42.03 ± 1.43	2.25 ± 0.17
**quercetin**	6.81 (22.53 μM)	-	-

a mg TE/g compound: mg Trolox equivalent/g compound; mg GAE/g compound: mg gallic acid equivalent/g compound.

#### ABTS (2,2′-Azino-bis­(3-ethylbenzothiazoline-6-sulfonic acid) Radical Cation Scavenging Activity

The ABTS radical cation scavenging activity assay depends on the neutralization of the ABTS radical cation in the existence of antioxidants and represents the total antioxidant activity of a substance.^37^ The ABTS radical cation scavenging activity of the compounds was calculated based on the relevant equation (*y* = 22.55*x* – 16.497) of the Trolox calibration curve. The experimental outcomes were expressed in terms of the Trolox equivalent antioxidant capacity (TEAC) in Table 2. A greater TEAC value corresponds to a higher antioxidant activity of the compounds. The compounds **4bh′** (46.35 ± 0.57 mg TE/g compound) and **4bf′** (46.27 ± 0.62 mg TE/g compound) displayed the highest ABTS^+^ radical scavenging activity among all the tested compounds, followed by the compounds **4ah′** (45.75 ± 0.20 mg TE/g compound), **4af′** (45.48 ± 0.51 mg TE/g compound), and **4bi′** (44.88 ± 0.12 mg TE/g compound). Like DPPH^+^ radical scavenging activity, the compounds **4ah′**, **4ba′**, **4bd′**, **4bf′**, **4bh′**, and **4bi′** exhibited higher and comparable ABTS^+^ radical scavenging activity. In comparison to DPPH^+^ radical scavenging activity, a greater antioxidant activity was obtained from **4af′**.

#### Cupric Ion Reducing Antioxidant Capacity (CUPRAC) Assay

Antioxidants do not only scavenge free radicals but also shorten higher valent elements such as copper and iron to their lower valence state. The redox potential of an antioxidant substance is of significance representing its efficacy. The redox potential-based method CUPRAC assay is based on the reduction of the copper­(II)–neocuproine complex to copper­(I)–neocuproine chelate by antioxidants.^38^ Cupric ion reducing antioxidant capacities of the compounds were ascertained according to the equation (*y* = 0.6824*x* – 0.7436) as gallic acid equivalent (mg gallic acid/g compound). The results are indicated in Table 2. The highest activity was demonstrated by the compounds **4bh′** and **4bj′** (each is 3.06 mg GAE/g compound), followed by **4bf′** (2.90 GAE/g compound), **4be′** (2.84 mg GAE/g compound), **4aj′** (2.70 mg GAE/g compound), **4bd′** (2.62 mg GAE/g compound), and **4ai′** (2.55 mg GAE/g compound). The experimental outcomes of the compounds are generally close to each other. The compounds **4ab′**, **4ak′**, **4ah′**. **4bb′**, and **4bi′** showed relatively lower cupric ion reducing antioxidant capacity in all.

### Cytotoxicity Evaluation

The most important risk factor for the new CNS drug candidates designed and developed, especially for neurodegenerative diseases, is the possibility of these compounds to exhibit neurotoxicity. Therefore, the potential cytotoxic effects of the all compounds were evaluated in both healthy BV-2 microglial cells and H9c2 cells at 10 μM in addition to the cytotoxicity evaluation under 100 μM treatment for 72 h. At the end of the procedure in which solutions of the compounds at a concentration of 100 μM were applied to H9c2 cells, it was determined that the cells showed viability rates ranging from 52.90 ± 4.20% to 92.61 ± 3.22%. When the safety profiles of the compounds were examined, the compounds **4be′** and **4bb′** were found to be favorable with cell viability percentages of 92.61 ± 3.22% and 88.18 ± 6.47%, respectively. The compound **4ah′** was found to have a less favorable safety profile with nearly 50% cell viability inhibition. Besides, the compounds did not exhibit any cytotoxicity toward BV-2 microglial cells and H9c2 cells.

Evaluating the compounds’ toxicity on both H9c2 and BV-2 cell lines displayed that none of the compounds were toxic at 10 μM. Furthermore, the compounds except for **4ah′**, **4bj′**, **4ag′**, and **4aj′** (ranked according to the decreasing toxicity) are even relatively safe at a quite high concentration 100 μM with the cell viability percentages above 70%. These data may provide an inference that these compounds may hold potential without inducing neurotoxicity (Figure 3A–3C). On the other hand, our previous findings demonstrated that doxorubicin reduced H9c2 cell viability by nearly half even at relatively low concentration (0.003 μM).^39,40^ Therefore, we evaluated all the compounds as considerably safe in comparison to clinically used drug doxorubicin.

**Figure 3:**
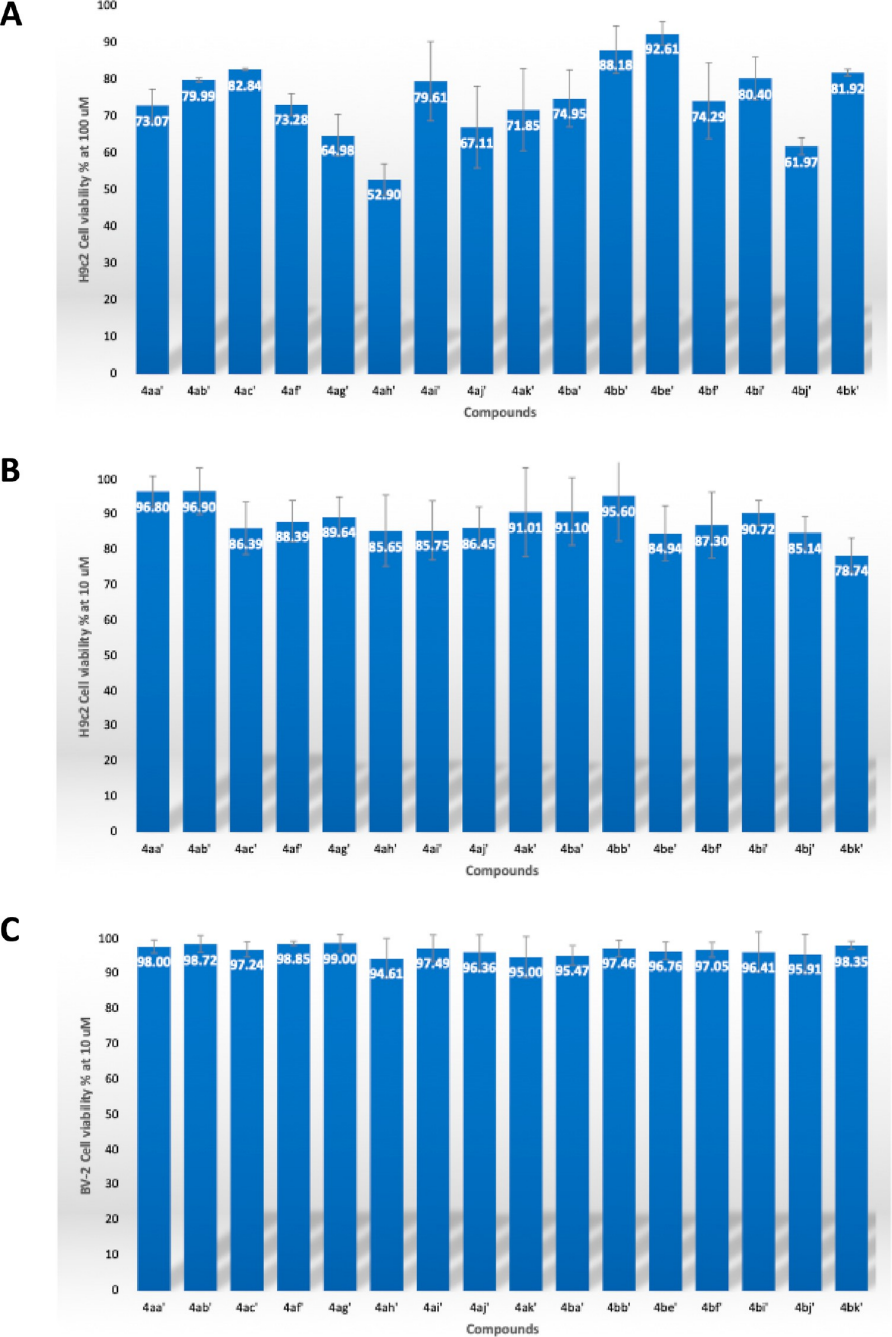
Cell viability % of the compounds (**4aa′**–**4bk′**) on the H9c2 cell line at 100 μM (A), on the H9c2 cell line at 10 μM (B), and on the BV-2 cell line at 10 μM (C).

### In Silico Studies

#### Prediction of ADME Parameters

The ADME properties of the synthesized compounds were assessed using the *QikProp* 4.8 software. In addition to evaluating ADME-related parameters, drug-likeness profiles were also analyzed through *QikProp*. To determine the drug-likeness of the compounds, Lipinski’s Rule of Five and Jorgensen’s Rule of Three were applied. The calculated parameters are presented in Table 3. As observed, all evaluated values fall within the acceptable reference ranges. Since none of the compounds showed multiple violations of either rule set, the obtained derivatives were considered compliant with established drug-likeness criteria.

**Table 3:** Calculated ADME Parameters of Compounds^a^

comp	MW	RB	DM	MV	DHB	AHB	PSA	log*P*	log*S*	PCaco	logBB	PMDCK	PM	% HOA	VRF	VRT
**4aa′**	342.799	5	6.773	1008.387	2.25	7.25	114.154	2.115	–4.200	428.101	–0.815	979.179	1	86.427	0	0
**4ab′**	354.810	6	4.169	1068.249	2.25	7.25	115.695	2.396	–4.746	293.828	–1.154	648.436	2	85.149	0	0
**4ac′**	356.826	6	6.582	1029.628	2.25	7.25	113.340	2.008	–3.854	339.568	–1.046	439.495	1	84.000	0	0
**4ad′**	370.853	7	5.928	1118.517	2.25	7.25	107.862	2.824	–4.979	520.050	–0.955	1237.998	1	92.092	0	0
**4ae′**	396.891	5	2.578	1177.519	2.25	7.25	104.206	3.332	–5.556	836.724	–0.591	1988.330	1	100.000	0	0
**4af′**	390.843	5	4.700	1129.021	2.25	7.25	116.899	2.846	–5.376	244.226	–1.226	500.665	2	86.345	0	0
**4ag′**	425.289	5	6.135	1172.389	2.25	7.25	116.891	3.319	–6.072	244.416	–1.086	1229.066	1	89.124	0	1
**4ah′**	404.870	5	4.254	1190.409	2.25	7.25	115.985	3.165	–5.855	269.414	–1.223	525.272	2	88.979	0	1
**4ai′**	435.841	6	3.655	1201.905	2.25	8.25	160.332	2.172	–5.474	32.764	–2.327	53.870	2	66.785	0	0
**4aj′**	420.870	6	3.715	1176.704	2.25	8	122.362	2.987	–5.042	512.605	–0.907	961.008	2	92.937	0	0
**4ak′**	404.870	6	6.258	1210.792	2.25	7.25	115.663	3.457	–6.021	312.984	–1.221	722.545	2	91.854	0	1
**4ba′**	322.381	5	9.357	1022.699	2.25	7.25	114.184	1.926	–3.995	436.006	–0.985	409.613	2	85.465	0	0
**4bb′**	334.392	6	6.219	1082.183	2.25	7.25	115.710	2.194	–4.469	293.656	–1.319	257.395	3	83.961	0	0
**4bc′**	336.408	6	7.082	1081.043	2.25	7.25	109.546	2.338	–4.408	534.048	–1.004	526.000	2	89.452	0	0
**4bd′**	350.435	7	6.385	1149.242	2.25	7.25	115.452	2.509	–4.823	287.329	–1.439	234.850	2	85.635	0	0
**4be′**	376.473	5	7.039	1224.669	2.25	7.25	114.805	3.036	–6.033	321.694	–1.334	311.299	2	89.603	0	1
**4bf′**	370.425	5	4.232	1144.053	2.25	7.25	115.315	2.808	–5.150	381.175	–1.178	335.041	3	89.586	0	0
**4bg′**	404.870	5	4.704	1187.420	2.25	7.25	115.306	3.282	–5.847	381.472	–1.034	822.481	2	92.367	0	1
**4bh′**	384.452	5	6.550	1204.343	2.25	7.25	116.000	2.964	–5.580	269.256	–1.389	208.505	3	87.795	0	0
**4bi′**	415.423	6	2.096	1215.840	2.25	8.25	160.347	1.970	–5.198	32.745	–2.485	21.384	3	65.599	0	0
**4bj′**	400.451	6	6.934	1149.015	2.25	8	115.757	2.556	–4.091	589.166	–0.915	433.679	3	91.494	0	0
**4bk′**	384.452	6	8.560	1225.334	2.25	7.25	115.678	3.263	–5.834	313.082	–1.405	293.987	3	90.718	0	1

a MW: molecular weight; RB: count of rotatable bonds (acceptable range: 0–15); DM: computed dipole moment (ideal range: 1–2.5); MV: total solvent-accessible volume (optimum range: 500–2000); DHB: predicted quantity of hydrogen-bond donors (acceptable range: 0–6); AHB: predicted quantity of hydrogen-bond acceptors (acceptable range: 2–20); PSA: van der Waals surface area contributed by polar nitrogen, oxygen, and carbonyl carbon atoms (optimum range: 7–200); log*P*: predicted octanol/water partition coefficient (ideal range: −2 to 6.5); log*S*: predicted aqueous solubility (optimum range: −6.5 to 0.5); PCaco: predicted apparent Caco-2 cell permeability coefficient (values <25 indicate poor, whereas >500 indicate excellent permeability); logBB: predicted brain/blood partition coefficient (acceptable range: −3 to 1.2); PMDCK: predicted apparent MDCK cell permeability coefficient (values <25 signify poor, whereas >500 signify excellent permeability); CNS: predicted central nervous system activity scored from −2 (completely inactive) to +2 (highly active) (expected range: −2 to +2); PM: number of potential metabolic transformations (ideal range: 1–8); %HOA: predicted percentage of human oral absorption (values > 80% denote high, while <25% denote poor absorption); VRF: number of infractions of Lipinski’s rule of five (criteria: MW <500, log*P* <5, DHB ≤5, AHB ≤10, along with a positive PSA value); VRT: number of infractions of Jorgensen’s rule of three (criteria: log*S* >−5.7, PCaco >22 nm/s, and PM <7).

A critical evaluation of the central nervous system (CNS) drug candidates necessitates a thorough analysis of their ability to cross the blood–brain barrier (BBB). In this study, the BBB permeability was predicted using logBB and PMDCK parameters (Table 3). The calculated logBB values, ranging from −0.591 to −2.485, indicate that the compounds are within the acceptable range for brain penetration. Notably, the PMDCK values, which represent a crucial indicator of passive diffusion across the BBB, were found to be highly promising. Specifically, several compounds (e.g., **4ad′**, **4ae′**, and **4ag′**) exhibited PMDCK values exceeding 500 nm/s (up to 1988.330 nm/s), which is classified as excellent permeability by the QikProp criteria. It is thought that the Cl substitution at the fifth position of the benzoxazolinone ring contributes to membrane permeation by increasing the lipophilicity of the compounds. The lead dual inhibitors **4ac′** and **4bk′** also showed favorable PMDCK scores of 439.495 and 293.987 nm/s, respectively. These in silico findings suggest that the synthesized benzoxazolinone–thiosemicarbazide hybrids possess a suitable pharmacokinetic profile to penetrate the CNS and engage with the target enzymes in the brain.

#### Molecular Docking Studies

To further investigate the molecular basis of the high inhibitory potencies observed in the *in vitro* assays, structure-based *in silico* studies were conducted. Since the primary objective of our MTDL design was to target AChE and MAO-B where the most significant submicromolar activities were achieved, molecular docking and dynamics simulations were specifically focused on these two enzymes to elucidate the key binding interactions of the lead candidates. In this context, computational docking experiments were first performed on the AChE enzyme. Based on the data gathered from the *in vitro* AChE screening, a diverse spectrum of inhibitory potencies was noted among the synthesized series. Consequently, a subset comprising the five most effective derivatives (**4ac′**, **4af′**, **4ak′**, **4bj′**, and **4bk′**) was earmarked for thorough molecular docking investigations. The obtained low-energy binding orientations are visualized in Figure 4. Additionally, a comprehensive breakdown of the key binding interactions established by these top candidates within the catalytic gorge of the enzyme (PDB ID: 4EY7) is summarized in Table 4.

**Figure 4:**
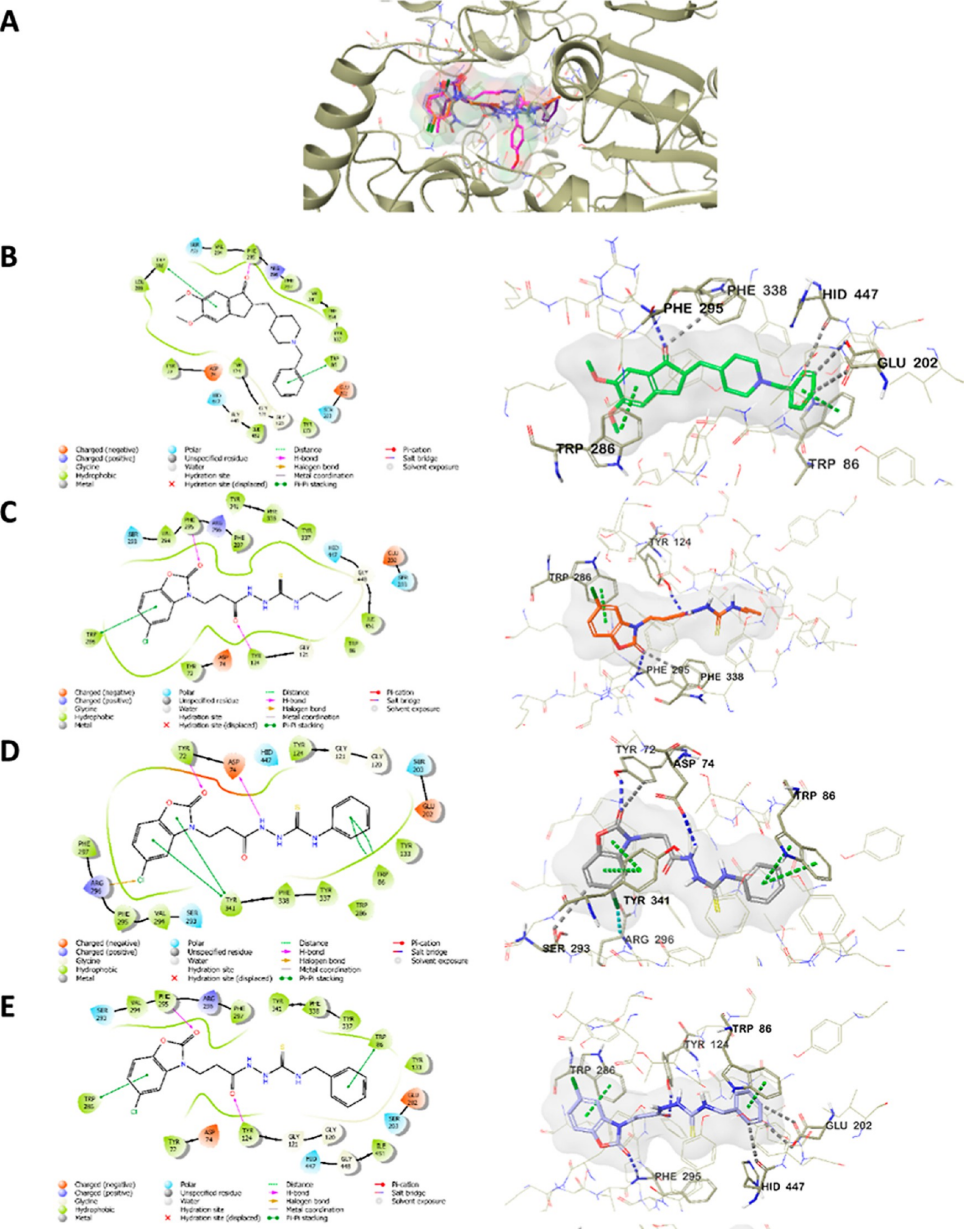

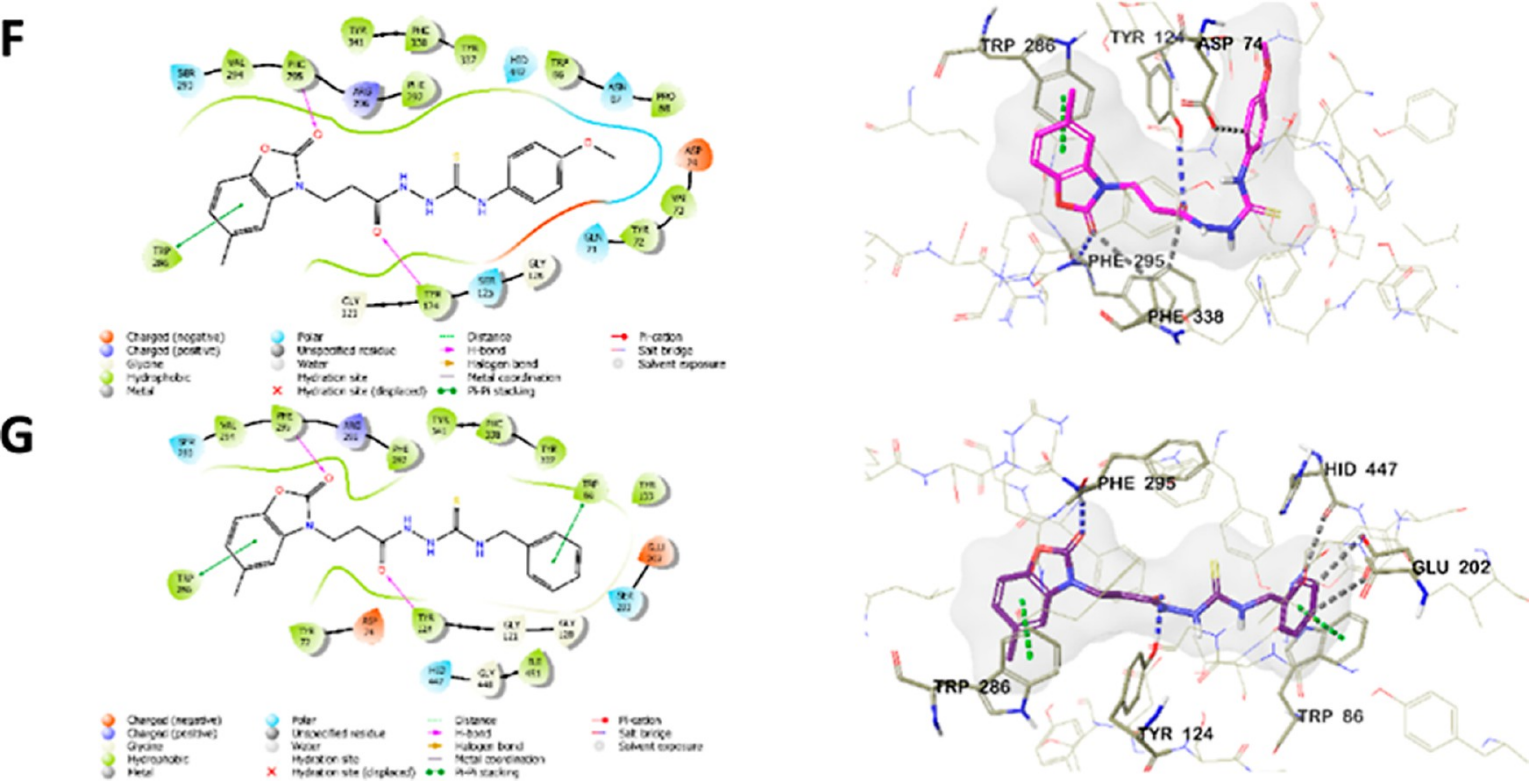
(A) Conformational superposition displaying the binding orientations of derivatives **4ac′**, **4af′**, **4ak′**, **4bj′**, and **4bk′** within the AChE pocket. 2D and 3D molecular contact networks representing reference drug donepezil (B) and the synthesized hybrids **4ac′** (C), **4af′** (D), **4ak′** (E), **4bj′** (F), and **4bk′** (G) inside the AChE binding gorge (PDB ID: 4EY7).

**Table 4:** Detailed Breakdown of Non-Covalent Docking Interactions Established by the Top Candidates (**4ac′**, **4af′**, **4ak′**, **4bj′**, and **4bk′**) inside the Human AChE Active Pocket

compound	moiety	Pi–pi interactions	hydrogen bonds	aromatic hydrogen bonds	halogen bonds
**4ac′**	benzoxazole	indole ring of Trp286	-	-	-
	carbonyl (on benzoxazole)	-	amide NH of Phe295	phenyl of Phe338	-
	carbonyl	-	hydroxyl of Tyr124	-	-
**4af′**	5-Cl	-	-	-	guanidine moiety of Arg296 (charge-reinforced interaction)
	benzoxazole	phenyl ring of Tyr341	-	carbonyl of Ser293	-
	carbonyl (on benzoxazole)	-	hydroxyl of Tyr72	phenyl of Tyr72	-
	secondary amine	-	carbonyl of Asp74	-	-
	phenyl	indole ring of Trp86	-	-	-
**4ak′**	benzoxazole	indole ring of Trp286	-	-	-
	carbonyl (on benzoxazole)	-	amide NH of Phe295	-	-
	carbonyl	-	hydroxyl of Tyr124	-	-
	phenyl	indole ring of Trp86	-	carbonyl of Glu202	-
				carbonyl of His447	
**4bj′**	benzoxazole	indole ring of Trp286	-	-	-
	carbonyl (on benzoxazole)	-	amide NH of Phe295	phenyl of Phe338	-
	carbonyl	-	hydroxyl of Tyr124	phenyl of Phe338	-
	phenyl	-	-	hydroxyl of Asp74	-
**4bk′**	benzoxazole	indole ring of Trp286	-	-	-
	carbonyl (on benzoxazole)	-	amide NH of Phe295	-	-
	carbonyl	-	hydroxyl of Tyr124	-	-
	phenyl	indole ring of Trp86	-	carbonyl of Glu202	-
				carbonyl of His447	

As illustrated in Figure 4A, a high degree of conformational alignment is evident between the reference drug donepezil and the evaluated derivatives (**4ac′**, **4af′**, **4ak′**, **4bj′**, and **4bk′**), all of which occupy the acetylcholinesterase active cavity through closely matched orientations and interaction types. Literature widely recognizes that the topographical architecture of the AChE binding gorge is fundamentally divided into two strategic domains: the catalytic anionic site (CAS) located deep within the pocket and the peripheral anionic site (PAS) situated at the entrance. In donepezil, the indanone ring interacts with Trp286 and Phe295, allowing it to bind at the PAS, while the benzylpiperidine moiety primarily interacts with Trp86, enabling binding at the CAS region.^41^ The compounds evaluated in this study exhibited similar interactions, suggesting a dual-binding mode to the AChE enzyme. Specifically, the benzoxazol-2-one core in these compounds formed π–π interactions with Trp286 and Phe295, anchoring the molecule at the PAS. Meanwhile, the terminal aromatic groups; phenyl (**4af′**), 4-methoxyphenyl (**4bj′**), and benzyl moieties (**4ak′** and **4bk′**), engaged in π–π interactions with Trp86, facilitating their orientation toward the CAS region. Additionally, the thiourea and amide moieties, which serve as linkers between the two aforementioned core structures, were also found to interact with Tyr124. In addition to the common interactions described above, compound **4af′** uniquely exhibited a specific interaction between the chlorine atom at the 5-position of its benzoxazolinone ring and the side chain of Arg296. Detailed analysis suggests that this interaction occurs with the protonated guanidine moiety of Arg296, characterizing it as a charge-reinforced interaction. This specific contact may account for the slightly superior inhibitory potency of **4af′** compared to the other potent analogues in the series (IC_50_ = 0.025 ± 0.001 μM).

In summary, the computational docking insights demonstrated that the targeted derivatives (**4ac′**, **4af′**, **4ak′**, **4bj′**, and **4bk′**) accommodate into the acetylcholinesterase active pocket by mirroring the conformational traits and contact networks of the reference inhibitor donepezil. By simultaneously engaging with the catalytic anionic site (CAS) and the peripheral anionic site (PAS), the series successfully displayed a classic dual-site binding behavior. Taken together, these data strongly indicate that the engineered scaffold holds a pronounced inhibitory capacity against AChE, justifying its ongoing optimization and advancement as a valuable class of anticholinesterase candidates.

In the subsequent stage of the computational docking workflow, the binding characteristics within the human MAO-B active pocket were comprehensively investigated. To achieve this, the four derivatives demonstrating the most potent *in vitro* MAO-B inhibition (**4ac′**, **4ag′**, **4ai′**, and **4bk′**) were singled out for molecular docking runs. The predicted low-energy binding conformations of these prominent candidates are illustrated in Figure 5. Furthermore, a detailed evaluation of the specific noncovalent networks established by these hybrids inside the MAO-B enzymatic cavity (PDB ID: 2V5Z) is systematically cataloged in Table 5.

**Figure 5:**
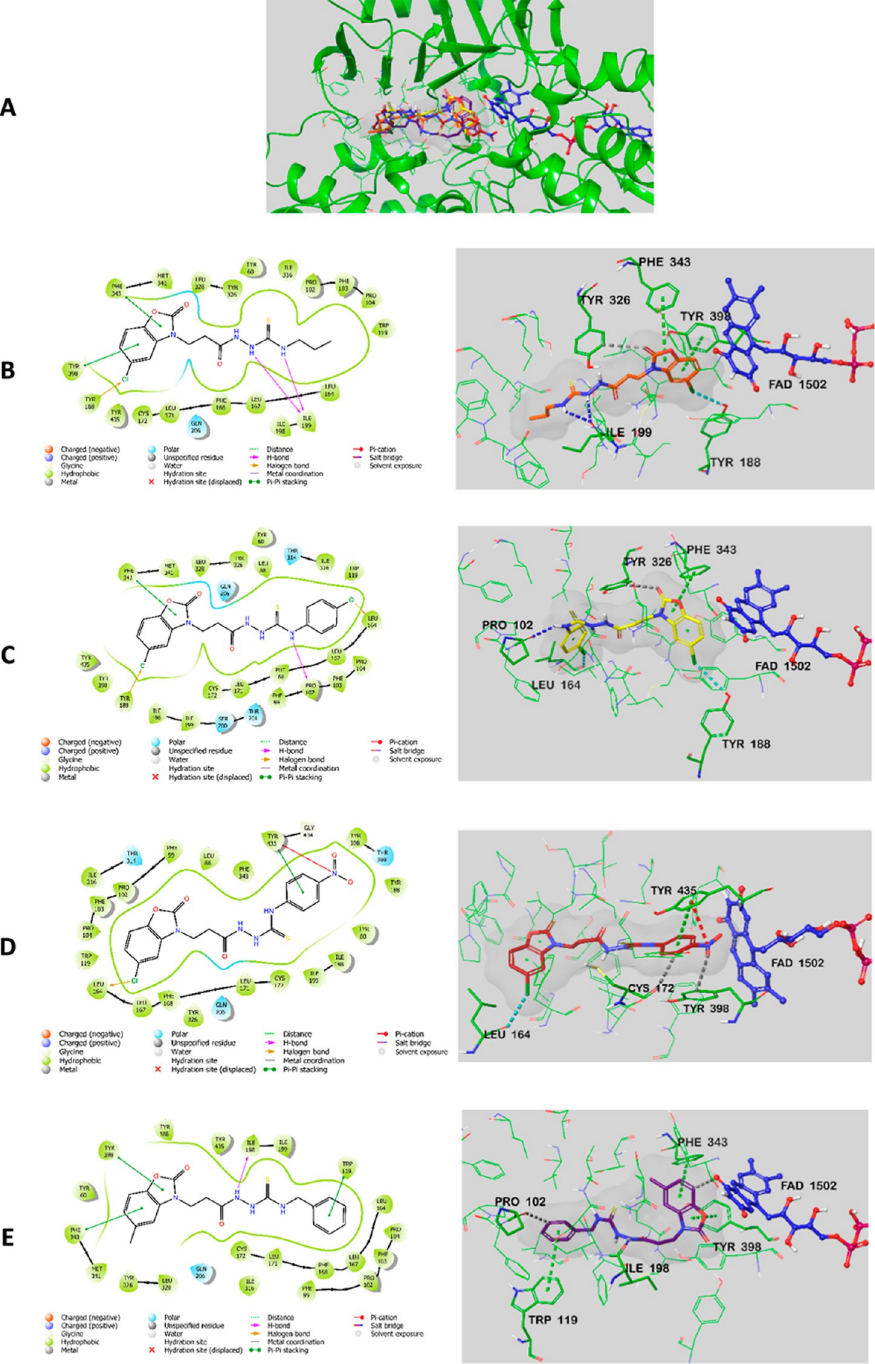
(A) Conformational superposition displaying the binding orientations of derivatives **4ac′**, **4ag′**, **4ai′**, and **4bk′** within the human MAO-B pocket. 2D and 3D molecular contact networks representing the synthesized hybrids **4ac′** (B), **4ag′** (C), **4ai′** (D), and **4bk′** (E) inside the active cleft of MAO-B (PDB ID: 2V5Z).

**Table 5:** Detailed Breakdown of Non-Covalent Docking Interactions Established by the Prominent Candidates (**4ac′**, **4ag′**, **4ai′**, and **4bk′**) inside the Human MAO-B Active Pocket

compound	moiety	Pi–pi interactions	cation-pi interactions	hydrogen bonds	aromatic hydrogen bonds	halogen bonds
**4ac′**	5-Cl	-	-	-	-	hydroxyl of Tyr188
	benzoxazole	phenyl of Phe343	-	-	-	-
		phenyl of Tyr398				
	secondary amine	-	-	carbonyl of Ile199	-	-
	carbonyl (on benzoxazole)	-	-	-	phenyl of Tyr326	-
**4ag′**	4′-Cl	-	-	-	-	carbonyl of Leu164
	5-Cl	-	-	-	-	hydroxyl of Tyr188
	benzoxazole	phenyl of Phe343	-	-	-	-
	secondary amine	-	-	carbonyl of Pro102	-	-
	carbonyl (on benzoxazole)	-	-	-	phenyl of Tyr326	-
**4ai′**	5-Cl	-	-	-	-	carbonyl of Leu164
	phenyl	phenyl of Tyr435	-	-	carbonyl of Cys172	-
	4′-nitro	-	phenyl of Tyr435	-	phenyl of Tyr398	-
**4bk′**	benzoxazole	phenyl of Phe343	-	-	FAD molecule	-
		phenyl of Tyr398				
	secondary amine	-	-	carbonyl of Ile198	-	-
	phenyl	phenyl of Trp119	-	-	carbonyl of Pro102	-

As shown in Figure 5A, the selected compounds (**4ac′**, **4ag′**, **4ai′**, and **4bk′**) occupy the same hydrophobic pocket within the MAO-B active site. However, a detailed analysis revealed that **4ai′** adopts a reversed orientation relative to the other analogues. In **4ai′**, the *para*-nitro substituted phenyl moiety is oriented toward the FAD cofactor, facilitating a unique cation–π interaction with Tyr435 and an aromatic hydrogen bond with Tyr398 (Table 5). This orientation is likely driven by the strong electron-withdrawing nature of the nitro substituent, which redefines the electrostatic complementarity within the bipartite cavity of MAO-B. In contrast, for compounds like **4ac′** and **4ag′**, the benzoxazolinone core is positioned deeper into the catalytic site. This observation underscores that even minor structural modifications on the N-4-aryl appendage can significantly modulate the binding trajectory within the MAO-B isoform.

Typically, the benzoxazole framework engaged in pi–pi stacking contacts with the aromatic side chains of Phe343 and Tyr398. Furthermore, the thiourea spacer was highlighted as a pivotal motif, facilitating polar interaction networks. More precisely, the secondary amine features within this central linker establish hydrogen bonds with the key amino acids Ile198 and Ile199, both of which are recognized for their integral participation in the substrate-binding domain. Engaging these particular amino acid residues is deemed to be essential for anchoring ligands effectively inside the MAO-B cavity. Consequently, such distinctive binding configurations offer a robust structural rationale for the prominent *in vitro* MAO-B inhibitory potency displayed by this series.

According to the *in vitro* enzymatic screenings against MAO-B, the most potent inhibitory responses within the synthesized series were elicited by compounds **4ac′** and **4ag′**, which yielded outstanding IC_50_ values of 0.045 ± 0.002 μM and 0.041 ± 0.001 μM, respectively. It is highly probable that the structural integration of chlorine substituents acts as the driving force behind this pronounced enhancement in biological efficacy. Molecular docking results support this hypothesis, as halogen bonds were observed between the chlorine substituents and key residues Leu164 and Tyr188 in both compounds. Halogen atoms have been shown to act not only as passive groups that increase lipophilicity in MAO-B inhibitor design but also as active pharmacophores that direct binding interactions. They are known to enhance binding affinity in the hydrophobic active site of MAO-B and strengthen π–π van der Waals interactions by forming “halogen bonding” (σ-hole interactions). Furthermore, studies have shown that the type of halogen, and especially its position in the aromatic ring (mostly para and meta), is decisive in inhibitor potency and MAO-B selectivity, and that in appropriate placement they support isoform selectivity by increasing compatibility with the larger hydrophobic pocket of MAO-B.^42^

In conclusion, the prioritized candidates exhibited optimal spatial configurations within the human MAO-B active cleft, establishing vital pi–pi stacking and hydrogen-bonding networks with the critical amino acid residues Phe343, Tyr398, Ile198, and Ile199. These specific noncovalent contacts robustly substantiate the pronounced *in vitro* enzymatic blockade achieved by the series, underlining their potential as highly viable MAO-B inhibitory leads for future preclinical optimization.

To further corroborate these *in vitro* enzymatic trends from a structural perspective and unravel the molecular basis of isoform selectivity, cross-docking simulations were subsequently performed against human BChE and MAO-A enzymes. For this purpose, three key representative compounds were strategically selected: **4ac′** and **4bk′**, which demonstrated prominent dual AChE/MAO-B inhibitory profiles and were extensively evaluated in subsequent molecular dynamics simulations, and **4af′**, which emerged as the most effective derivative against BChE and MAO-A in the *in vitro* assays. The docking poses of these ligands on the related enzymes are given in Figure 6.

**Figure 6:**
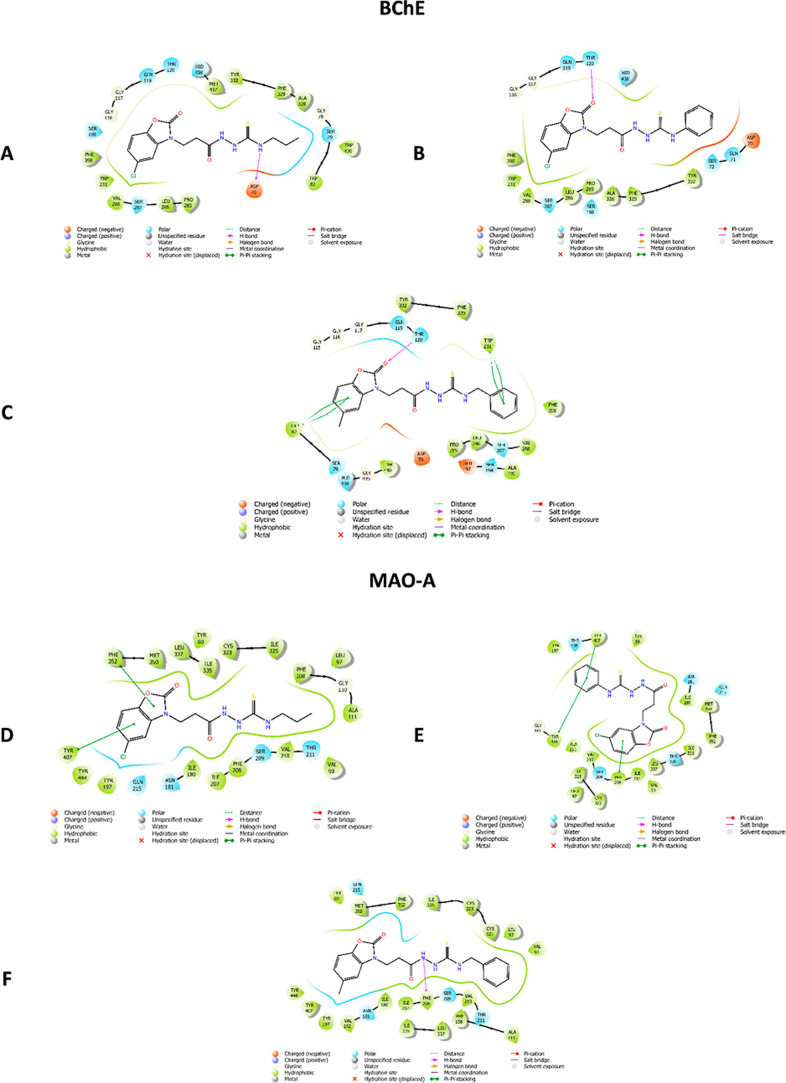
2D molecular contact networks representing the synthesized hybrids **4ac′** (A), **4af′** (B), and **4bk′** (C) inside the human BChE binding cavity (PDB ID: 4BDS). 2D specific noncovalent interactions of derivatives **4ac′**(D), **4af′** (E), and **4bk′** (F) within the active cleft of human MAO-A (PDB ID: 2Z5X).

When these representative compounds were docked into the active site of BChE, they were distinctly lacking in precise geometric fit and critical binding contacts compared to their AChE counterparts. Structural analysis of the binding poses revealed that compound **4ac′** formed only a single hydrogen bond with Asp70, while compound **4af′** established only one hydrogen bond with Thr120. In the case of **4bk′**, the molecule participated in pi–pi stacking interactions with Trp82 and Trp231, alongside a single interaction with Thr120. These sparse interaction profiles clearly indicate that the evaluated compounds possess a significantly lower binding affinity and poor complementary fit toward BChE compared to the highly stabilized network characteristic of their AChE complexes.

Similarly, the cross-docking analysis within the MAO-A active cavity provided a clear structural rationale for the high MAO-B selectivity observed across the series. In the MAO-A binding pocket, compounds **4ac′** and **4af′** established pi–pi stacking interactions between their benzoxazolinone core and the aromatic side chains of Phe208, Phe352, and Tyr407. For compound **4af′**, supplementary pi–pi contacts were also detected between its terminal phenyl ring and the residues Tyr407 and Tyr444. Conversely, compound **4bk′** was found to engage in only a single hydrogen bond with Phe208. Based on these interaction profiles, it is evident that the representative derivatives suffer from severe spatial mismatches or steric restrictions within the more constrained MAO-A binding cavity, thereby preventing a productive alignment near the FAD cofactor.

Overall, these comparative *in silico* evaluations clearly revealed that the investigated compounds could not establish relevant interactions or highly favorable binding scores with either BChE or MAO-A, standing in stark contrast to their robust, high-affinity binding profiles observed with AChE and MAO-B. This computational cross-validation successfully demonstrates that the established protocol accurately aligns with the experimental boundaries of target affinity, confirming that the structural architectures of these derivatives are optimized for selective dual-target intervention against AChE and MAO-B.

The divergent inhibitory profiles of the matched molecular pairs (5-Cl vs 5-Me) underscore the importance of the benzoxazolinone 5-position in modulating isoform selectivity and potency. The high MAO-B over MAO-A selectivity (e.g., **4ag′**, SI >2439) is likely driven by the favorable accommodation of the para-substituents within the MAO-B bipartite cavity, where the gatekeeper Ile199 offers a more accommodating environment than the bulkier Phe208 in MAO-A. Conversely, for bulky analogues like the cyclohexyl derivative **4ae′**, the 5-Cl substituent may trigger steric hindrance, leading to a loss of MAO-B affinity—a trend reversed in the smaller 5-Me analogue **4be′**.

Furthermore, the superior AChE potency of the 5-Cl series can be rationalized by the formation of halogen-mediated interactions. As highlighted in the literature regarding σ-holes,^43,44^ the 5-Cl atom can engage in noncovalent interactions with nearby nucleophilic centers, such as the backbone carbonyls or the side chain of **Arg296** (Cl–H–N^+^), providing a “charge-reinforced” stabilization that the 5-Me group cannot replicate. These structural nuances explain why 5-Cl-substituted hybrids often represent the most potent lead candidates in this scaffold.

In conclusion, the structure-based simulations firmly demonstrated that the prioritized derivatives establish robust and targeted noncovalent interactions within the active pockets of both AChE and MAO-B enzymes. Particularly, hybrids **4ac′** and **4bk′** exhibited a distinctive dual-target inhibition profile, effectively modulating both enzymatic pathways simultaneously. This balanced multitarget mechanism underscores their potential viability as promising therapeutic leads in the management of Alzheimer’s disease, where concurrent blockade of AChE and MAO-B is expected to deliver synergistic clinical outcomes.

#### Molecular Dynamic Studies on the AChE and MAO-B Enzyme

To explore the time-dependent structural behavior of the ligand–enzyme complexes, 100 ns computational molecular dynamics (MD) trajectories were generated based on the human AChE (PDB ID: 4EY7) and MAO-B (PDB ID: 2V5Z) crystal coordinates incorporated into a POPE lipid bilayer architecture. Individual simulation setups were constructed for the highly effective derivatives **4ac′** and **4bk′**, both of which stood out as the most prominent dual-acting inhibitors against both target enzymes in the *in vitro* assays. Compounds **4ac′** and **4bk′** were prioritized for in-depth molecular dynamics simulations (MDS) due to their highly balanced and potent dual inhibitory profiles against both AChE and MAO-B. Despite the moderate activity of **4ac′** against MAO-A, the exceptional efficacy of these compounds on the primary targets of the MTDL strategy made them crucial candidates for elucidating the structural stability and binding characteristics required for simultaneous cholinesterase and MAO-B modulation. The computational trajectories demonstrated that the biophysical complexes established between these target ligands and the crystal coordinates of 4EY7 and 2 V5Z maintained excellent structural integrity across the designated simulation timelines. Comprehensive evaluations focusing on root-mean-square deviation (RMSD) and root-mean-square fluctuation (RMSF), along with the time-dependent contact networks of specific amino acid residues, are systematically documented in Figure 7–10 and Tables 6 and 7.

**Figure 7:**
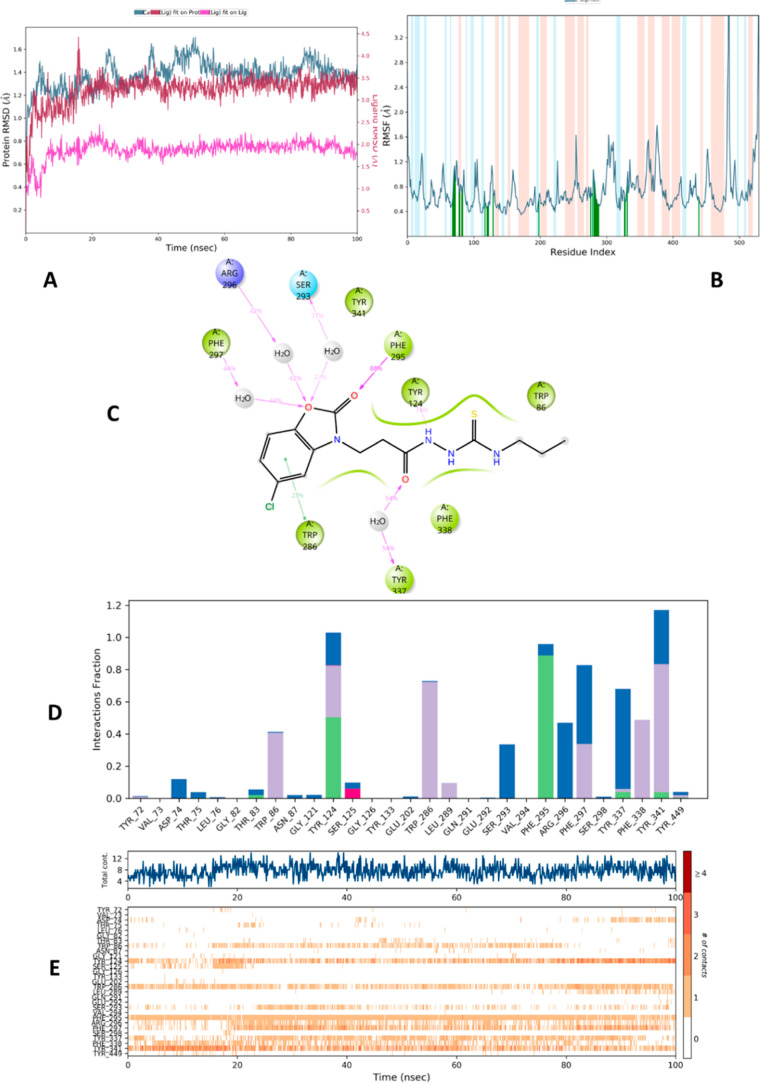
Molecular dynamic results (A–E) of the compound **4ac′**–AChE (PDB ID: 4EY7) complex.

**Figure 8:**
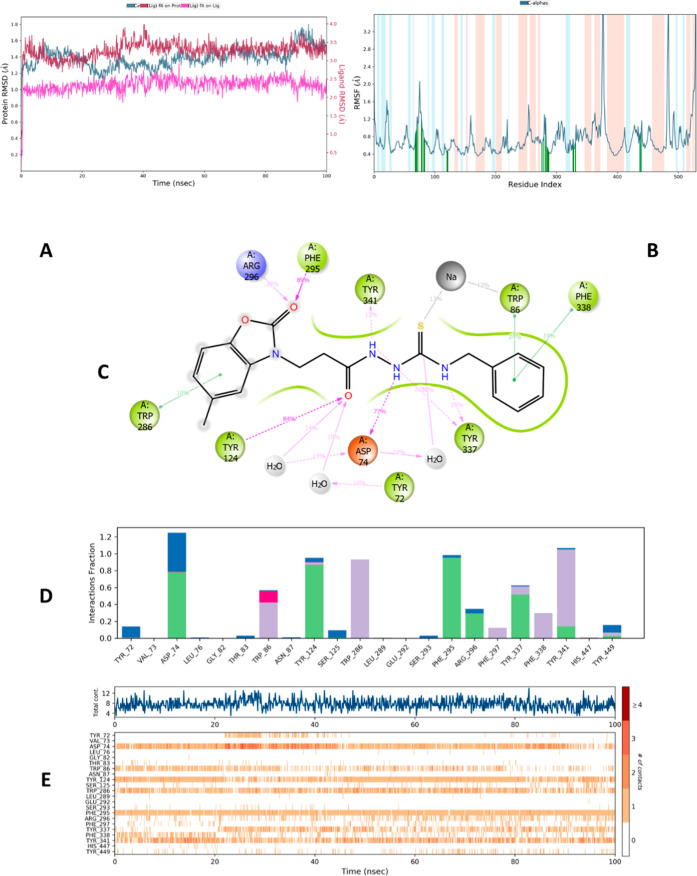
Molecular dynamic results (A–E) of the compound **4bk′**–AChE (PDB ID: 4EY7) complex.

**Figure 9:**
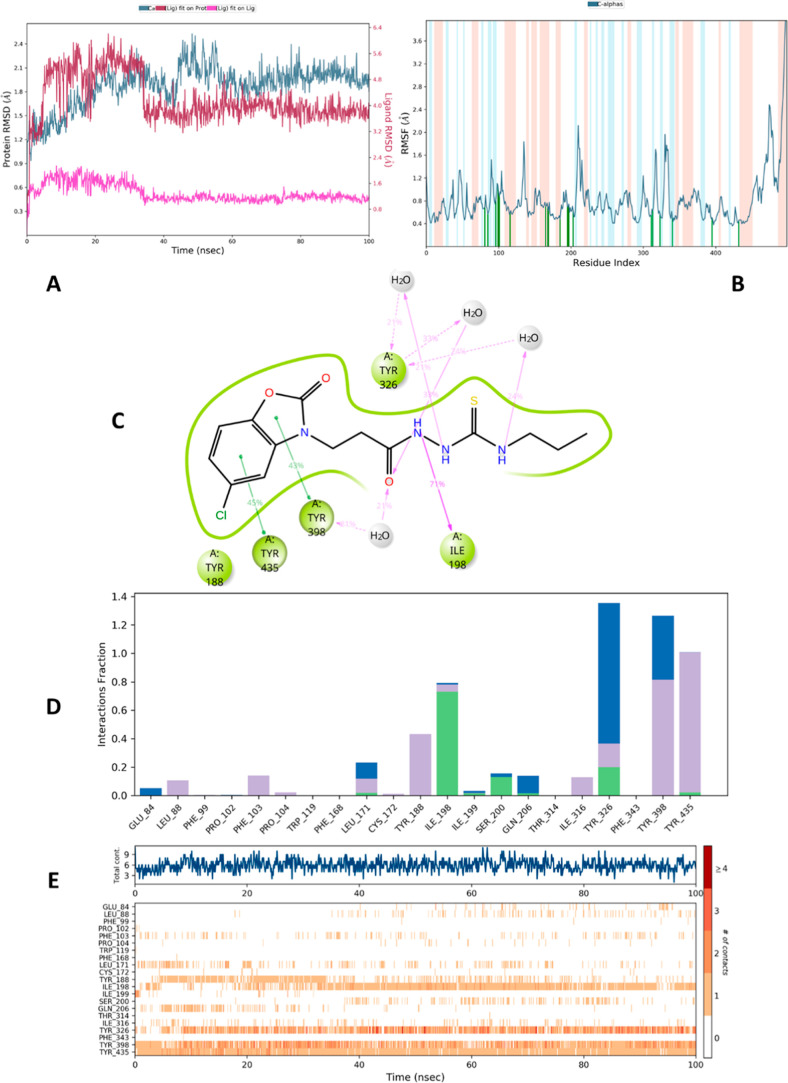
Molecular dynamic results (A–E) of the compound **4ac′**–MAO-B (PDB ID: 2V5Z) complex.

**Figure 10:**
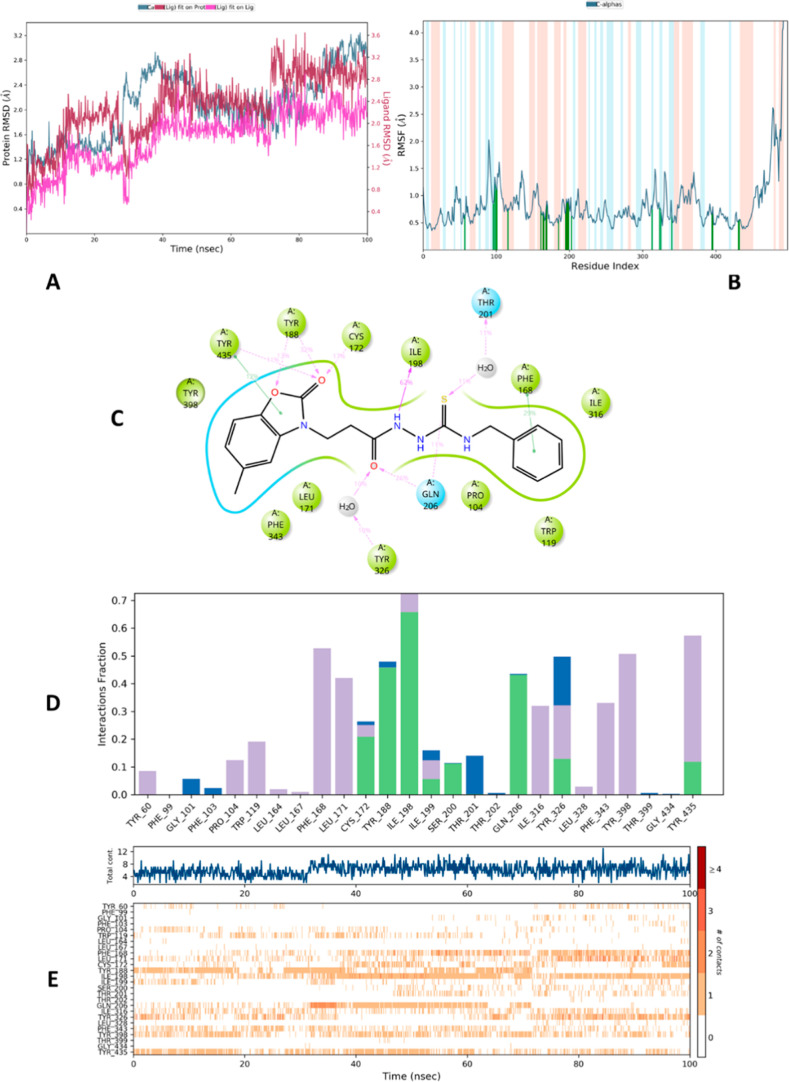
Molecular dynamic results (A–E) of the compound **4bk′**–MAO-B (PDB ID: 2V5Z) complex.

**Table 6:** RMSD, RMSF, and Rg Parameters (Å) for Compounds **4ac′** and **4bk′** on the AChE (PDB ID: 4EY7) Enzyme

complex	RMSD (Å)	*R*_g_	RMSF
**4ac′**-**4EY7**	1.6	4.4–4.8 Å	Tyr 72 (0.59 Å), Val73 (0.80 Å), Asp74 (1.05 Å), Thr75 (1.08 Å), Leu76 (0.91 Å), Gly82 (0.79 Å), Thr83 (0.70 Å), Trp86 (0.68 Å), Asn87 (0.80 Å), Gly121 (0.52 Å), Tyr124 (0.48 Å), Ser125 (0.47 Å), Gly126 (0.54 Å), Tyr133 (0.68 Å), Glu202 (0.60 Å), Trp286 (0.79 Å), Leu289 (0.69 Å), Gln291 (0.94 Å), Glu292 (0.86 Å), Ser293 (0.71 Å), Val294 (0.60 Å), Phe295 (0.59 Å), Arg296 (0.52 Å), Phe297 (0.52 Å), Ser298 (0.52 Å), Tyr337 (0.69 Å), Phe338 (0.56 Å), Tyr341 (0.85 Å), Tyr449 (0.62 Å)
**4bk′**-**4EY7**	1.5	4.2–4.8 Å	Tyr 72 (0.86 Å), Val73 (0.91 Å), Asp74 (0.98 Å), Leu76 (1.06 Å), Gly82 (1.26 Å), Thr83 (0.98 Å), Trp86 (0.74 Å), Asn87 (0.77 Å), Tyr124 (0.46 Å), Ser125 (0.49 Å), Trp286 (0.63 Å), Leu289 (0.82 Å), Glu292 (1.24 Å), Ser293 (0.57 Å), Phe295 (0.54 Å), Arg296 (0.51 Å), Phe297 (0.45 Å), Tyr337 (0.74 Å), Phe338 (0.55 Å), Tyr341 (0.68 Å), His447 (0.66 Å)

**Table 7:** RMSD, RMSF, and *R*_g_ Parameters (Å) for Compounds **4ac′** and **4bk′** on the MAO-B (PDB ID: 2V5Z) Enzyme

complex	RMSD (Å)	*R*_g_	RMSF
**4ac′**-**2 V5Z**	2.1	4.1–4.8 Å	Glu84 (0.69 Å), Leu88 (0.57 Å), Phe99 (0.81 Å), Pro102 (1.07 Å), Phe103 (0.93 Å), Pro104 (0.88 Å), Trp119 (0.60 Å), Phe168 (0.70 Å), Leu171 (0.70 Å), Cys172 (0.63 Å), Tyr188 (0.48 Å), Ile198 (0.70 Å), Ile199 (0.70 Å), Ser200 (0.74 Å), Gln206 (0.63 Å), Thr314 (0.55 Å), Ile316 (0.63 Å), Tyr326 (0.58 Å), Phe343 (0.52 Å), Tyr398 (0.51 Å), Tyr435 (0.46 Å)
**4bk′**-**2 V5Z**	2.8	4.4–5.2 Å	Tyr60 (0.65 Å), Phe99 (0.85 Å), Gly101 (1.34 Å), Phe103 (1.10 Å), Pro104 (1.16 Å), Trp119 (0.77 Å), Leu164 (0.70 Å), Leu167 (0.66 Å), Phe168 (0.66 Å), Leu171 (0.76 Å), Cys172 (0.90 Å), Tyr188 (0.57 Å), Ile198 (0.88 Å), Ile199 (0.94 Å), Ser200 (0.87 Å), Thr201 (0.88 Å), Thr202 (0.94 Å), Gln206 (0.62 Å), Ile316 (0.76 Å), Tyr326 (0.79 Å), Leu328 (0.74 Å), Phe343 (0.66 Å), Tyr398 (0.67 Å), Thr399 (0.79 Å), Gly434 (0.54 Å), Tyr435 (0.56 Å)

To assess the conformational endurance of the engineered ligand–enzyme systems, both RMSD and RMSF profiles were comprehensively evaluated. In computational simulations, RMSD trajectories remaining below 3.0 Å are universally accepted as a hallmark of structural equilibrium; notably, the AChE-bound complexes of derivatives **4ac′** and **4bk′** yielded highly favorable deviations of 1.6 Å and 1.5 Å, respectively (Figures 7A and 8A). Along the same line, the calculated RMSD values for the human MAO-B complexes converged at 2.1 Å and 2.8 Å for the corresponding hybrids (Figures 9A and 10A). These quantitative metrics firmly indicate that both target ligands preserved robust, well-stabilized poses within the active pockets of their respective enzymes over the course of the 100 ns computational timelines. Furthermore, the localized atomic displacements representing the **4ac′**-4EY7, **4bk′**-4EY7, **4ac′**-2 V5Z, and **4bk′**-2 V5Z simulation setups are graphically mapped via their respective RMSF plots in Figures 7B, 8B, 9B, and 10B. In the structural plots, the beta-helices are represented by blue indicators, whereas the α-helical segments are distinguished by pink regions. Within specific domains, an RMSF trajectory surpassing the 1 Å threshold typically reflects local flexibility or a potential conformational adjustment of the bound ligand. The unassigned loop regions are highlighted in white, defining flexible zones that accommodate the structural oscillations of the corresponding derivatives. A comprehensive breakdown of the precise amino acid fluctuations computed for the **4ac**′ + 4EY7 and **4bk**′ + 4EY7 complexes is cataloged in Table 6, while the corresponding data for the **4ac**′ + 2V5Z and **4bk**′ + 2V5Z systems are detailed in Table 7. Based on these fluctuation profiles, only a minimal subset of amino acid residues displayed displacements greater than 1 Å, thereby reinforcing the view that the evaluated candidates preserve high structural rigidity and stable anchoring within the active gorges of both AChE and MAO-B. Additionally, the radius of gyration (rGyr)—a critical biophysical metric utilized in computational trajectories to monitor macromolecular compactness—was meticulously tracked for these complexes to assess their dimensional stability and time-dependent geometric transitions. The time-dependent rGyr trajectories computed for the **4ac′**-4EY7, **4bk′**-4EY7, **4ac′**-2 V5Z, and **4bk′**-2 V5Z systems are graphically displayed in Figures S67–S70 of the Supporting Information. On the whole, the rGyr profiles for these targeted complexes exhibited a steady-state equilibrium across the simulation timelines, consistently varying within a narrow window of 4.0–5.0 Å (Tables 6 and 7). Typically, a minor initial fluctuation was succeeded by a clear convergence toward minimized values, implying that the bound ligands adjusted into more tightly packed and energetically favorable geometries as the trajectories advanced. This characteristic trend strongly supports the absence of any major structural unfolding or macromolecular denaturation within the active domains of the **4ac′**-4EY7, **4bk′**-4EY7, **4ac′**-2 V5Z, and **4bk′**-2 V5Z complexes.

The 2D molecular contact networks established by derivatives **4ac′** and **4bk′** inside the acetylcholinesterase-binding pocket are visually mapped in Figures 7C and 8C, whereas Figures 9C and 10C display their corresponding binding modes within the MAO-B active cleft. A comprehensive compilation documenting all identified noncovalent contacts is systematically cataloged in Tables S1 and S2 of the Supporting Information. An evaluation of the AChE-bound trajectories demonstrated that both hybrids successfully anchored onto critical catalytic and peripheral residues, most notably Trp86, Trp286, Ser293, Phe295, and Arg296. In a similar fashion, the computational interaction profiles derived for the MAO-B complexes confirmed that the ligands aligned closely with key amino acid features including Phe168, Ile198, Tyr398, and Tyr435, all of which are widely acknowledged as pivotal for substrate recognition and enzymatic function.

The distribution of molecular contacts monitored over the computational timelines is graphically categorized in Figures 7D, 8D, 9D, and 10D based on specific binding categories: water-bridged hydrogen bonds (highlighted in blue), direct hydrogen-bonding networks (represented in green), electrostatic/ionic forces (denoted in pink), and hydrophobic stabilization paths (indicated in purple). These molecular histograms clearly elucidate the major noncovalent anchors established between derivatives **4ac′** and **4bk′** and the pivotal residues lining the catalytic cavities of both target enzymes. The precise quantitative metrics underpinning these graphical timelines are systematically organized in Tables S1 and S2 of the Supporting Information. Taken together, these sustained contact pathways robustly reinforce the overall structural integration and highly favorable thermodynamic equilibrium of the AChE and MAO-B complexes formed by hybrids **4ac′** and **4bk′**.

The total count of molecular contacts along with the corresponding time-dependent residue contact frequencies across the computational trajectories are illustrated in Figures 7E, 8E, 9E, and 10E. A detailed inspection of Figures 7E and 8E, which describe the **4ac′**-4EY7 and **4bk′**-4EY7 systems, respectively, demonstrated that these derivatives maintained persistent anchors with essential amino acid features within the AChE catalytic gorge, most notably Asp74, Trp86, Tyr124, Trp286, Phe295, Arg296, Phe297, Tyr337, Phe338, and Tyr341. This continuous contact pattern serves as strong evidence that the hybrids preserved a well-stabilized binding pose inside the active cavity throughout the trajectory. Parallel to these observations, Figures 9E and 10E, representing the **4ac′** and **4bk′** simulations inside the human MAO-B pocket, highlighted uninterrupted networks with critical residues including Tyr188, Ile198, Ile199, Tyr326, Tyr398, and Tyr435. The highly reproducible and uninterrupted nature of these contacts firmly reflects the conformational integrity of both **4ac′** and **4bk′** within the enzymatic cleft, further substantiating their capability as potent MAO-B blocking agents.

In conclusion, the 100 ns MDSs of the **4ac′**–**4EY7**, **4bk′**–**4EY7**, **4ac′**–**2V5Z**, and **4bk′**–**2V5Z** complexes provided detailed insights into the time-dependent stability of the ligand–enzyme interactions. Beyond maintaining the overall structural integrity, the trajectories revealed that critical contacts, particularly with Trp86 and Tyr124 in AChE and Ile198 in MAO-B, exhibited high occupancy rates throughout the simulation period. The RMSF profiles, predominantly below 1 Å for active site residues, indicate that the ligands effectively restrict the conformational flexibility of the binding pockets, thereby stabilizing the complex in a bioactive state. Furthermore, the stable rGyr values (4.0–5.2 Å) demonstrate that the ligands adopt a compact and consistent orientation within the enzymatic gorge, preventing solvent exposure of the hydrophobic core. These findings suggest that **4ac′** and **4bk′** are not only initially well-docked but also maintain the requisite geometric alignment for sustained enzyme inhibition over time.

Taken together, these computational insights underscore that hybrids **4ac′** and **4bk′** not only establish highly effective initial docking interactions but also preserve robust conformational integrity throughout the dynamic trajectories, thereby cementing their capability as prospective dual-acting inhibitors. While the present MD simulations focused on the most potent dual and triple inhibitors to confirm their binding stability, further computational studies involving a broader set of analogues—including those with weaker or more selective profiles—could provide additional insights into the specific energetic penalties and missing interactions that drive isoform selectivity. Nevertheless, the current 100 ns trajectories sufficiently validate the robust binding modes of the lead candidates, providing a reliable structural template for the future optimization of the benzoxazolinone–thiosemicarbazide scaffold. The molecules identified in this study are thought to have the potential to pave the way for further research in the treatment of Alzheimer’s disease through enzyme inhibition.

## Conclusions

This study identified benzoxazolinone–thiosemicarbazide hybrids as promising multitarget candidates for AD-relevant enzyme modulation. Several analogues delivered near-reference AChE potency (e.g., **4af′**, **4bk′**) and MAO-B inhibition close to selegiline (e.g., **4ag′**, **4ac′**, **4bk′**) while maintaining sub-0.10 μM BChE activity in selected cases (e.g., **4af′**, **4bk′**, **4ak′**, **4bj′**, **4bf′**). These findings substantiated the MTDL concept advanced in the introduction, addressing both cholinergic dysfunction and MAO-linked oxidative pathways.

From an SAR perspective, a 5-chloro core generally favored AChE and MAO-B potency over 5-methyl, with notable exceptions (e.g., the benzyl pair); propyl emerged as an optimal aliphatic N^4^-substituent, whereas increased bulk (cyclohexyl, butyl) penalizes activity; and para-substitution on N^4^-phenyl can be used to tune selectivity, with *para*-chloro inducing MAO-B selectivity in the Cl series and *para*-methoxy boosting AChE in the CH_3_ series. In summary, **4bk**, **4ac′**, and **4af′** stand out as lead MTDLs, while **4ag**′ offers a MAO-B-selective chemotype. These tunable features provide clear handles for the next design cycle. Orthogonal evidence absence of H9c2 toxicity at 100 μM, robust antioxidant readouts, stable MD trajectories for dual-actives (RMSD ≤∼3 Å), and ADME compliance supports advancement.

The present results derive from in vitro enzyme assays which are necessary but not sufficient to establish disease-modifying potential. Kinetic mechanisms (reversible vs time-dependent; competitive vs mixed), copper–iron chelation readouts, Aβ aggregation assays, and BBB permeability remain to be defined for the top candidates.

## Experimental Section

### Chemistry

Acetone and dimethylformamide were purchased from Merck (Darmstadt, Germany). Ethanol and methanol were obtained from ISOLAB (Istanbul, Türkiye), and ethyl 3-bromopropionate was supplied by BLDpharm (Shanghai, China). All other chemicals were sourced from Sigma-Aldrich (St. Louis, MO, USA). Melting points were determined using a Thomas-Hoover Capillary Melting Point Apparatus (Thomas Scientific, USA), and the values reported are uncorrected. Infrared (IR) spectra (KBr) were recorded on a PerkinElmer Fourier transform infrared System Spectrum BX (PerkinElmer Inc., USA). Nuclear magnetic resonance (NMR) spectra were acquired at room temperature on a Bruker DPX 300 NMR spectrometer (Billerica, MA, USA) and Bruker Biospin 500 MHz spectrometer (Bruker BioSpin GmbH, Germany) in DMSO-*d*_6_. Chemical shifts (δ) are reported in parts per million (ppm), and coupling constants (J) are given in Hertz (Hz). The NMR spectra are provided in the Supporting Information. High-resolution mass spectra (HRMS) were obtained using a Bruker Daltonics micrOTOF-Q mass spectrometer (Bruker, Billerica, MA, USA) equipped with an electrospray ionization (ESI) source.

### Synthesis of Compounds

The synthesis of starting material **1b**,^45^ intermediates **2** *a*/**2b**, **3** *a*/**3b**,^46,47^ and final compounds (**4aa′**–**4bk′**)^47^ was carried out according to the literature and is schematized in Scheme 1. The other starting material **1a** was purchased. Data on the structural characterization and purity control of the resulting products (**4aa′**–**4bk′**) are presented in the Supporting Information. Full spectroscopic data (^1^H NMR, ^13^C NMR, IR, and HRMS spectra) for all synthesized compounds are provided in the Supporting Information file.

#### *N*-Ethyl-1-[3-(5-chloro-2-benzoxazolinon-3-yl)­propionyl]­thiosemicarbazide (**4aa′**)

Yield: 59.65%; recrystallized from ethanol, mp 178–180 °C (dec.). **IR** (**ATR**, **cm** ^–**1**^), 3299 (NH_stretch_), 3160, 3044 (C–H_stretch_, aromatic), 2971 (C–H_stretch_, aliphatic), 1764 (C=O_stretch_, lactam), 1703 (C=O_stretch_, carbazide), 1615, 1549 (C=C_stretch_ and C=N_stretch_), 1375 (C=S_stretch_), 1248 (C–O_stretch_), 1049, 1007, 963, 795 (C–H_deformation_), ^**1**^ **H NMR** (**500 MHz**, **DMSO**-***d***_**6**_) **δ**: 0.99 (t, 3H, **CH**_**3**_), 2.64 (t, 2H, N–CH_2_–**CH**_**2**_), 3.40 (m, 2H, NH–**CH**_**2**_–CH_3_), 4.04 (t, 2H, N–**CH**_**2**_–CH_2_), 7.16 (dt, 1H, ArH, J: 8.5 Hz and J: 2.5 Hz), 7.35 (d, 1H, ArH, J:8.5 Hz), 7.45 (d, 1H, ArH, J:2.5 Hz), 7.85 (s, CS–**NH**–CH_2_−), 9.10, (s, CO–**NH**–NH–CS), 9.79 (s, CO–NH–**NH**–CS). ^**13**^ **C NMR** (**100 MHz**, **DMSO**-***d***_**6**_) **δ**: 14.8 (**C**H_3_), 22.3, 31.5 (CH_2_–**C**H_2_–CO), 38.6 (**C**H_2_–CH_2_–CO), 110.4, 111.2, 128.4, 132.8, 141.2 (Ar–C), 154.1 (**C**=O), 169.8 (CH_2_–**C**O–NH), 181.6 (C=S). **HRMS** calcd for C_13_H_15_ClN_4_O_3_S [M + H]^+^: *m*/*z*, 343.0632; found, 343.0698.

#### N-Allyl-1-[3-(5-chloro-2-benzoxazolinon-3-yl)­propionyl]­thiosemicarbazide (**4ab′**)

Yield: 68.43%; recrystallized from ethanol, mp 108–110 °C (dec.). **IR** (**ATR**, **cm** ^–**1**^), 3540, 3440, 3319 (NH_stretch_), 3161, (C–H_stretch_, aromatic), 2987 (C–H_stretch_, aliphatic), 1766 (C=O_stretch_, lactam), 1691 (C=O_stretch_, carbazide), 1610, 1545 (C=C_stretch_ and C=N_stretch_), 1373 (C=S_stretch_), 1252 (C–O_stretch_), 1063, 1004, 970 (C–H_deformation_), ^**1**^ **H NMR** (**500 MHz**, **DMSO**-***d***_**6**_) **δ**: 2.65 (t, 2H, N–CH_2_–C**H**_2_), 4.02–4.06 (m, 4H, N–**CH**_2_–CH_2_ and NH–**CH**_**2**_–CH=CH_2_), 4.99–5.08 (m, 2H, =CH_2_), 5.73–5.78 (m, 1H, CH_2_–**CH**=CH_2_), 7.16 (dt, 1H, ArH, J: 8.5 Hz and J: 2.5 Hz), 7.35 (d, 1H, ArH, J:8.5 Hz), 7.45 (d, 1H, ArH, J:2.5 Hz), 8.05 (s, CS–**NH**–CH_2_−), 9.22 (s, CO–**NH**–NH–CS), 9.84 (s, CO–NH–**NH**–CS). ^**13**^ **C NMR** (**100 MHz**, **DMSO**-***d***_**6**_) **δ**: 31.5 (CH_2_–**C**H_2_–CO), 38.5 (**C**H_2_–CH_2_–CO), 46.2 (NH–**C**H_2_), 110.4, 111.2, 115.6, 122.1, 128.4, 132.8, 135.2, 141.2 (Ar–C), 154.1 (**C**=O), 169.8 (CH_2_–**C**O–NH), 181.2 (C=S). **HRMS** calcd for C_14_H_15_ClN_4_O_3_S [M + H]^+^: *m*/*z*, 355.0632; found, 355.0697.

#### *N*-Propyl-1-[3-(5-chloro-2-benzoxazolinon-3-yl)­propionyl]­thiosemicarbazide (**4ac′**)

Yield: 65.41%; recrystallized from ethanol, mp 166–168 °C (dec.), water solubility <5 mg/mL. **IR** (**ATR**, **cm** ^–**1**^), 3346 (NH_stretch_), 1772 (C=O_stretch_, lactam), 1673 (C=O_stretch_, carbazide), 1565, 1499 (C=C_stretch_ and C=N_stretch_), 1370 (C=S_stretch_), 1209 (C–O_stretch_), 1089, 1002 (C–H_deformation_), ^**1**^ **H NMR** (**500 MHz**, **DMSO**-***d***_**6**_) **δ**: 0,78 (t, 3H, NH–CH_2_–CH_2_–**CH**_**3**_), 1.39–1.44 (m, 2H, NH–CH_2_–**CH**_**2**_–CH_3_), 2.64 (t, 2H, N–CH_2_–**CH**_**2**_), 3.40 (m, 2H, NH–**CH**_**2**_–CH_2_–CH_3_), 4.04 ppm (t, 3H, N–**CH**_**2**_–CH_2_), 7.16 (dt, 1H, ArH, J: 8.5 Hz and J: 2.5 Hz), 7.35 (d, 1H, ArH, J:8.5 Hz), 7.45 (d, 1H, ArH, J:2.5 Hz), 7.83 (s, CS–**NH**–CH_2_−), 9.08, (s, CO–**NH**–NH–CS), 9.79 (s, CO–NH–**NH**–CS). ^**13**^ **C NMR** (**100 MHz**, **DMSO**-***d***_**6**_) **δ**: 11.5 (**C**H_3_), 22.3 (**C**H_2_–CH_3_), 31.5 (CH_2_–**C**H_2_–CO), 38.6 (**C**H_2_–CH_2_–CO), 45.7 (NH–**C**H_2_), 110.4, 111.2, 128.4, 132.8, 141.2 (Ar–C), 154.1 (**C**=O), 169.8 (CH_2_–**C**O–NH), 181.8 (C=S). **HRMS** calcd for C_14_H_17_ClN_4_O_3_S [M + H]^+^: *m*/*z*, 357.0788; found, 357.0855.

#### *N*-Butyl-1-[3-(5-chloro-2-benzoxazolinon-3-yl)­propionyl]­thiosemicarbazide (**4ad′**)

Yield: 60.10%; recrystallized from isopropanol, mp 186–189 °C (dec.). **IR** (**ATR**, **cm** ^–**1**^), 3531, 3324 (NH_stretch_), 3160 (C–H_stretch_, aromatic), 2958 (C–H_stretch_, aliphatic), 1767 (C=O_stretch_, lactam), 1688 (C=O_stretch_, carbazide), 1609, 1545 (C=C_stretch_ and C=N_stretch_), 1375 (C=S_stretch_), 1251 (C–O_stretch_), 1061, 970 (C–H_deformation_), ^**1**^ **H NMR** (**500 MHz**, **DMSO**-***d***_**6**_) **δ**: 0.86 (t, 3H, NH–CH_2_–CH_2_–CH_2_–**CH**_**3**_), 1.19–1.24 (m, 2H, NH–CH_2_–CH_2_–**CH**_**2**_–CH_3_), 1.37–1.40 (m, 2H, NH–CH_2_–**CH**_**2**_–CH_2_–CH_3_), 2.64 (t, 3H, N–CH_2_–**CH**_**2**_), 3.34–3.39 (m, 2H, NH–**CH**_**2**_–CH_2_–CH_2_–CH_3_), 4.04 (t, 2H, N–**CH**_**2**_–CH_2_), 7.16 (dt, 1H, ArH, J: 8.5 Hz and J: 2.5 Hz), 7.35 (d, 1H, ArH, J:8.5 Hz), 7.45 (d, 1H, ArH, J:2.5 Hz), 7.81 (s, −CS–**NH**–CH_2_−), 9.08 (s, CO–**NH**–NH–CS), 9.77 (s, CO–NH–**NH**–CS). ^**13**^ **C NMR** (**100 MHz**, **DMSO**-***d***_**6**_) **δ**: 14.2 (**C**H_3_), 19.8 (**C**H_2_–CH_3_), 31.2 (CH_2_–**C**H_2_–CO), 38.6 (**C**H_2_–CH_2_–CO), 43.7 (NH–**C**H_2_), 110.4, 111.2, 128.4, 132.8, 141.2 (Ar–C), 154.1 (**C**=O), 169.8 (CH_2_–**C**O–NH), 181.8 (C=S). **HRMS** calcd for C_15_H_19_ClN_4_O_3_S [M + H]^+^: *m*/*z*, 371.0945; found, 371.1011.

#### *N*-Cyclohexyl-1-[3-(5-chloro-2-benzoxazolinon-3-yl)­propionyl]­thiosemicarbazide (**4ae′**)

Yield: 50.37%; recrystallized from ethanol, mp 187–188 °C (dec.). **IR** (**ATR**, **cm** ^–**1**^), 3312 (NH_stretch_), 1766 (C=O_stretch_, lactam), 1689 (C=O_stretch_, carbazide), 1543, 1481 (C=C_stretch_ and C=N_stretch_), 1373 (C=S_stretch_), 1272 (C–O_stretch_), 1034, 961 (C–H_deformation_). ^**1**^ **H NMR** (**500 MHz**, **DMSO**-***d***_**6**_) **δ**: 1.01–1.69 (m, 11H, CH and CH_2,_ cyclohexyl), 2.64 (t, 3H, N–CH_2_–**CH**_**2**_), 4.03 (t, 2H, N–**CH**_**2**_–CH_2_), 7.15 (dt, 1H, ArH, J: 8.5 Hz and J: 2.5 Hz), 7.35 (d, 1H, ArH, J:8.5 Hz), 7.44 (d, 1H, ArH, J:2.5 Hz), 9.04 (s, CO–**NH**–NH–CS), 9.74 (s, CO–NH–**NH**–CS). ^**13**^ **C NMR** (**100 MHz**, **DMSO**-***d***_**6**_) **δ**: 25.3, 25.6, 31.6 (CH_2_–**C**H_2_–CO), 32.1, 38.6 (**C**H_2_–CH_2_–CO), 53.2 (NH–**C**H), 110.5, 111.1, 122.0, 128.4, 132.8, 141.2 (Ar–C), 154.1 (**C**=O), 169.7 (CH_2_–**C**O–NH), 181.7 (C=S). **HRMS** calcd for C_17_H_21_ClN_4_O_3_S [M + H]^+^: *m*/*z*, 397.1101; found, 397.1173.

#### *N*-Phenyl-1-[3-(5-chloro-2-benzoxazolinon-3-yl)­propionyl]­thiosemicarbazide (**4af′**)

Yield: 58.33%; recrystallized from ethanol, mp 170 °C (dec.), water solubility <5 mg/mL. **IR** (**ATR**, **cm** ^–**1**^), 3320 (NH_stretch_), 3164 (C–H_stretch_, aromatic), 2945 (C–H_stretch_, aliphatic), 1769 (C=O_stretch_, lactam), 1709 (C=O_stretch_, carbazide), 1618, 1546 (C=C_stretch_ and C=N_stretch_), 1370 (C=S_stretch_), 1252 (C–O_stretch_), 1064, 982 (C–H_deformation_), ^**1**^ **H NMR** (**500 MHz**, **DMSO**-***d***_**6**_) **δ**: 2.70 (t, 2H, N–CH_2_–**CH**_**2**_), 4.07 (t, 2H, N–**CH**_2_–CH_2_), 7.16–7.18 (m, 2H, ArH), 7.29–7.32 (m, 4H, ArH), 7.35 (d, 1H, ArH, J:8.5 Hz), 7.44 (d, 1H, ArH, J:2.5 Hz), 9.55 (s, 2H, CO–**NH**–NH–CS and CO–NH–**NH**–CS), 10.03 (s, −CS–**NH**–CH_2_). ^**13**^ **C NMR** (**100 MHz**, **DMSO**-***d***_**6**_) **δ**: 31.6 (CH_2_–**C**H_2_–CO), 38.5 (**C**H_2_–CH_2_–CO), 110.5, 111.2, 122.0, 122.1, 125.6, 126.4, 128.4, 128.5, 132.9, 139.4, 141.2 (Ar–C), 154.1 (**C**=O), 170.1 (CH_2_–**C**O–NH), 181.5 (C=S). **HRMS** calcd for C_17_H_15_ClN_4_O_3_S [M + H]^+^: *m*/*z*, 391.0632; found, 391.0697.

#### *N*-(4-Chlorophenyl)-1-[3-(5-chloro-2-benzoxazolinon-3-yl)­propionyl]­thiosemicarbazide (**4ag′**)

Yield: 69.80%; recrystallized from ethanol, mp 207–208 °C (dec.), water solubility <5 mg/mL. **IR** (**ATR**, **cm** ^–**1**^), 3180 (C–H_stretch_, aromatic), 2974 (C–H_stretch_, aliphatic), 1757 (C=O_stretch_, lactam), 1712 (C=O_stretch_, carbazide), 1546, 1487 (C=C_stretch_ and C=N_stretch_), 1376 (C=S_stretch_), 1185 (C–O_stretch_), 1088, 1047, 966, 833, 749 (C–H_deformation_). ^**1**^ **H NMR** (**500 MHz**, **DMSO**-***d***_**6**_) **δ**: 2.70 (t, 2H, N–CH_2_–**CH**_**2**_), 4.07 (t, 2H, N–**CH**_2_–CH_2_), 7.16 (dt, 1H, ArH), 7.33–7.36 (m, 5H, ArH), 7.48 (d, 1H, ArH, J:2.5 Hz), 9.41–9.64 (s, 2H, CO–**NH**–NH–CS and CO–NH–**NH**–CS), 10.03 (s, −CS–**NH**–CH_2_). ^**13**^ **C NMR** (**100 MHz**, **DMSO**-***d***_**6**_) **δ**: 31.6 (CH_2_–**C**H_2_–CO), 38.5 (**C**H_2_–CH_2_–CO), 110.5, 111.2, 122.0, 122.1, 127.9, 128.4, 129.7, 132.9, 138.4, 141.2 (Ar–C), 154.1 (**C**=O), 170.0 (CH_2_–**C**O–NH), 181.2 (C=S). **HRMS** calcd for C_17_H_14_Cl_2_N_4_O_3_S [M + H]^+^: *m*/*z*, 425.0242; found, 425.0308.

#### *N*-(4-Methylphenyl)-1-[3-(5-chloro-2-benzoxazolinon-3-yl)­propionyl]­thiosemicarbazide (**4ah′**)

Yield: 65.50%; recrystallized from ethanol, mp 196–198 °C (dec.), **IR (ATR, cm** ^**–1**^), 3212 (NH_stretch_), 1761 (C=O_stretch_, lactam), 1647 (C=O_stretch_, carbazide), 1520, 1491 (C=C_stretch_ and C=N_stretch_), 1360 (C=S_stretch_), 1202 (C–O_stretch_), 1060, 1014, 942, 840, 790 (C–H_deformation_), ^**1**^ **H NMR** (**500 MHz**, **DMSO**-***d***_**6**_) **δ**: 2.28 (s, 3H, –CH_3_), 2.69 (t, 2H, N–CH_2_–**CH**_**2**_), 4.07 (t, 2H, N–**CH**_2_–CH_2_), 7.10–7.18 (m, 5H, ArH), 7.34 (d, 1H, ArH, J: 8.5 Hz), 7.47 (d, 1H, ArH, J: 2.5 Hz), 9.48 (s, 2H, CO–**NH**–NH–CS and CO–NH–**NH**–CS), 10.01 (s, −CS–**NH**–CH_2_). ^**13**^ **C NMR** (**100 MHz**, **DMSO**-***d***_**6**_) **δ**: 21.0 (**C**H_3_), 31.5 (CH_2_–**C**H_2_–CO), 38.5 (**C**H_2_–CH_2_–CO), 110.4, 111.2, 122.0, 122.1, 126.3, 128.3, 128.9, 132.9, 134.8, 136.8, 141.2 (Ar–C), 154.1 (**C**=O), 169.9 (CH_2_–**C**O–NH), 181.3 (C=S). **HRMS** calcd for C_18_H_17_ClN_4_O_3_S [M + H]^+^: *m*/*z*, 405.0788; found, 405.0850.

#### *N*-(4-Nitrophenyl)-1-[3-(5-chloro-2-benzoxazolinon-3-yl)­propionyl]­thiosemicarbazide (**4ai′**)

Yield: 67.40%; recrystallized from ethanol, mp 222–224 °C (dec.), **IR** (**ATR**, **cm** ^–**1**^); 3330, 3271 (NH_stretch_), 1770 (C=O_stretch_, lactam), 1643 (C=O_stretch_, carbazide), 1598, 1482 (C=C_stretch_ and C=N_stretch_), 1375 (C=S_stretch_), 1226 (C–O_stretch_), 1107, 1051, 966, 807, 746 (C–H_deformation_), ^**1**^ **H NMR** (**500 MHz**, **DMSO**-***d***_**6**_) **δ**: 2.73 (t, 2H, N–CH_2_–**CH**_**2**_), 4.08 (t, 2H, N–**CH**_2_–CH_2_), 7.12–7.16 (m, 1H, ArH), 7.34 (d, 1H, ArH, J: 8.5 Hz), 7.44–7.48 (m, 1H, ArH), 7.73–7.88 (m, 2H, ArH), 8.18 (d, 2H, ArH, J: 9 Hz), 9.82–10.10 (s, 3H, CO–**NH**–NH–CS and CO–NH–**NH**–CS and CS–**NH**–CH_2_), ^**13**^ **C NMR** (**100 MHz**, **DMSO**-***d***_**6**_) **δ**: 31.6 (CH_2_–**C**H_2_–CO), 38.5 (**C**H_2_–CH_2_–CO), 110.4, 111.2, 122.0, 124.0, 125.0, 128.3, 132.8, 134.8, 136.8, 141.2, 145.9 (Ar–C), 154.1 (**C**=O), 170.2 (CH_2_–**C**O–NH), 180.9 (C = S). **HRMS** calcd for C_17_H_14_ClN_5_O_5_S [M + H]^+^: *m*/*z*, 436.0482; found, 436.0542.

#### *N*-(4-Methoxyphenyl)-1-[3-(5-chloro-2-benzoxazolinon-3-yl)­propionyl]­thiosemicarbazide (**4aj′**)

Yield: 70%; recrystallized from ethanol, mp 178–180 °C (dec.), **IR** (**ATR**, **cm** ^–**1**^), 3255 (NH_stretch_), 3069 (C–H_stretch_, aromatic), 2884 (C–H_stretch_, aliphatic), 1781 (C=O_stretch_, lactam), 1680 (C=O_stretch_, carbazide), 1525, 1513 (C=C_stretch_ and C=N_stretch_), 1370 (C=S_stretch_), 1179 (C–O_stretch_), 1041, 1022, 960, 802, 745 (C–H_deformation_), ^**1**^ **H NMR** (**500 MHz**, **DMSO**-***d***_**6**_) **δ**: 2.69 (t, 2H, N–CH_2_–**CH**_**2**_), 3.75 (s, 3H, –OCH_3_), 4.06 (t, 2H, N–**CH**_2_–CH_2_), 6.86 (d, 1H, ArH, J: 9 Hz), 7.14–7.18 (m, 3H, ArH), 7.35 (d, 1H, ArH, J: 8.5 Hz), 7.47 (d, 1H, ArH, J: 2.5 Hz), 9.42–9.45 (m, 2H, CO–**NH**–NH–CS and CO–NH–**NH**–CS), 9.99 (s, −CS–**NH**–CH_2_). ^**13**^ **C NMR** (**100 MHz**, **DMSO**-***d***_**6**_) **δ**: 31.5 (CH_2_–**C**H_2_–CO), 38.5 (**C**H_2_–CH_2_–CO), 55.6 (OCH_3_), 110.4, 111.2, 113.7, 122.1, 127.9, 128.3, 132.9, 141.2, 157.3 (Ar–C), 154.1 (**C**=O), 169.9 (CH_2_–**C**O–NH), 181.6 (C=S). **HRMS** calcd for C_18_H_17_ClN_4_O_4_S [M + H]^+^: *m*/*z*, 421.0737; found, 421.0802.

#### *N*-Benzyl-1-[3-(5-chloro-2-benzoxazolinon-3-yl)­propionyl]­thiosemicarbazide (**4ak′**)

Yield: 58.60%; recrystallized from ethanol, mp 232–233 °C (dec.), **IR** (**ATR**, **cm** ^–**1**^), 3201 (NH_stretch_), 1770 (C=O_stretch_, lactam), 1687 (C=O_stretch_, carbazide), 1552, 1484 (C=C_stretch_ and C=N_stretch_), 1371 (C=S_stretch_), 1199 (C–O_stretch_), 1063, 1044, 957, 840, 796 (C–H_deformation_), ^**1**^ **H NMR** (**500 MHz**, **DMSO**-***d***_**6**_) **δ**: 2.66 (t, 2H, N–CH_2_–**CH**_**2**_), 4.04 (t, 2H, N–**CH**_2_–CH_2_), 4.70 (d, NH–**CH**_**2**_), 7.17 (dd, 1H, ArH, J: 8.5 Hz, J: 2.5 Hz), 7.20–7.33 (m, 5H, ArH), 7.35 (d, 1H, ArH, J: 8.5 Hz), 7.47 (d, 1H, ArH, J: 2.5 Hz), 8.43 (s, CO–**NH**–NH–CS), 9.31 (s, −CS–**NH**–CH_2_), 9.91 (s, CO–NH–**NH**–CS). ^**13**^ **C NMR** (**100 MHz**, **DMSO**-***d***_**6**_) **δ**: 31.5 (CH2–**C**H_2_–CO), 38.5 (**C**H_2_–CH_2_–CO), 47.1 (CH_2_), 110.4, 111.2, 122.1, 127.0, 127.3, 127.6, 128.4, 128.5, 132.8, 139.6, 141.2 (Ar–C), 154.0 (**C**=O), 169.8 (CH_2_–**C**O–NH), 182.5 (C=S). **HRMS** calcd for C_18_H_17_ClN_4_O_3_S [M + H]^+^: *m*/*z*, 405.0788; found, 405.0849.

#### *N*-Ethyl-1-[3-(5-methyl-2-benzoxazolinon-3-yl)­propionyl]­thiosemicarbazide (**4ak′**)

Yield: 66.20%; recrystallized from ethanol, mp 183 °C (dec.), **IR** (**ATR**, **cm** ^–**1**^), 3289 (NH_stretch_), 3174 (C–H_stretch_, aromatic), 2969 (C–H_stretch_, aliphatic), 1758 (C=O_stretch_, lactam), 1703 (C=O_stretch_, carbazide), 1622, 1572, 1455 (C=C_stretch_ and C=N_stretch_), 1382 (C=S_stretch_), 1243 (C–O_stretch_), 1085, 1009, 934 (C–H_deformation_), ^**1**^ **H NMR** (**500 MHz**, **DMSO**-***d***_**6**_) **δ**: 1.03 (t, 3H, **CH**_**3**_), 2.35 (s, 3H, **CH**_**3**_), 2.63 (t, 2H, N–CH_2_–**CH**_**2**_), 3.42 (m, 2H, NH–**CH**_**2**_–CH_3_), 4.02 (t, 2H, N–**CH**_2_–CH_2_), 6.92 (d, 1H, ArH, J: 8.0), 7.13 (s, 1H, ArH), 7.19 (d, 1H, ArH, J:8 Hz), 7.85 (s, CS-**NH**-CH_2_−), 9.10 (s, CO-**NH**-NH–CS), 9.80 (s, CO-NH-**NH-**CS). ^**13**^ **C NMR** (**100 MHz**, **DMSO**-***d***_**6**_) **δ**: 14.8 (CH_2_–**C**H_3_), 21.5 (**C**H_3_), 31.7 (CH_2_–**C**H_2_–CO), 38.3 (**C**H_2_–CH_2_–CO), 38.8 (NH–**C**H_2_), 109.6, 110.3 (Ar–C), 154.4 (**C**=O), 169.7 (CH_2_–**C**O–NH), 181.6 (C=S). **HRMS** calcd for C_14_H_18_N_4_O_3_S [M + H]^+^: *m*/*z*, 323.1178; found, 323.1231.

#### *N*-Allyl-1-[3-(5-methyl-2-benzoxazolinon-3-yl)­propionyl]­thiosemicarbazide (**4bb′**)

Yield: 54.38%; recrystallized from ethanol, mp 154–155 °C (dec.), **IR** (**ATR**, **cm** ^–**1**^), 3520, 3447 (NH_stretch_), 3160 (C–H_stretch_, aromatic), 2990 (C–H_stretch_, aliphatic), 1741 (C=O_stretch_, lactam), 1691 (C=O_stretch_, carbazide), 1650, 1563, 1446 (C=C_stretch_ and C=N_stretch_), 1380 (C=S_stretch_), 1255 (C–O_stretch_), 1094, 1053, 951 (C–H_deformation_). ^**1**^ **H NMR** (**500 MHz**, **DMSO**-***d***_**6**_) **δ**: 2.36 (s, 3H, **CH**_**3**_), 2.65 (t, 2H, N–CH_2_–**CH**_**2**_), 4.01 ((t, 2H, N–**CH**_2_–CH_2_), 4.07 (s, 2H, NH–**CH**_**2**_), 5.01–5.11 (m, 2H, CH_2_–CH=**CH**_**2**_), 5.76–5.82 (m, 1H, CH_2_–**CH**=CH_2_), 6.92 (d, 1H, ArH, J: 8.0), 7.13 (s, 1H, ArH), 7.19 (d, 1H, ArH, J:8 Hz), 8.06 (s, CS–**NH**–CH_2_−), 9.23 (s, CO–**NH**–NH–CS), 9.85 (s, CO–NH–**NH**–CS). ^**13**^ **C NMR** (**100 MHz**, **DMSO**-***d***_**6**_) **δ**: 21.5 (**C**H_3_), 31.7 (CH_2_–**C**H_2_–CO), 38.2 (**C**H_2_–CH_2_–CO), 46.2 (NH–**C**H_2_), 109.6, 110.3, 122.8, 131.3, 133.7, 140.5 (Ar–C), 115.6 (CH=**C**H_2_), 135.3 (**C**H=CH_2_), 154.3 (**C**=O), 169.8 (CH2–**C**O–NH), 182.1 (C=S). HRMS calcd for C_15_H_18_N_4_O_3_S [M + H]^+^: *m*/*z*, 335.1178; found, 335.1233.

#### *N*-Propyl-1-[3-(5-methyl-2-benzoxazolinon-3-yl)­propionyl]­thiosemicarbazide (**4bc′**)

Yield: 67.16%; recrystallized from ethanol, mp 199–200 °C (dec.), **IR** (**ATR**, **cm** ^–**1**^), 3173 (NH_stretch_), 3012 (C–H_stretch_, aromatic), 2979 (C–H_stretch_, aliphatic), 1762 (C=O_stretch_, lactam), 1716 (C=O_stretch_, carbazide), 1511, 1473 (C=C_stretch_ and C=N_stretch_), 1370 (C=S_stretch_), 1170 (C–O_stretch_), 970, 823, 790 (C–H_deformation_), ^**1**^ **H NMR** (**500 MHz**, **DMSO**-***d***_**6**_) **δ**: 0.81 (t, 3H, NH–CH_2_–CH_2_–**CH**_**3**_), 1.44–1.49 (m, 2H, NH–CH_2_–**CH**_**2**_–CH_3_), 2.36 (s, 3H, **CH**_**3**_), 2.64 (t, 2H, N–CH_2_–**CH**_**2**_), 3.35 (m, 2H, NH–**CH**_**2**_–CH_2_–CH_3_), 4.02 ppm (t, 3H, N–**CH**_**2**_–CH_2_), 6.93 (d, 1H, ArH, J: 8 Hz), 7.10 (s, 1H, ArH), 7.19 (d, 1H, ArH, J: 8 Hz), 7.84 (s, CS–**NH–**CH_2_−), 9.09 and 9.25 (s, CO–**NH–**NH–CS), 9.81 (s, CO–NH–**NH–**CS), ^**13**^ **C NMR (100 MHz, DMSO-*d***_**6**_ **) δ:** 11.5 (CH_2_–**C**H_3_), 21.5 (**C**H_3_), 22.4 (**C**H_2_–CH_3_), 31.7 (CH_2_–**C**H_2_–CO), 38.3 (**C**H_2_–CH_2_–CO), 45.7 (NH–**C**H_2_), 109.6, 110.2, 110.3, 122.8, 122.9, 131.2, 131.3, 133.6, 133.7, 140.5 (Ar–C), 154.4 (**C**=O), 169.7 (CH_2_–**C**O–NH), 181.8 (C=S). **HRMS** calcd for C_15_H_20_N_4_O_3_S [M + H]^+^: *m*/*z*, 337.1334; found, 337.1391.

#### *N*-Butyl-1-[3-(5-methyl-2-benzoxazolinon-3-yl)­propionyl]­thiosemicarbazide (**4bd′**)

Yield: 75%; recrystallized from ethanol, mp 183–184 °C (dec.), **IR (ATR, cm** ^**–1**^ **)**, 3529 (NH_stretch_), 3165 (C–H_stretch_, aromatic), 2989 (C–H_stretch_, aliphatic), 1743 (C=O_stretch_, lactam), 1691 (C=O_stretch_, carbazide), 1622, 1542, 1493, 1445 (C=C_stretch_ and C=N_stretch_), 1379 (C=S_stretch_), 1190 (C–O_stretch_), 1053, 970 (C–H_deformasyon_), ^**1**^ **H NMR (500 MHz, DMSO-*d***_**6**_ **) δ:** 0.87 (t, 3H, NH–CH_2_–CH_2_–CH_2_–**CH**_**3**_), 1.21–1.26 (m, 2H, NH–CH_2_–CH_2_–**CH**_**2**_–CH_3_), 1.41–1.46 (m, 2H, NH–CH_2_–**CH**_**2**_–CH_2_–CH_3_), 2.36 (s, 3H, **CH**_**3**_), 2.64 (t, 3H, N–CH_2_–**CH**_**2**_), 3.40 (m, 2H, NH–**CH**_**2**_–CH_2_–CH_2_–CH_3_), 4.02 (t, 2H, N–**CH**_**2**_–CH_2_), 6.93 (d, 1H, ArH, J: 8 Hz), 7.13 (s, 1H, ArH), 7.20 (d, 1H, ArH, J:8 Hz), 7.81 (s, CS–**NH–**CH_2_−), 9.09 (s, CO–**NH–**NH–CS), 9.80 (s, CO–NH–**NH–**CS). ^**13**^ **C NMR (100 MHz, DMSO-*d***_**6**_ **) δ:** 14.2 (CH_2_–**C**H_3_), 19.8 (**C**H_2_–CH_2_–CH_3_), 21.5 (**C**H_3_), 31.7 (CH_2_–**C**H_2_–CO), 38.3 (**C**H_2_–CH_2_–CO), 43.7 (NH–**C**H_2_), 109.6, 110.3, 110.3, 122.8, 131.3, 133.7, 140.5 (Ar–C), 154.4 (**C**=O), 169.8 (CH_2_–**C**O–NH), 181.7 (C=S). **HRMS** calcd for C_16_H_22_N_4_O_3_S [M + H]^+^: *m*/*z*, 351.1491; found, 351.1549.

#### *N*-Cyclohexyl-1-[3-(5-methyl-2-benzoxazolinon-3-yl)­propionyl]­thiosemicarbazide (**4be′**)

Yield: 66.90%; recrystallized from ethanol, mp 236–237 °C (dec.), **IR (ATR, cm** ^**–1**^ **),** 3339 (NH_stretch_), 3072 (C–H_stretch_, aromatic), 2893 (C–H_stretch_, aliphatic), 1772 (C=O_stretch_, lactam), 1693 (C=O_stretch_, carbazide), 1503, 1480 (C=C_stretch_ and C=N_stretch_), 1376 (C=S_stretch_), 1182 (C–O_stretch_), 996, 903, 771 (C–H_deformation_), ^**1**^ **H NMR (500 MHz, DMSO-*d***_**6**_ **) δ:** 1.07–1.74 (m, 11H, CH and CH_2_, cyclohexyl), 2.35 (s, 3H, **CH**_**3**_), 2.64 (t, 3H, N–CH_2_–**CH**_**2**_), 4.02 (t, 2H, N–**CH**_**2**_–CH_2_), 6.93 (d, 1H, ArH, J: 8.0), 7.13 (s, 1H, ArH), 7.20 (d, 1H, ArH, J:8 Hz), 7.98 (s, CS–**NH–**CH_2_−), 9.05 and 9.19 (s, CO–**NH–**NH–CS), 9.76 (s, CO–NH–**NH–**CS). ^**13**^ **C NMR (100 MHz, DMSO-*d***_**6**_ **) δ:** 21.4 (CH_2_–**C**H_3_), 21.6 (**C**H_2_–CH_3_), 25.3 (CH_2_), 25.5 (CH_2_), 31.8 (CH_2_–**C**H_2_–CO), 38.4 (**C**H_2_–CH_2_–CO), 53.3 (NH–**C**H_2_), 109.5, 109.6, 110.2, 110.4, 122.8, 131.2, 131.3, 133.6, 140.5 (Ar–C), 154.4 (**C**=O), 169.7 (CH_2_–**C**O–NH), 181.8 (C=S). **HRMS** calcd for C_18_H_24_N_4_O_3_S [M + H]^+^: *m*/*z*, 377.1647; found, 377.1712.

#### *N*-Phenyl-1-[3-(5-methyl-2-benzoxazolinon-3-yl)­propionyl]­thiosemicarbazide (**4bf′**)

Yield: 64.35%; recrystallized from ethanol, mp 183–185 °C (dec.), **IR (ATR, cm** ^**–1**^ **),** 3239 (NH_stretch_), 3167 (C–H_stretch_, aromatic), 2976 (C–H_stretch_, aliphatic), 1742 (C=O_stretch_, lactam), 1710 (C=O_stretch_, carbazide), 1522, 1496 (C=C_stretch_ and C=N_stretch_), 1372 (C=S_stretch_), 1257 (C–O_stretch_), 1008, 903, 771 (C–H_deformation_), ^**1**^ **H NMR (500 MHz, DMSO-*d***_**6**_ **) δ:** 2.33 (s, 3H, **CH**_**3**_), 2.67 (t, 2H, N–CH_2_–**CH**_**2**_), 4.03 (t, 2H, N–**CH**_2_–CH_2_), 6.92 (d, 1H, ArH, J: 8 Hz), 7.13–7.39 (m, 7H, ArH), 9.56 (s, CS–**NH–**CH_2_ and CO–**NH–**NH–CS), 10.03 (s, CO–NH–**NH–**CS). ^**13**^ **C NMR (100 MHz, DMSO-*d***_**6**_ **) δ:** 21.5 (**C**H_3_), 31.7 (CH_2_–**C**H_2_–CO), 38.2 (**C**H_2_–CH_2_–CO), 109.6, 110.3, 110.3, 110.4, 122.8, 125.6, 126.5, 128.5, 131.3, 133.6, 139.4, 140.5 (Ar–C), 154.4 (**C**=O), 169.9 (CH_2_–**C**O–NH), 181.3 (C=S). **HRMS** calcd for C_18_H_18_N_4_O_3_S [M + H]^+^: *m*/*z*, 371.1178; found, 371.1240.

#### *N*-(4-Chlorophenyl)-1-[3-(5-methyl-2-benzoxazolinon-3-yl)­propionyl]­thiosemicarbazide (**4bg′**)

Yield: 68.20%; recrystallized from ethanol, mp 215–216 °C (dec.), **IR (ATR, cm** ^**–1**^ **),** 3166 (C–H_stretch_, aromatic), 2972 (C–H_stretch_, aliphatic), 1770 (C=O_stretch_, lactam), 1714 (C=O_stretch_, carbazide), 1549, 1494 (C=C_stretch_ and C=N_stretch_), 1373 (C=S_stretch_), 1187 (C–O_stretch_), 1084, 1013, 972, 838, 796 (C–H_deformation_), ^**1**^ **H NMR (500 MHz, DMSO-*d***_**6**_ **) δ:** 2.35 (s, 3H, **CH**_**3**_), 2.69 (t, 2H, N–CH_2_–**CH**_**2**_), 4.05 (t, 2H, N–**CH**_2_–CH_2_), 6.93 (d, 1H, ArH, J: 8.0), 7.14 (s, 1H, ArH), 7.19 (d, 1H, ArH, J: 8 Hz), 7.37 (s, 4H, ArH), 9.57 and 9.67 (s, CS–**NH–**CH_2_ and CO–**NH–**NH–CS), 10.05 (s, CO–NH–**NH–**CS). ^**13**^ **C NMR (100 MHz, DMSO-*d***_**6**_ **) δ:** 21.5 (CH_3_), 31.7 (CH_2_–**C**H_2_–CO), 38.2 (**C**H_2_–CH_2_–CO), 109.6, 110.3, 122.8, 127.9, 128.3, 131.3, 133.6, 138.5, 140.5 (Ar–C), 154.4 (**C**=O), 170.0 (CH_2_–**C**O–NH), 181.2 (C = S). **HRMS** calcd for C_18_H_17_ClN_4_O_3_S [M + H]^+^: *m*/*z*, 405.0788; found, 405.0856.

#### *N*-(4-Methylphenyl)-1-[3-(5-methyl-2-benzoxazolinon-3-yl)­propionyl]­thiosemicarbazide (**4bh′**)

Yield: 68.55%; recrystallized from ethanol, mp 205–207 °C (dec.), **IR (ATR, cm** ^**–1**^ **),** 3163, 3033 (C–H_stretch_, aromatic), 2969 (C–H_stretch_, aliphatic), 1770 (C=O_stretch_, lactam), 1713 (C=O_stretch_, carbazide), 1538, 1494 (C=C_stretch_ and C=N_stretch_), 1372 (C=S_stretch_), 1177 (C–O_stretch_), 1051, 1012, 970, 820, 790 (C–H_deformation_), ^**1**^ **H NMR (500 MHz, DMSO-*d***_**6**_ **) δ:** 2.28 (s, 3H, CH_3_), 2.35 (s, 3H, **CH**_**3**_), 2.69 (t, 2H, N–CH_2_–**CH**_**2**_), 4.05 (t, 2H, N–**CH**_2_–CH_2_), 6.93 (d, 1H, ArH, J: 8.0), 7.11–7.28 (m, 6H, ArH), 9.49 (s, 2H, CS–**NH**–CH_2_ and CO–**NH**–NH–CS), 10.02 (s, CO–NH–**NH**–CS), ^**13**^ **C NMR (100 MHz, DMSO-*d***_**6**_ **) δ:** 21.0 (CH3), 21.5 (CH_3_), 31.7 (CH_2_–**C**H_2_–CO), 38.2 (**C**H_2_–CH_2_–CO), 109.6, 110.3, 122.8, 126.4, 128.9, 131.4, 133.6, 134.8, 136.9, 140.5 (Ar–C), 154.4 (**C**=O), 169.9 (CH_2_–**C**O–NH), 181.5 (C = S). **HRMS** calcd for C_19_H_20_N_4_O_3_S [M + H]^+^: *m*/*z*, 385.1334; found, 385.1404.

#### *N*-(4-Nitrophenyl)-1-[3-(5-methyl-2-benzoxazolinon-3-yl)­propionyl]­thiosemicarbazide (**4bi′**)

Yield: 61.47%; recrystallized from ethanol, mp 228–230 °C (dec.), **IR (ATR, cm** ^**–1**^ **),** 3330 (NH_stretch_), 3086 (C–H_stretch_, aromatic), 2893 (C–H_stretch_, aliphatic), 1770 (C=O_stretch_, lactam), 1625 (C=O_stretch_, carbazide), 1583, 1499 (C=C_stretch_ and C=N_stretch_), 1324 (C=S_stretch_), 1111 (C–O_stretch_), 1003, 973, 847, 745 (C–H_deformation_), ^**1**^ **H NMR (500 MHz, DMSO-*d***_**6**_ **) δ:** 2.34 (s, 3H, **CH**_**3**_), 2.72 (t, 2H, N–CH_2_–**CH**_**2**_), 4.06 (t, 2H, N–**CH**_2_–CH_2_), 6.90 (d, 1H, ArH, J: 8.0), 7.13 (s, 1H, ArH), 7.19 (d, 1H, ArH, J:8 Hz), 7.79 (s, 2H, ArH), 8.18 (d, 2H, ArH, J: 9 Hz), 9.51–10.11 (s, 3H, CS–**NH–**CH_2_ and CO–**NH–**NH–CS and CO–NH–**NH–**CS), ^**13**^ **C NMR (100 MHz, DMSO-*d***_**6**_ **) δ:** 21.5 (CH_3_), 31.8 (CH_2_–**C**H_2_–CO), 110.3, 122.8, 124.1, 125.1, 131.3, 133.6, 140.5 (Ar–C), 154.4 (**C**=O), 170.1 (CH_2_–**C**O–NH), 180.9 (C=S). **HRMS** calcd for C_18_H_17_N_5_O_5_S [M + H]^+^: *m*/*z*, 416.1029; found, 416.1100.

#### *N*-(4-Methoxyphenyl)-1-[3-(5-methyl-2-benzoxazolinon-3-yl)­propionyl]­thiosemicarbazide (**4bj′**)

Yield: 70.10%; recrystallized from ethanol, mp 219–220 °C (dec.), **IR (ATR, cm** ^**–1**^ **),** 3164 (C–H_stretch_, aromatic), 2973 (C–H_stretch_, aliphatic), 1770 (C=O_stretch_, lactam), 1713 (C=O_stretch_, carbazide), 1550, 1495 (C=C_stretch_ and C=N_stretch_), 1373 (C=S_stretch_), 1184 (C–O_stretch_), 1036, 1011, 971, 838, 790, 750 (C–H_deformation_), ^**1**^ **H NMR (500 MHz, DMSO-*d***_**6**_ **) δ:** 2.34 (s, 3H, **CH**_**3**_), 2.68 (t, 2H, N–CH_2_–**CH**_**2**_), 3.75 (s, 3H, OCH3), 4.05 (t, 2H, N–**CH**_2_–CH_2_), 6.89 (d, 1H, ArH, J: 8.0), 6.94 (d, 2H, ArH), 7.14 (s, 1H, ArH), 7.18–7.21 (m, 3H, ArH), 9.46 (s, 2H, CS–**NH–**CH_2_ and CO–**NH–**NH–CS), 10.01 (s, 1H, CO–NH–**NH–**CS), ^**13**^ **C NMR (100 MHz, DMSO-*d***_**6**_ **) δ:** 21.5 (CH_3_), 31.7 (CH_2_–**C**H_2_–CO), 38.2 (**C**H_2_–CH_2_–CO), 55.6 (OCH_3_), 109.6, 110.3, 113.7, 122.8, 127.9, 131.3, 132.3, 133.7, 140.5 (Ar–C), 154.4 (**C**=O), 169.9 (CH_2_–**C**O–NH), 181.8 (C=S). **HRMS** calcd for C_19_H_20_N_4_O_4_S [M + H]^+^: *m*/*z*, 401.1284; found, 401.1357.

#### *N*-Benzyl-1-[3-(5-methyl-2-benzoxazolinon-3-yl)­propionyl]­thiosemicarbazide (**4bk′**)

Yield: 66.50%; recrystallized from ethanol, mp 266–267 °C (dec.), water solubility <5 mg/mL. **IR (ATR, cm** ^**–1**^ **),** 3320 (NH_stretch_), 3158 (C–H_stretch_, aromatic), 2936 (C–H_stretch_, aliphatic), 1770 (C=O_stretch_, lactam), 1697 (C=O_stretch_, carbazide), 1556, 1496 (C=C_stretch_ and C=N_stretch_), 1381 (C=S_stretch_), 1233 (C–O_stretch_), 1080, 984, 892, 805, 752 (C–H_deformation_), ^**1**^ **H NMR (500 MHz, DMSO-*d***_**6**_ **) δ:** 2.36 (s, 3H, **CH**_**3**_),2.65 (t, 2H, N–CH_2_–**CH**_**2**_), 4.03 (t, 2H, N–**CH**_2_–CH_2_), 4.72 (d, NH–**CH**_**2**_), 6.93 (d, 1H, ArH, J: 8.0), 7.13 (s, 1H, ArH), 7.19 (d, 1H, ArH, J: 8 Hz), 7.22–7.31 (m, 5H, ArH), 8.44 (s, 1H, CS–**NH–**CH_2_), 9.32 (s, 1H, CO–**NH–**NH–CS), 9.92 (s, 1H, CO–NH–**NH–**CS), ^**13**^ **C NMR (100 MHz, DMSO-*d***_**6**_ **) δ:** 21.5 (CH_3_), 31.7 (CH_2_–**C**H_2_–CO), 38.3 (**C**H_2_–CH_2_–CO), 47.0 (NH–CH_2_), 109.6, 110.3, 122.8, 127.0, 127.4, 127.6, 128.5, 131.3, 133.7, 139.7, 140.5 (Ar–C), 154.4 (**C**=O), 169.8 (CH_2_–**C**O–NH), 182.5 (C=S). **HRMS** calcd for C_19_H_20_N_4_O_3_S [M + H]^+^: *m*/*z*, 385.1334; found, 385.1403.

### Preliminary Aqueous Solubility Assessment

The aqueous solubility of the compound was evaluated using a preliminary screening approach via the visual saturation method. Accurately weighed amounts of the compound (1–5 mg) were individually added to 1 mL of distilled water in separate vials. The samples were incubated at a controlled room temperature of 25 ± 1 °C under continuous agitation for 30 min to facilitate dissolution. The solutions were then visually inspected for the presence of undissolved material. Complete dissolution was defined as the absence of visible particles or turbidity. To ensure reproducibility, the assessment was performed in triplicate (*n* = 3). The compound was completely soluble in water at concentrations up to 4 mg/mL, whereas undissolved material was observed at 5 mg/mL. These findings indicate that the preliminary aqueous solubility of the compound lies between 4 and 5 mg/mL under the tested conditions. Based on pharmacopeial classification systems such as the United States Pharmacopeia, this corresponds to a “slightly soluble” compound (approximately 200–250 mL/g).

### Biological Activity

#### In Vitro ChE Enzyme Inhibition Assay

Based on the adapted Ellman method described in our previous research, the inhibitory effects of the synthesized compounds on AChE and BChE enzymes were assessed.^35,48–51^ The enzymes employed in this study were human acetylcholinesterase (CAS No. 9000-81-1) and human butyrylcholinesterase (CAS No. 9001-08-5).

Briefly, for the inhibition assay, each well contained 140 μL of phosphate buffer (pH 8.0), 20 μL of enzyme solution (2.5 U/mL), 20 μL of inhibitor solution, 20 μL of DTNB (0.01 M), and 10 μL of ATC or BTC (0.075 M), reaching a final volume of 210 μL. The enzymes utilized in the experiment were human AChE (CAS No. 9000-81-1) and human BChE (CAS No. 9001-08-5). The first test solution, containing phosphate buffer, enzyme, and DTNB, and the second test solution, containing phosphate buffer and substrate, were prepared in sufficient quantities for the entire plate. Inhibitor solutions at various concentrations were dispensed into the wells using a BioTek Precision XS robotic system and tested in quadruplicate. After 5 min of mixing, the plates were incubated at 25 °C for 15 min, followed by the automated addition of the second test solution. Absorbance was measured at 412 nm before and after a 5 min reaction period using a BioTek Synergy H1 microplate reader.

#### In Vitro MAO Enzyme Inhibition Assay

The MAO inhibition assays were performed using a fluorometric method, previously detailed in our earlier research,^49–55^ and were utilized to assess the inhibitory activity of the synthesized compounds on MAO-A and MAO-B. The assays were conducted using recombinant forms of the human MAO-A and MAO-B enzymes.

Briefly, stock solutions of the test and reference compounds were prepared in DMSO and further diluted so that the final concentration of DMSO in each well was 2%. Recombinant hMAO-A and hMAO-B enzymes were prepared in 0.1 M phosphate buffer (pH 7.4). The working solution consisted of horseradish peroxidase (200 U/mL, 100 μL), Ampliflu Red (20 mM, 200 μL), and tyramine (100 mM, 200 μL), diluted to 10 mL with phosphate buffer. In each well, 20 μL of inhibitor solution and 100 μL of enzyme solution were incubated at 37 °C for 30 min. Subsequently, 100 μL of the working solution was added to initiate the reaction, and fluorescence was recorded every 5 min for 30 min at Ex/Em = 535/587 nm. Control wells contained 2% DMSO instead of the inhibitor solution, and a parallel control using 20 mM H_2_O_2_ was conducted to assess any interference with horseradish peroxidase activity.

The screening data acquired from both enzymatic assays were quantitatively expressed as the mean ± standard deviation (SD). Furthermore, the determination of IC_50_ metrics was executed utilizing *GraphPad Prism* software (version 5.0), employing nonlinear regression to fit a dose–response curve generated by plotting the percent enzyme inhibition against the logarithmic values of test concentrations.

#### Antioxidant Assays

##### Chemicals

Gallic acid, 1,1-diphenyl-2-picrylhydrazyl (DPPH), 2,9-dimethyl-1,10-phenanthroline (neocuproine), 6-hydroxy-2,5,7,8-tetramethylchroman-2-carboxylic acid (Trolox), and potassium persulfate were purchased from Sigma, Germany. 2,2′-Azinobis (3-ethylbenzothiazolin-6-sulfonic acid) (ABTS) was purchased from Cayman Chemical, USA.

##### DPPH (1,1-Diphenyl-2-picrylhydrazyl) Radical Scavenging Activity

The previously described method was applied to test DPPH (1,1-diphenyl-2-picrylhydrazyl) radical scavenging activity.^56^ 50 μL of 1 mM DPPH radical solution prepared in ethanol was added to 150 μL of the compounds and the positive control. The reaction mixtures were incubated for 30 min in the dark and the absorbance values were measured at 517 nm. The assay was carried out in duplicate. The radical scavenging capacity was calculated using the following formula:

$$Inhibition\%=\left[\left(A_{control}-A_{sample}\right)/A_{control}\right]\times 100$$

where *A*_control_ is the absorbance of the negative control and *A*_sample_ is the absorbance of the sample.

##### ABTS^+^ (2,2′-Azino-bis­(3- ethylbenzothiazoline-6-sulfonic acid) Radical Scavenging Activity

ABTS radical cation scavenging activity assay was performed according to the previously described method^37^ with slight modifications.^57^ ABTS radical cation (ABTS^•+^) was generated by reacting 7 mM ABTS solution with 2.45 mM potassium persulfate for 12–16 h. ABTS^•+^ solution was diluted to an absorbance of 0.700 ± 0.02 at 734 nm before use. 100 μg/mL concentration solutions of the compounds were mixed with that solution. The absorbance was measured at 734 nm after 6 min of incubation at room temperature in the dark. The assay was performed in duplicate. Trolox was used as a positive control. The results were expressed as the Trolox equivalents (mg TE/g compound).

##### Cupric Ion Reducing Antioxidant Capacity (CUPRAC) Assay

The assay was applied according to the method by Apak et al.^58^ with slight modifications.^57^ First, 50 μL of 0.01 M copper­(II) chloride solution, 50 μL of 7.5 mM neocuproine solution, and 50 μL of 1 M ammonium acetate buffer (pH 7.0) were mixed. Twenty-five μL of compounds or positive control and 25 μL of distilled water were added to the initial mixture. The reaction mixtures were incubated for 30 min in the dark and the absorbance values were measured at 450 nm. The assay was performed in duplicate. Gallic acid was used as a positive control. Results were represented as gallic acid equivalents (mg GAE/g compound).

#### Cytotoxic Activity

##### Preparation of the Stock Solutions of Pure Compounds for the Determination of Cytotoxicity

The compounds were weighed and dissolved in DMSO to obtain 20 mM stock solutions, from which solutions of different concentrations could be prepared.

##### Determination of Cytotoxic Activity by the Resazurin Reduction Assay

H9c2 and BV-2 healthy cell lines were used in the present work. Their origins and sustainment were previously reported (ATCC, 2025). Experiments were conducted according to the literature.^59^

The graphs of cell survival % of the compounds were created by using Microsoft Excel.

### In Silico Studies

#### Prediction of ADME Parameters

To project the pharmacokinetic behaviors of the newly synthesized derivatives, an *in silico* profiling protocol was deployed, wherein their fundamental physicochemical descriptors were computed utilizing the *QikProp* software.^60^

#### Molecular Docking Studies

To decipher the precise molecular architectures and noncovalent contact points of the synthesized hybrids within the catalytic pockets of AChE and MAO-B, a structure-guided *in silico* docking protocol was enacted. Computational binding assessments were conducted utilizing the high-resolution crystal coordinates of human AChE (PDB ID: 4EY7)^41^ and MAO-B (PDB ID: 2V5Z).^61^ The parameters and workflow applied for these molecular simulation setups were strictly aligned with the validated methodologies documented in our earlier reports.^48–52^

#### Molecular Dynamic Simulations Studies

To rigorously evaluate the dynamic behavior and conformational stability of the most promising *in vitro* hits in light of their initial docking configurations, 100 ns MDSs were executed utilizing the Maestro Desmond interface package.^62^ The comprehensive workflow encompassing the solvated system preparation, the production run of the MDS, and the postsimulation trajectory data retrieval was carefully carried out in strict accordance with the established protocols detailed in our earlier literature.^51,54^

## Supplementary Material



## Data Availability

The data that supports the findings of this study are available in the Supporting Information of this article.

## Notes

^1^H NMR, ^13^C NMR, and HRMS spectra of all synthesized compounds (**4aa′–4bk′**); MDS results for the **4ac′**-AChE, **4bk′**-AChE, **4ac′**-MAO-B, and **4bk′**-MAO-B complexes; and tables of interacting amino acid residues and interaction fractions from MDSs (PDF)

## ⊥

Department of Pharmaceutical Chemistry, Faculty of Pharmacy, EBYU, Erzincan 24100, Türkiye
