# Supplementary material for: Benzoxazolinone-Based Propionyl Thiosemicarbazides as Multi-Target-Directed Ligands for Alzheimer’s Disease: Cholinesterase and MAO Inhibition, Docking, and Molecular Dynamics
Source: ACS Omega. 2026 Jul 13;11(29):43129–56. doi: 10.1021/acsomega.5c12925 (PMC13425522; doi:10.1021/acsomega.5c12925)
Supplement: Supplementary file 1 [file ao5c12925_si_001.pdf]

SUPPORTING INFORMATION

Benzoxazolinone Based Propionyl Thiosemicarbazides as Multi-Target-Directed Ligands for Alzheimer's Disease: Cholinesterase and MAO Inhibition, Docking and Molecular Dynamics

Hayrünnisa Taşci,[a*,b] Ahmet Avcı,[b] Nadire Özenver,[c] Begüm Nurpelin Sağlık Özkan,[d]
Birsen Tozkoparan[b] and Nesrin Gökhan Kelekçi [**,b]

[a]Department of Pharmaceutical Chemistry, Faculty of Pharmacy, EBYU, Erzincan, 24100, Türkiye.
[b]Department of Pharmaceutical Chemistry, Faculty of Pharmacy, Hacettepe University, Ankara, 06100, Türkiye.
[c]Department of Pharmacognosy, Faculty of Pharmacy, Hacettepe University, Ankara, 06100, Türkiye.
[d]Department of Pharmaceutical Chemistry, Faculty of Pharmacy, Anadolu University, Eskişehir, 26470, Türkiye.

* Current address

** Corresponding author. Tel.: +903123053017; fax:+903123114777; e-mail: onesrin@hacettepe.edu.tr

Contents		Page
**Figure S1**	1H-NMR spectrum of compound 4aa'	S5
**Figure S2**	1H-NMR spectrum of compound 4ab'	S6
**Figure S3**	1H-NMR spectrum of compound 4ac'	S7
**Figure S4**	1H-NMR spectrum of compound 4ad'	S8
**Figure S5**	1H-NMR spectrum of compound 4ae'	S9
**Figure S6**	1H-NMR spectrum of compound 4af'	S10
**Figure S7**	1H-NMR spectrum of compound 4ag'	S11
**Figure S8**	1H-NMR spectrum of compound 4ah'	S12
**Figure S9**	1H-NMR spectrum of compound 4ai'	S13
**Figure S10**	1H-NMR spectrum of compound 4aj'	S14
**Figure S11**	1H-NMR spectrum of compound 4ak'	S15
**Figure S12**	1H-NMR spectrum of compound 4ba'	S16
**Figure S13**	1H-NMR spectrum of compound 4bb'	S17
**Figure S14**	1H-NMR spectrum of compound 4bc'	S18
**Figure S15**	1H-NMR spectrum of compound 4bd'	S19
**Figure S16**	1H-NMR spectrum of compound 4be'	S20
**Figure S17**	1H-NMR spectrum of compound 4bf'	S21
**Figure S18**	1H-NMR spectrum of compound 4bg'	S22
**Figure S19**	1H-NMR spectrum of compound 4bh'	S23
**Figure S20**	1H-NMR spectrum of compound 4bi'	S24
**Figure S21**	1H-NMR spectrum of compound 4bj'	S25
**Figure S22**	1H-NMR spectrum of compound 4bk'	S26
**Figure S23**	13C-NMR spectrum of compound 4aa'	S28
**Figure S24**	13C-NMR spectrum of compound 4ab'	S29
**Figure S25**	13C-NMR spectrum of compound 4ac'	S30
**Figure S26**	13C-NMR spectrum of compound 4ad'	S31
**Figure S27**	13C-NMR spectrum of compound 4ae'	S32
**Figure S28**	13C-NMR spectrum of compound 4af'	S33
**Figure S29**	13C-NMR spectrum of compound 4ag'	S34
**Figure S30**	13C-NMR spectrum of compound 4ah'	S35
**Figure S31**	13C-NMR spectrum of compound 4ai'	S36
**Figure S32**	13C-NMR spectrum of compound 4aj'	S37
**Figure S33**	13C-NMR spectrum of compound 4ak'	S38
**Figure S34**	13C-NMR spectrum of compound 4ba'	S39
**Figure S35**	13C-NMR spectrum of compound 4bb'	S40
**Figure S36**	13C-NMR spectrum of compound 4bc'	S41
**Figure S37**	13C-NMR spectrum of compound 4bd'	S42
**Figure S38**	13C-NMR spectrum of compound 4be'	S43
**Figure S39**	13C-NMR spectrum of compound 4bf'	S44
**Figure S40**	13C-NMR spectrum of compound 4bg'	S45
**Figure S41**	13C-NMR spectrum of compound 4bh'	S46
**Figure S42**	13C-NMR spectrum of compound 4bi'	S47
**Figure S43**	13C-NMR spectrum of compound 4bj'	S48
**Figure S44**	13C-NMR spectrum of compound 4bk'	S49

**Figure S45**	HRMS spectrum of compound 4aa'	S50
**Figure S46**	HRMS spectrum of compound 4ab'	S50
**Figure S47**	HRMS spectrum of compound 4ac'	S50
**Figure S48**	HRMS spectrum of compound 4ad'	S51
**Figure S49**	HRMS spectrum of compound 4ae'	S51
**Figure S50**	HRMS spectrum of compound 4af'	S51
**Figure S51**	HRMS spectrum of compound 4ag'	S52
**Figure S52**	HRMS spectrum of compound 4ah'	S52
**Figure S53**	HRMS spectrum of compound 4ai'	S52
**Figure S54**	HRMS spectrum of compound 4aj'	S53
**Figure S55**	HRMS spectrum of compound 4ak'	S53
**Figure S56**	HRMS spectrum of compound 4ba'	S53
**Figure S57**	HRMS spectrum of compound 4bb'	S54
**Figure S58**	HRMS spectrum of compound 4bc'	S54
**Figure S59**	HRMS spectrum of compound 4bd'	S54
**Figure S60**	HRMS spectrum of compound 4be'	S55
**Figure S61**	HRMS spectrum of compound 4bf'	S55
**Figure S62**	HRMS spectrum of compound 4bg'	S55
**Figure S63**	HRMS spectrum of compound 4bh'	S56
**Figure S64**	HRMS spectrum of compound 4bi'	S56
**Figure S65**	HRMS spectrum of compound 4bj'	S56
**Figure S66**	HRMS spectrum of compound 4bk'	S57
**Figure S67**	Molecular dynamic results of compound 4ac'–AChE (PDB ID: 4EY7) complex	S57
**Figure S68**	Molecular dynamic results of compound 4bk'–AChE (PDB ID: 4EY7) complex.	S58
**Figure S69**	Molecular dynamic results of compound 4ac'–MAO-B (PDB ID: 2V5Z) complex.	S58
**Figure S70**	Molecular dynamic results of compound 4bk'–MAO-B (PDB ID: 2V5Z) complex.	S59
**Table S1**	Interacting amino acids and fractions for compounds 4ac' and 4bk' on AChE (PDB ID: 4EY7) enzyme.	S59
**Table S2**	Interacting amino acids and fractions for compounds 4ac' and 4bk' on MAO-B (PDB ID: 2V5Z) enzyme.	S60

^{1}H-NMR spectra of 4aa'-k'/4ba'-k' compounds (S5-S26).

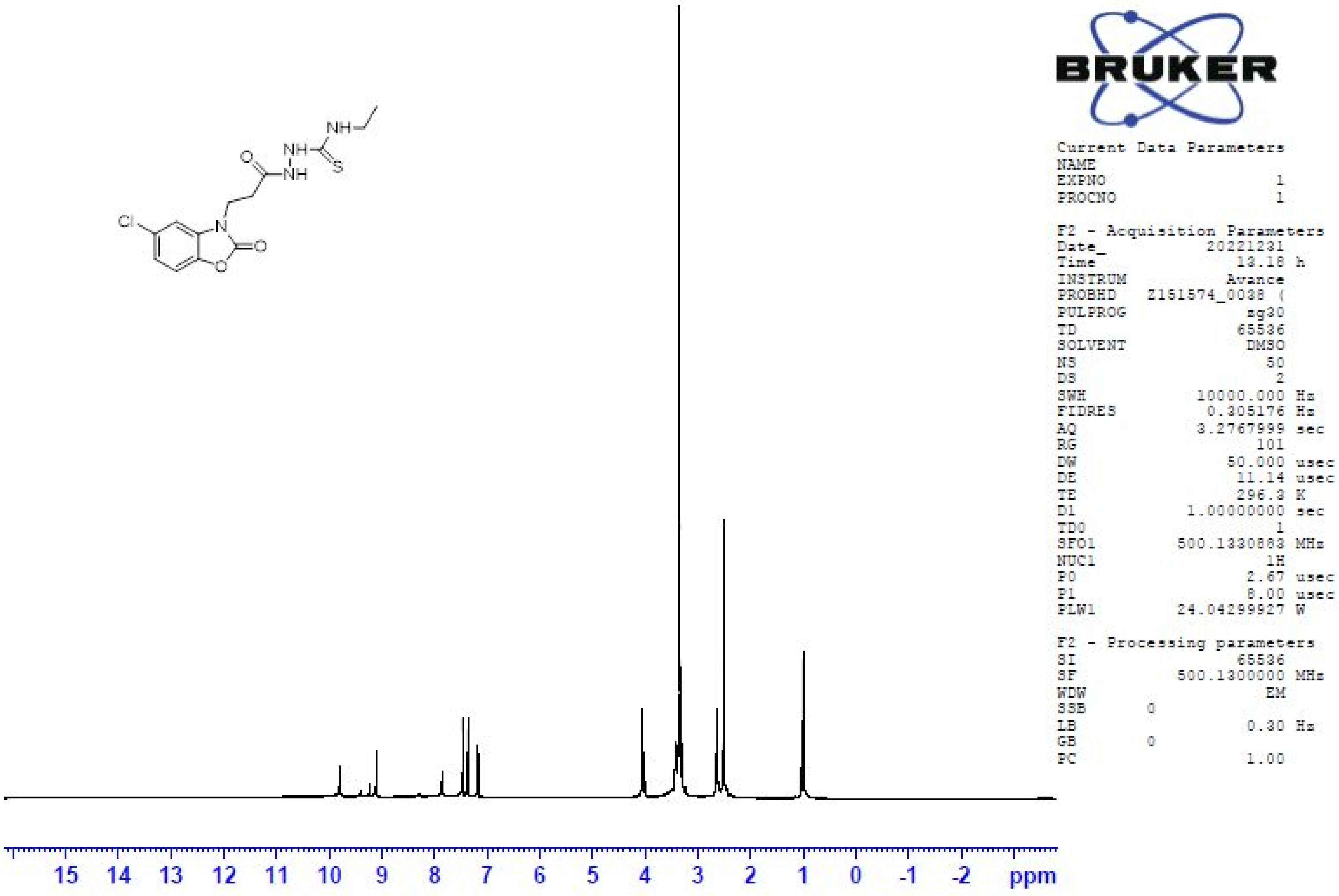

Figure S1. 1H-NMR spectrum of compound 4aa'

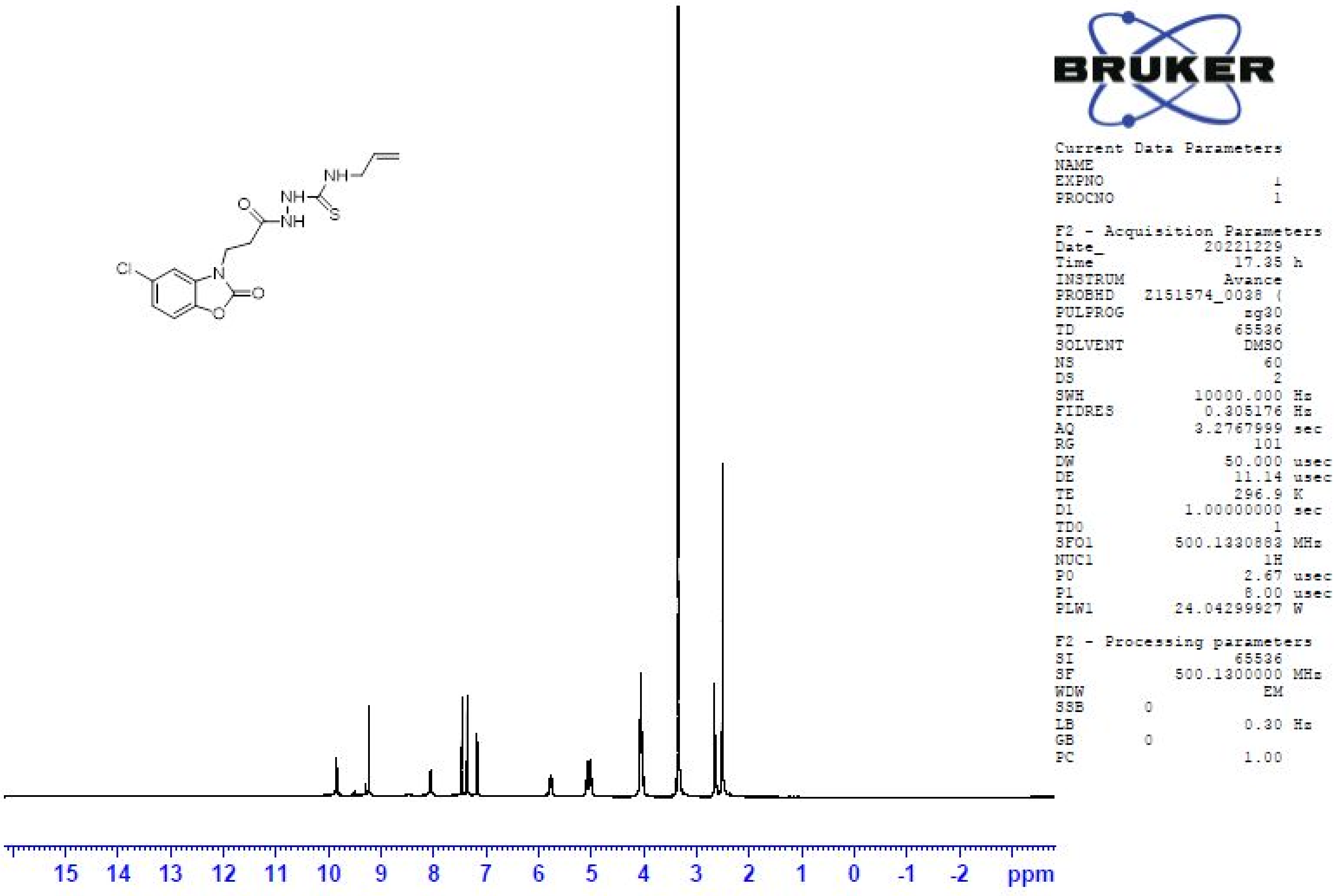

Figure S2. 1H-NMR spectrum of compound 4ab'

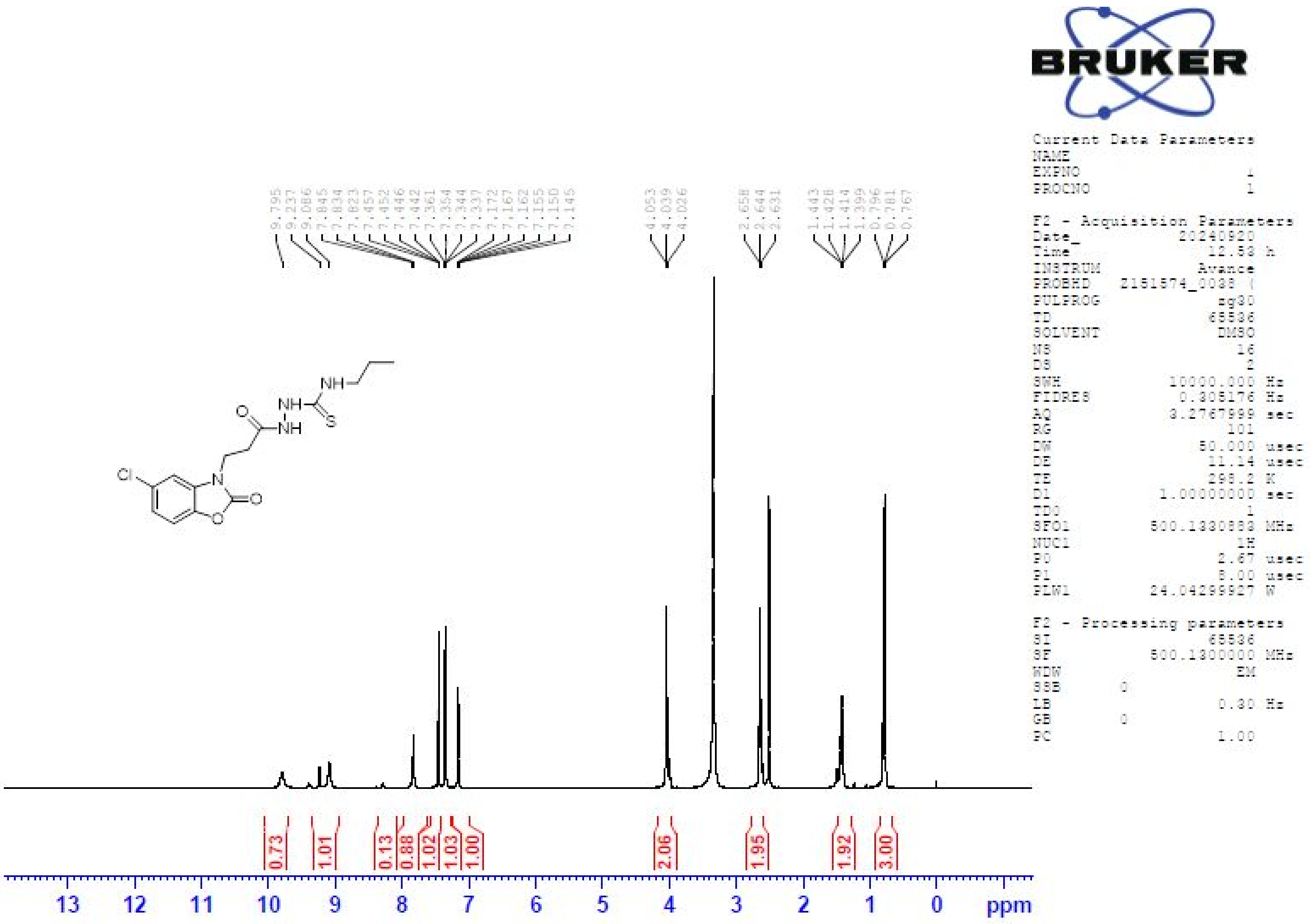

Figure S3. 1H-NMR spectrum of compound 4ac'

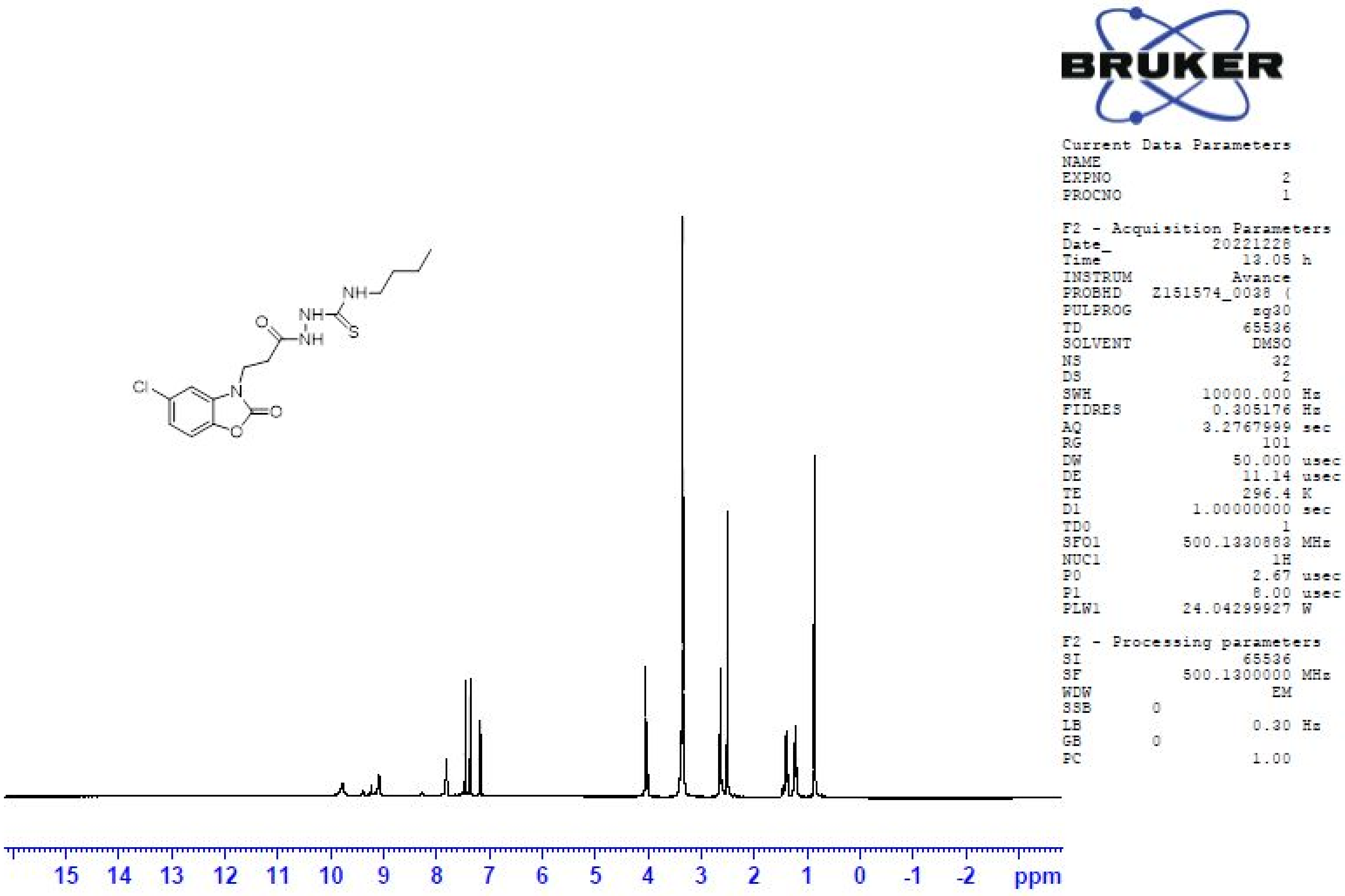

Figure S4. 1H-NMR spectrum of compound 4ad'

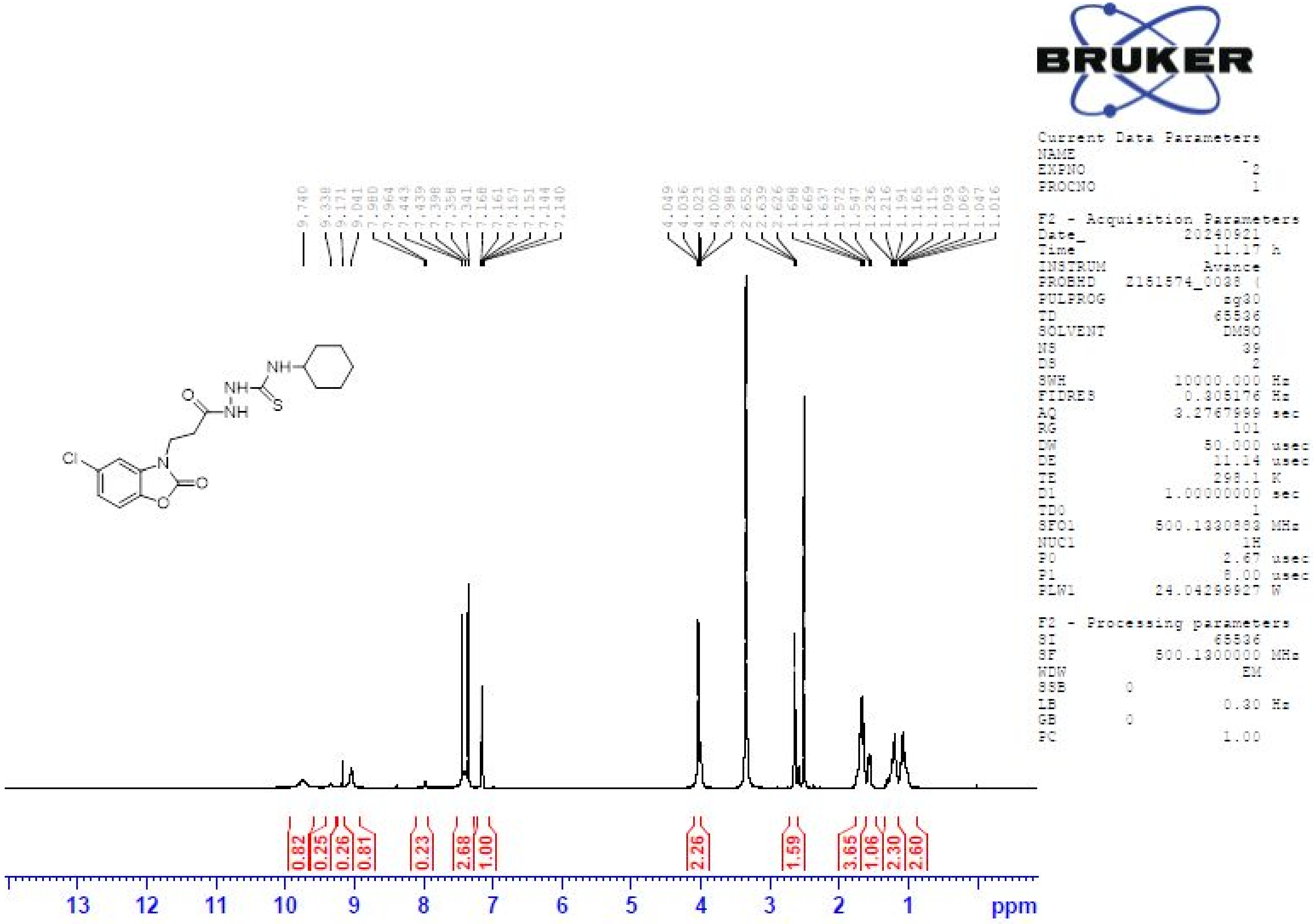

Figure S5. 1H-NMR spectrum of compound 4ae'

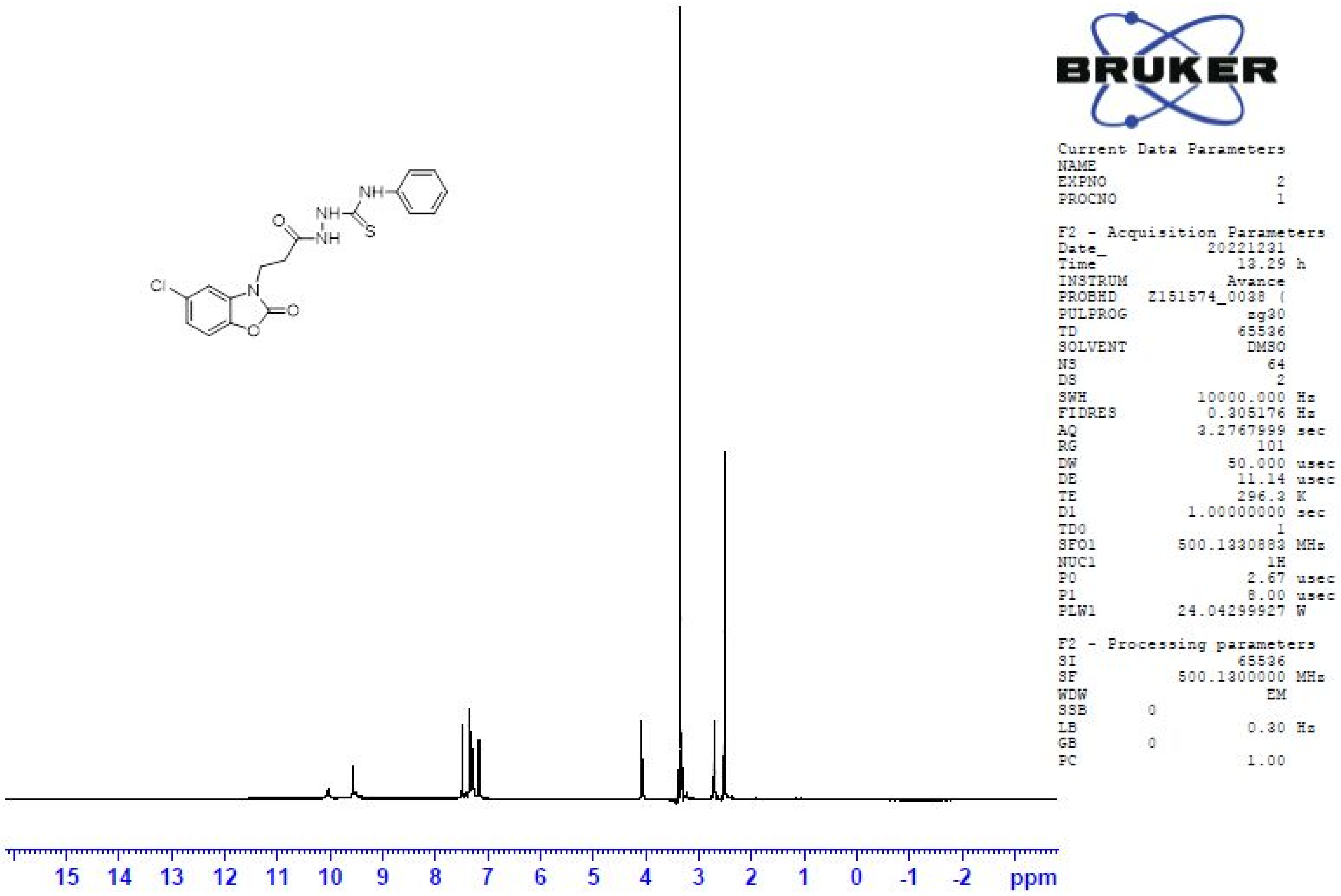

Figure S6. 1H-NMR spectrum of compound 4af'

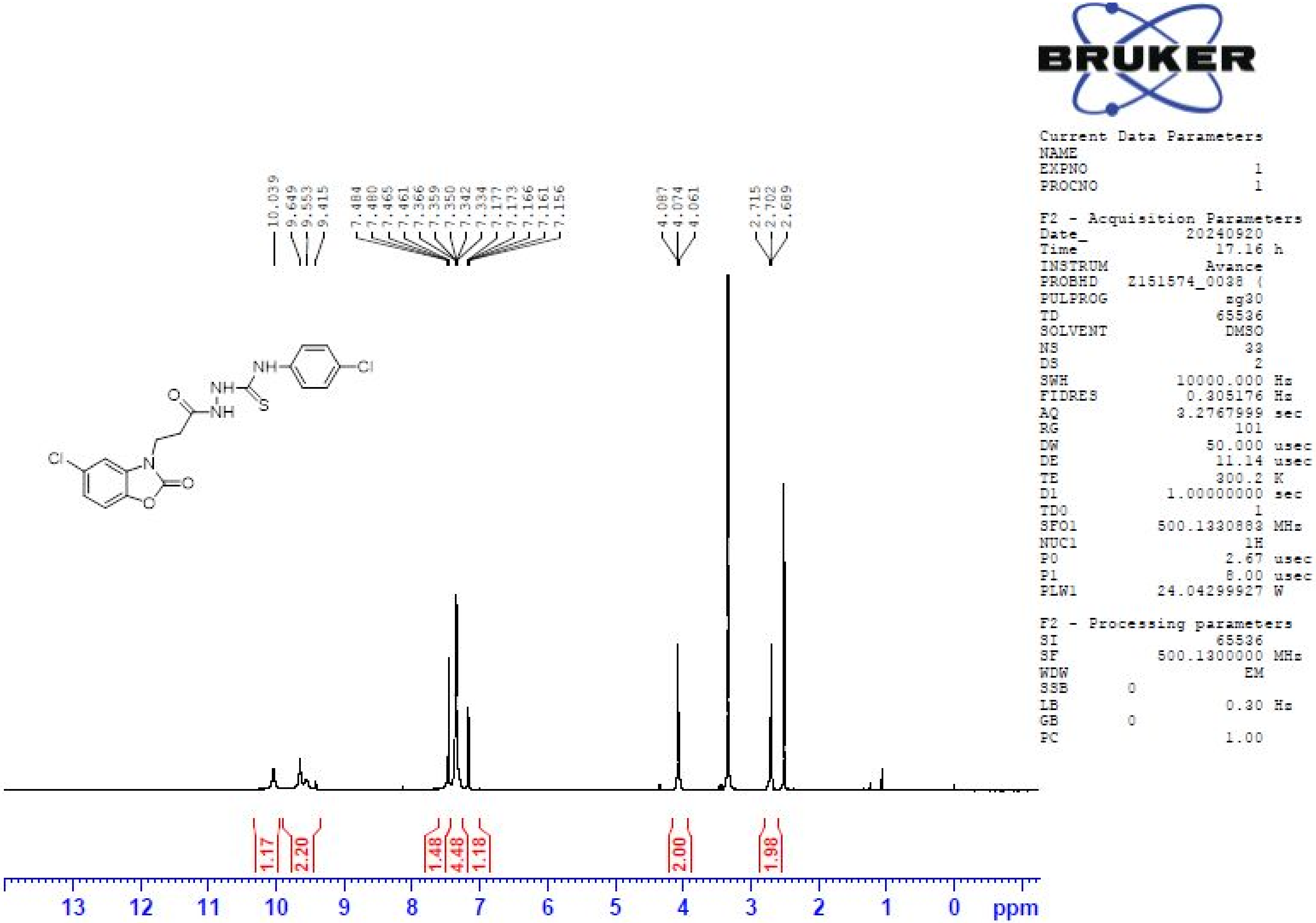

Figure S7. 1H-NMR spectrum of compound 4ag'

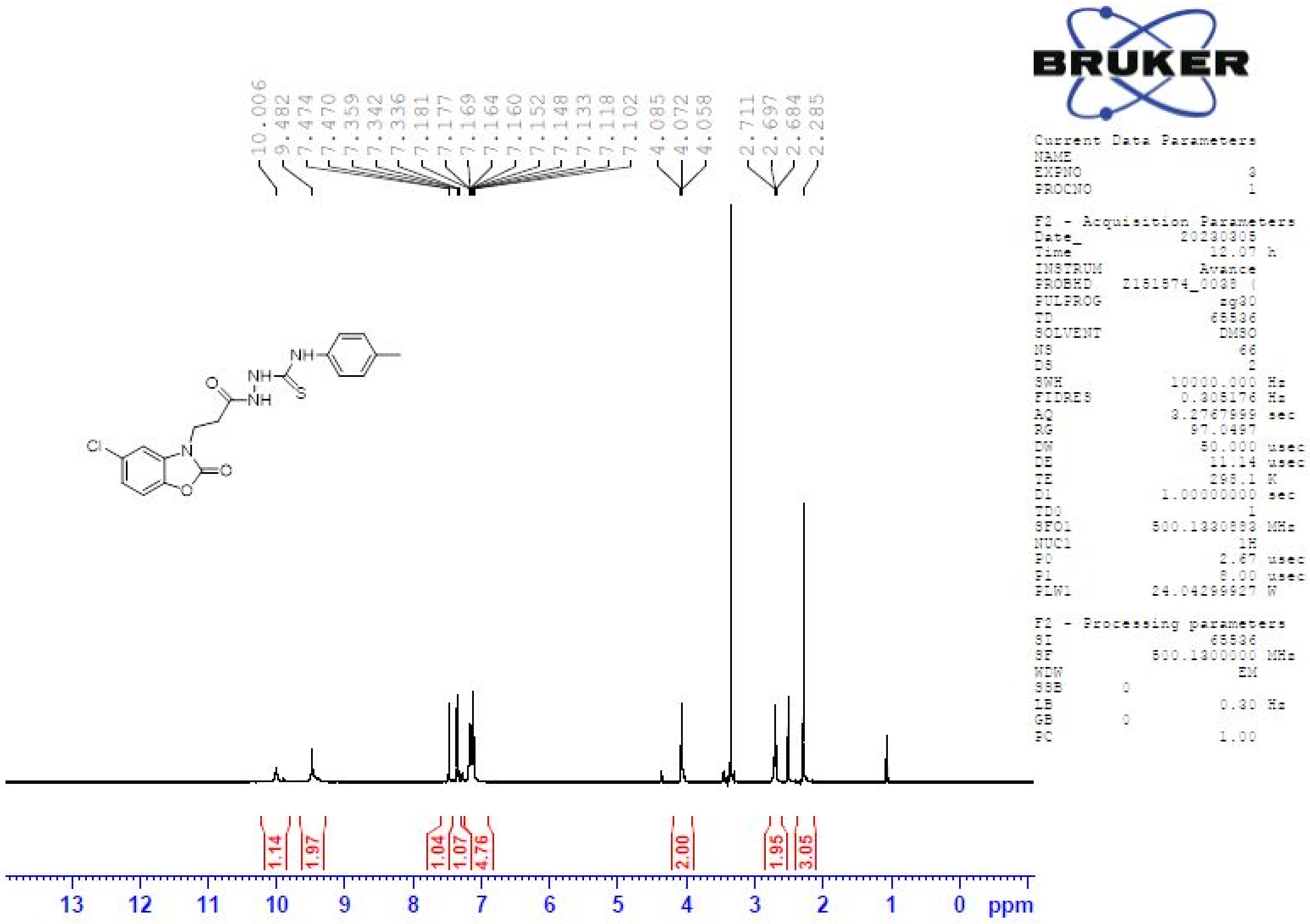

Figure S8. 1H-NMR spectrum of compound 4ah'

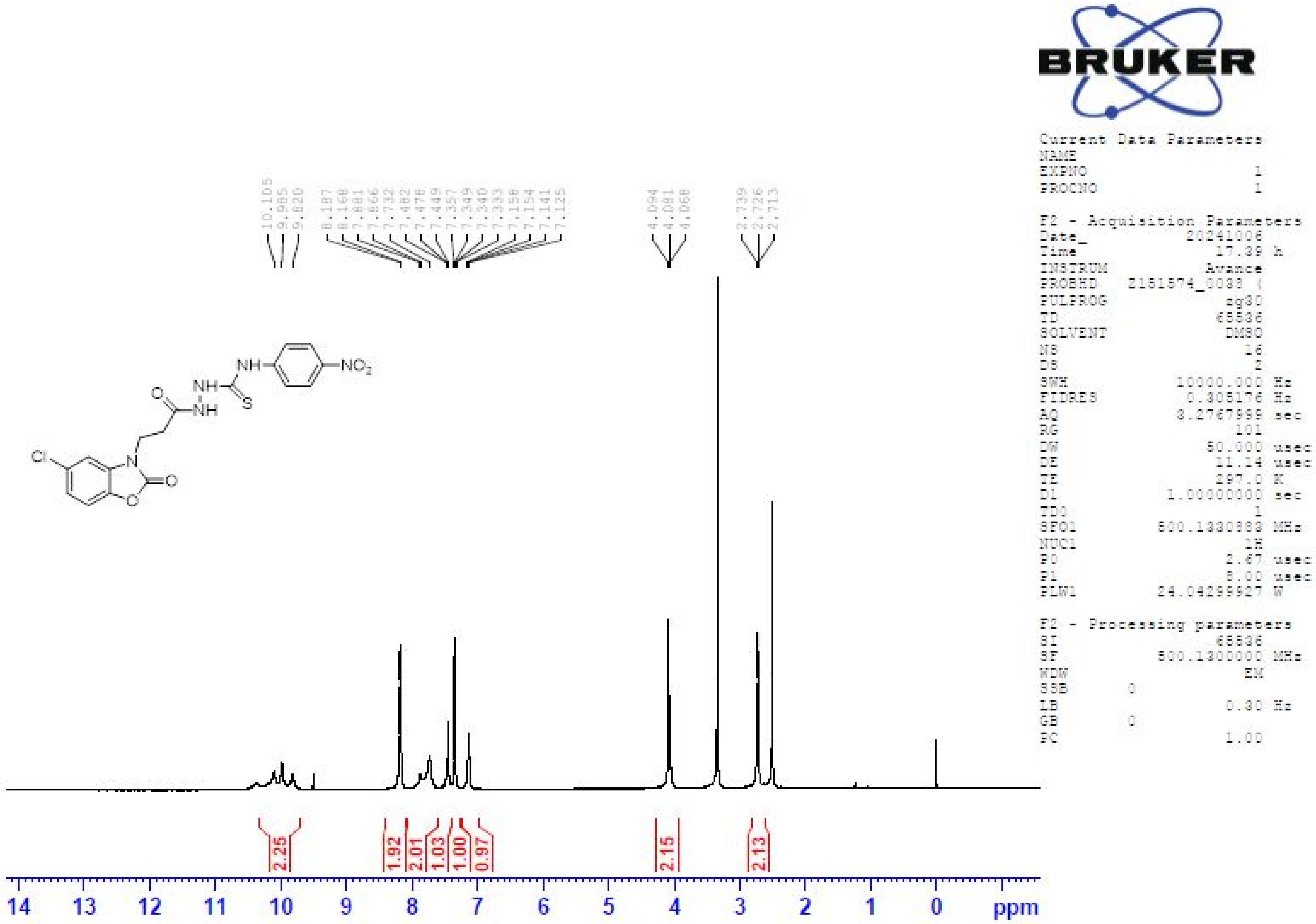

Figure S9. 1H-NMR spectrum of compound 4ai'

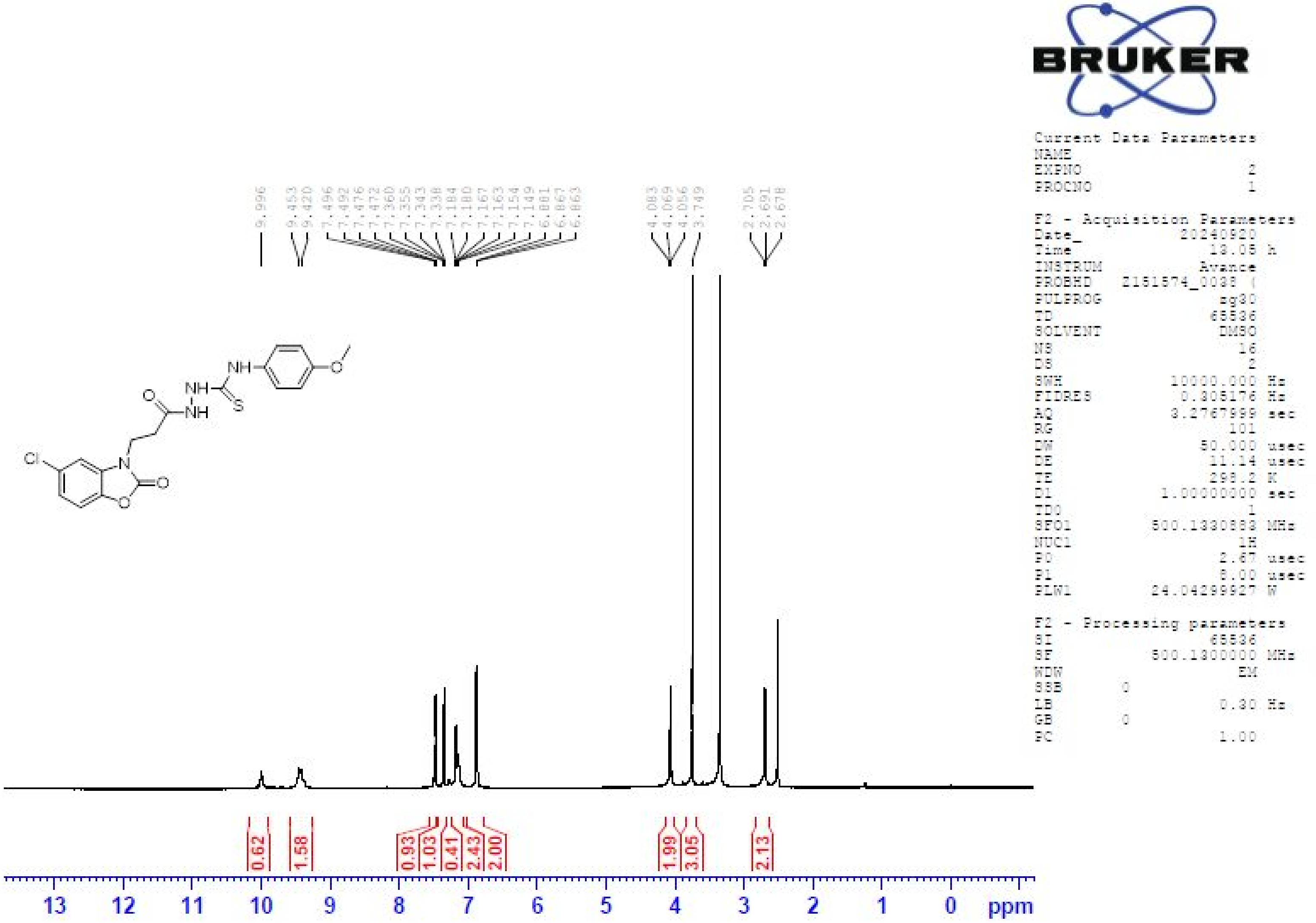

Figure S10. 1H-NMR spectrum of compound 4aj'

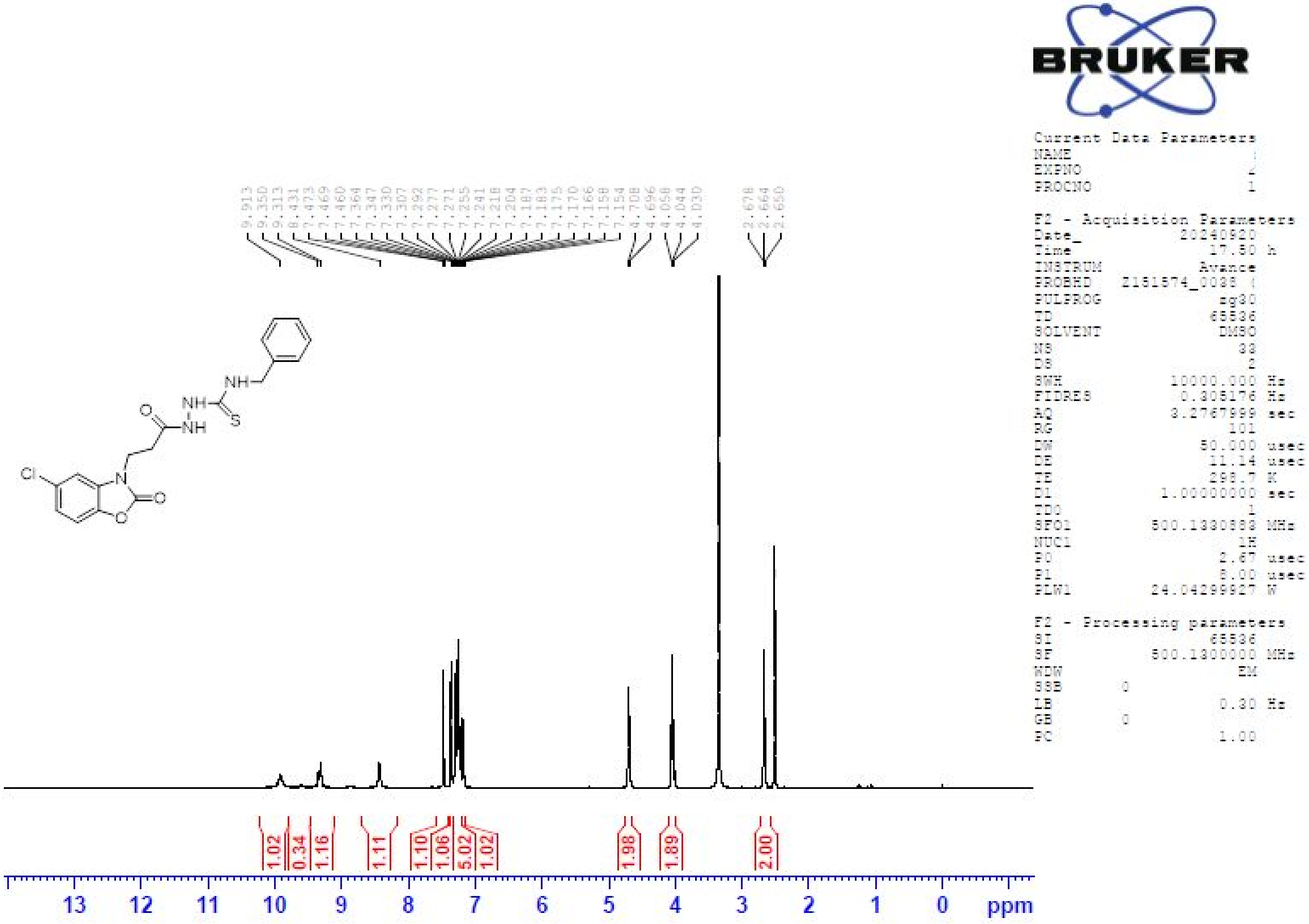

Figure S11. 1H-NMR spectrum of compound 4ak'

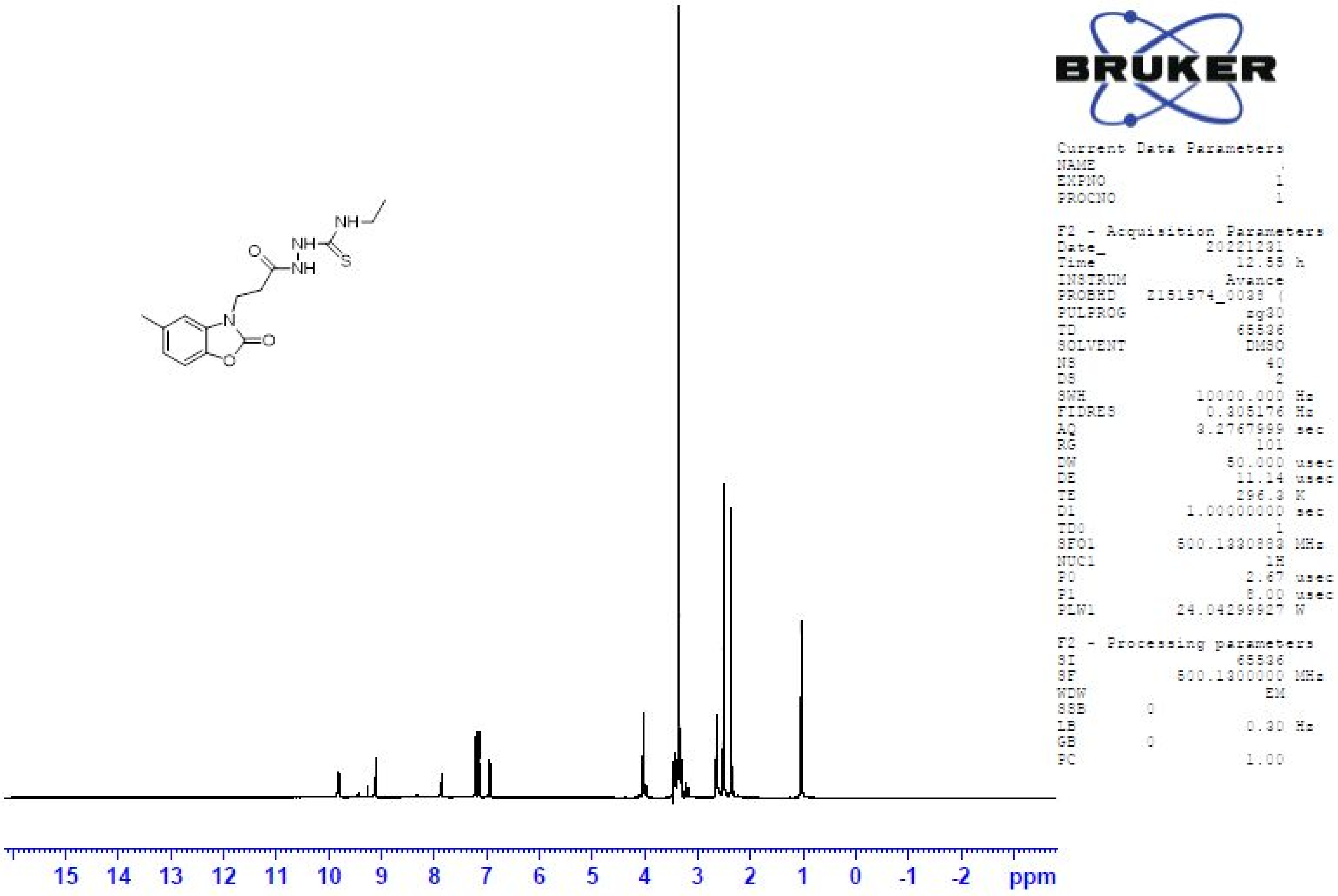

Figure S12. 1H-NMR spectrum of compound 4ba'

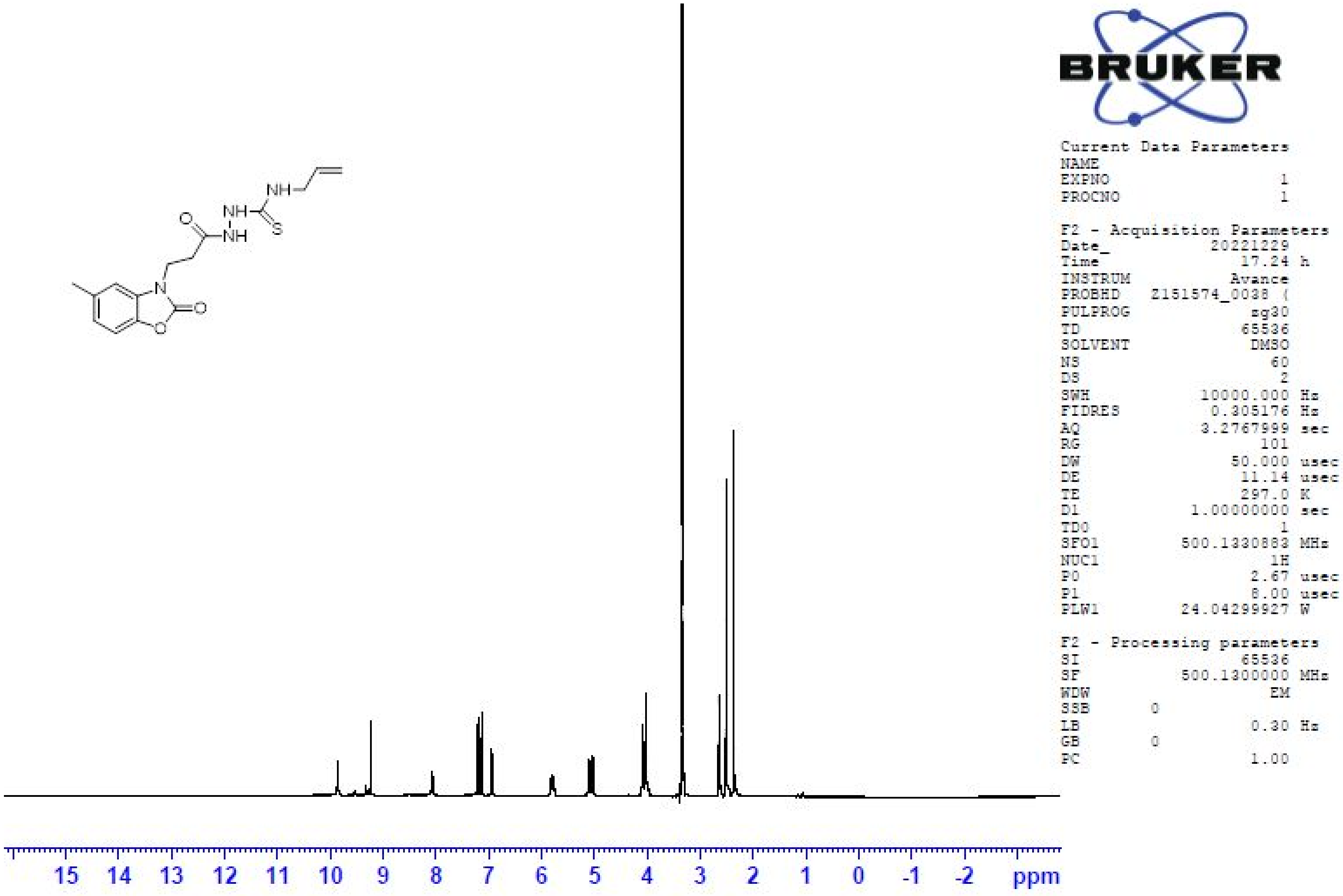

Figure S13. 1H-NMR spectrum of compound 4bb'

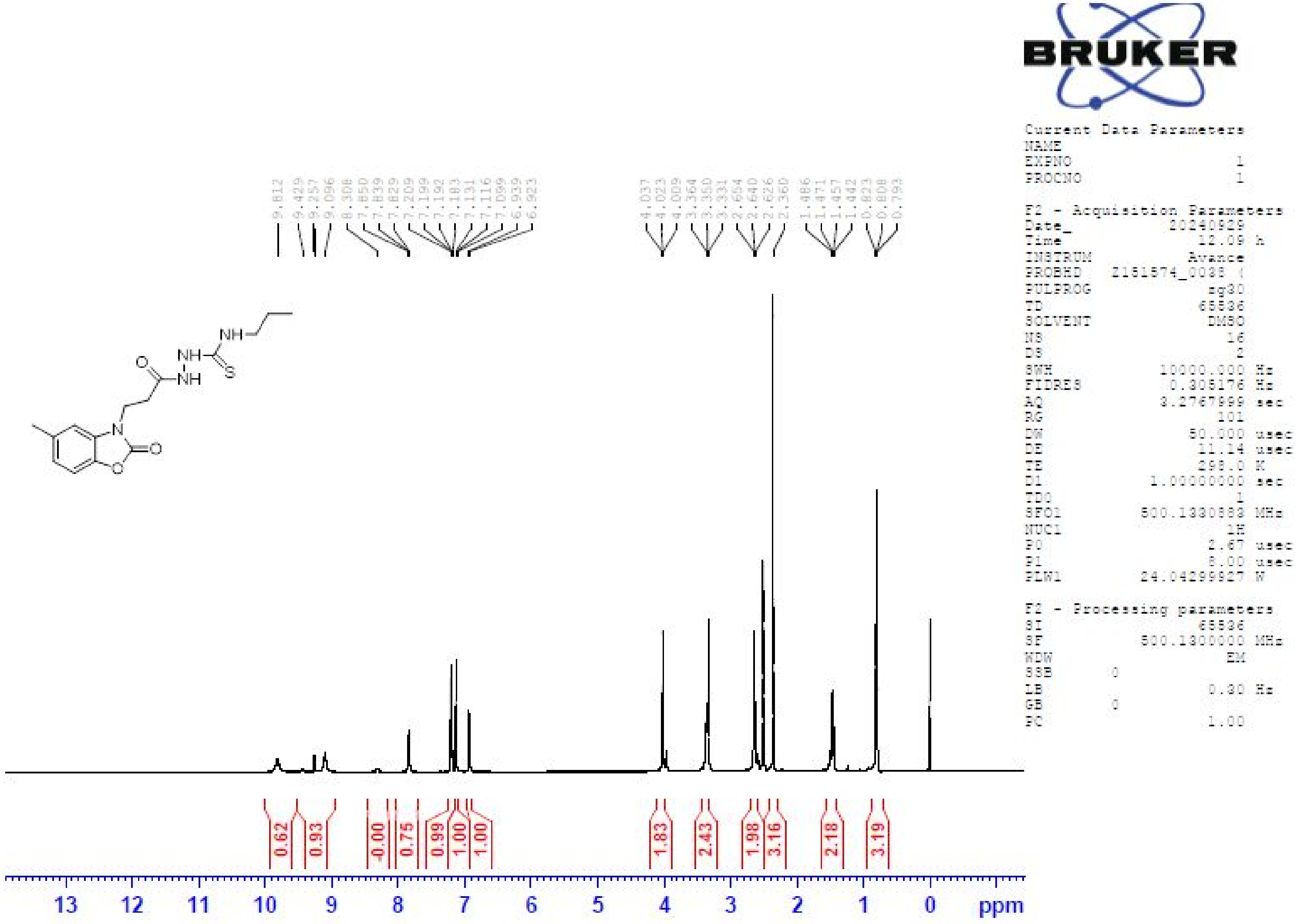

Figure S14. 1H-NMR spectrum of compound 4bc'

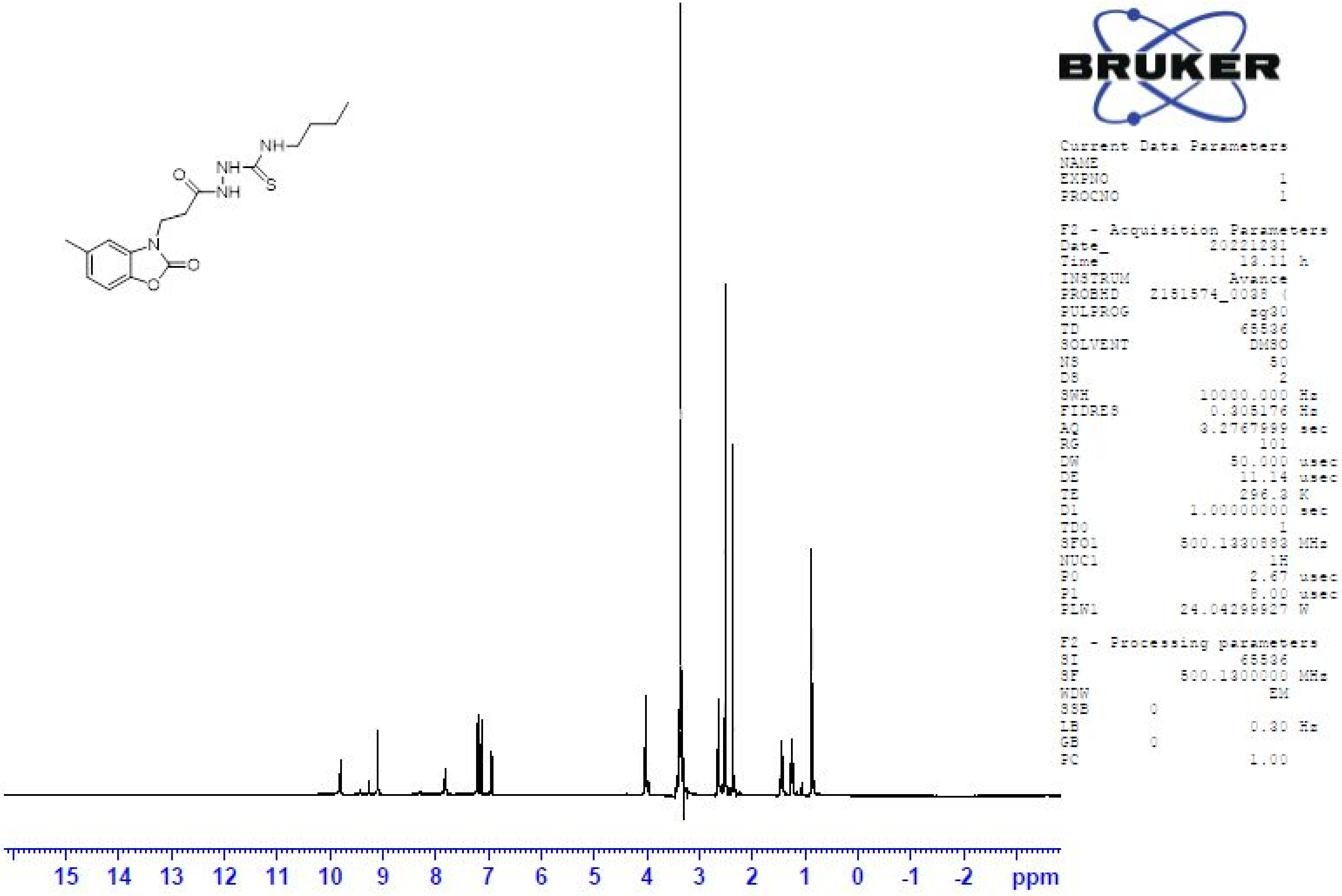

Figure S15. 1H-NMR spectrum of compound 4bd'

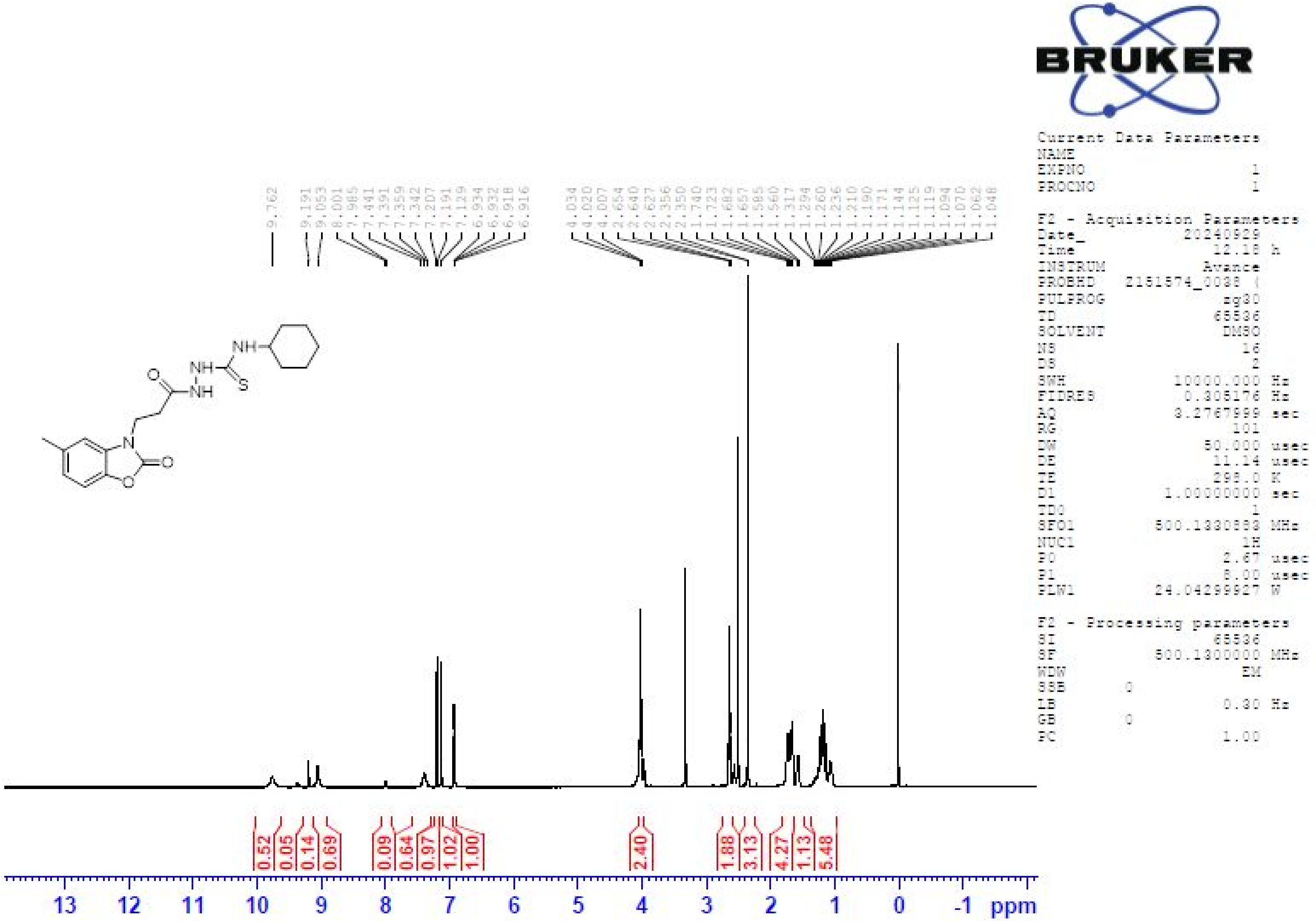

Figure S16. 1H-NMR spectrum of compound 4be'

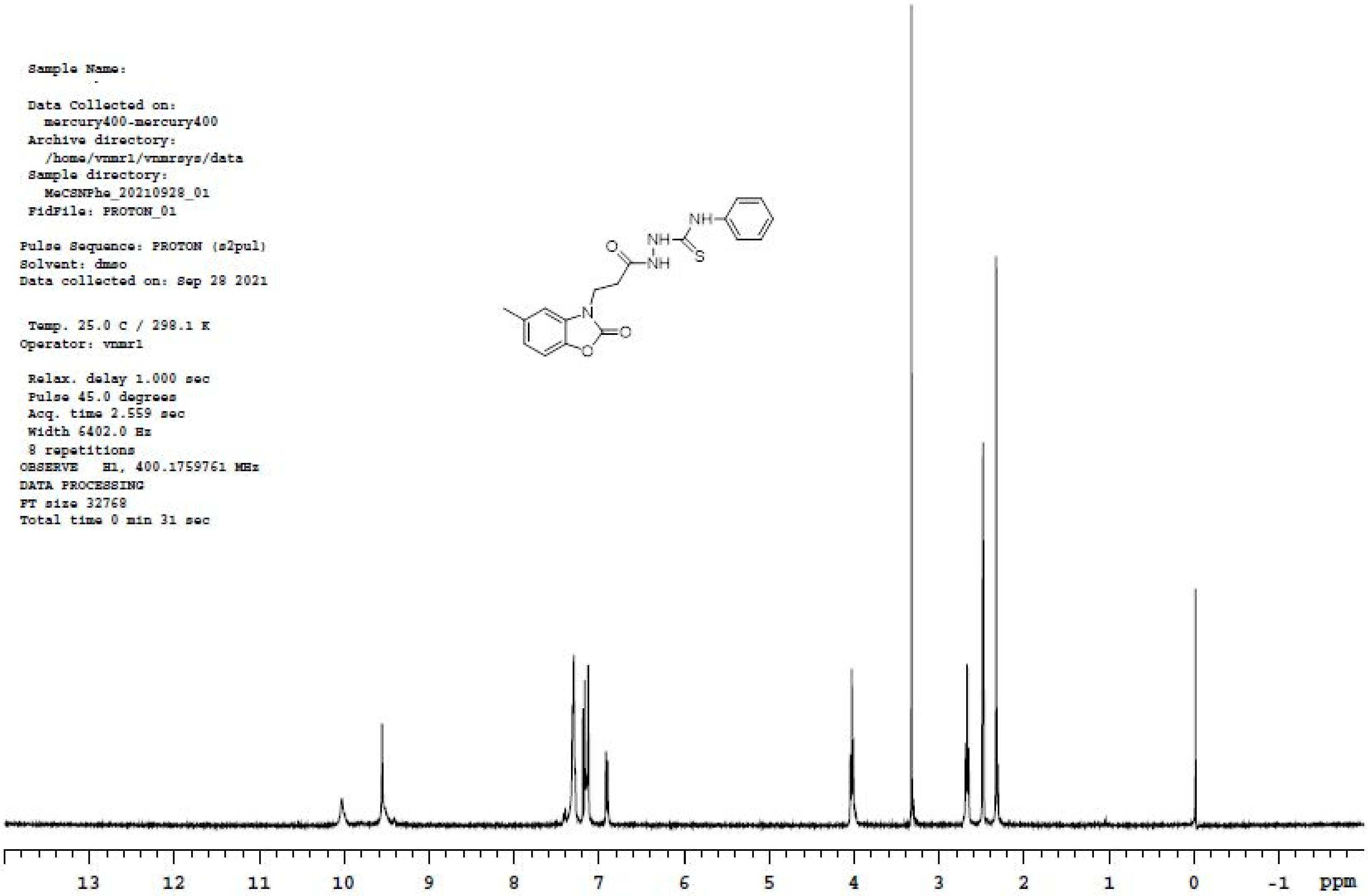

Figure S17. 1H-NMR spectrum of compound 4bf'

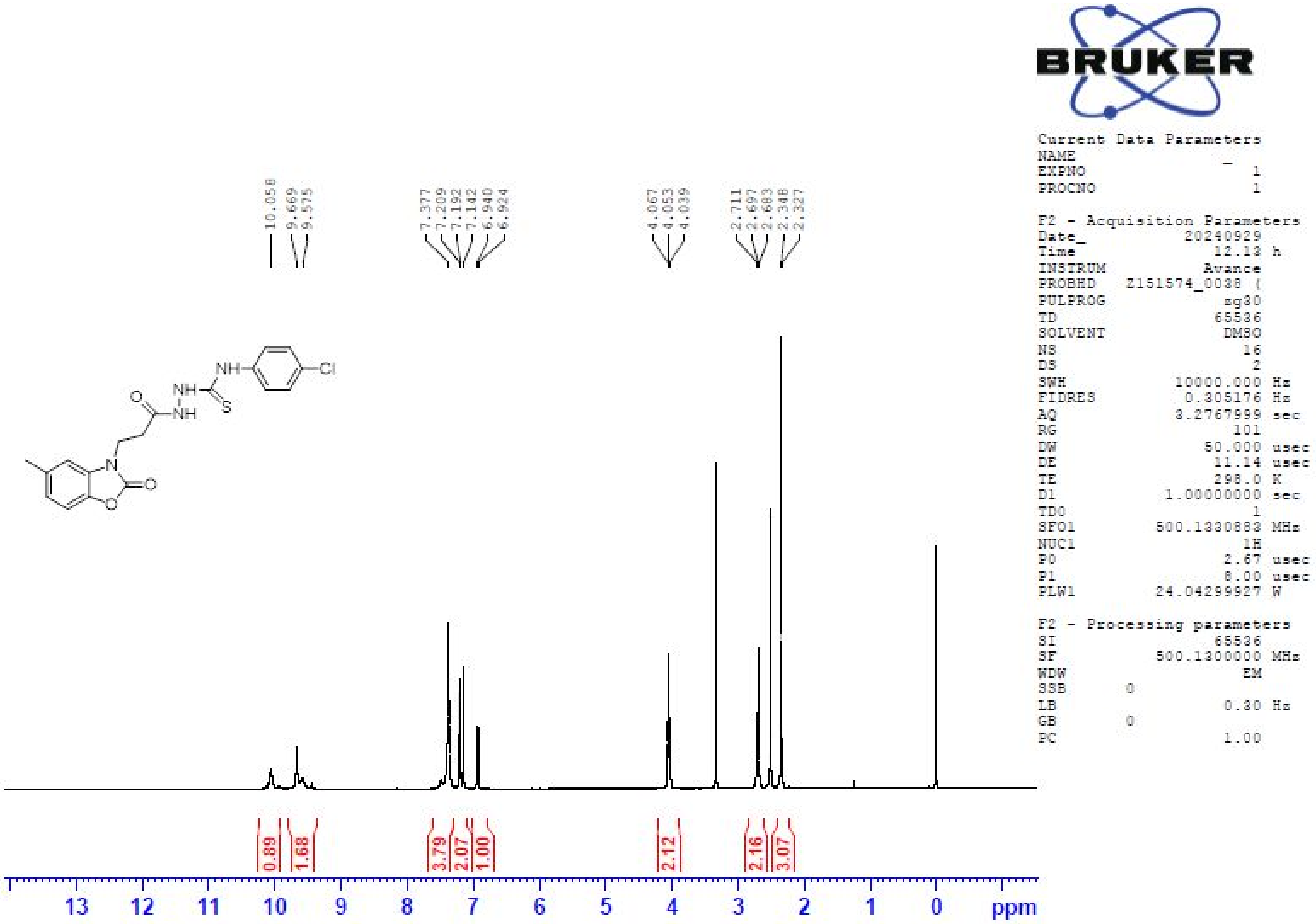

Figure S18. 1H-NMR spectrum of compound 4bg'

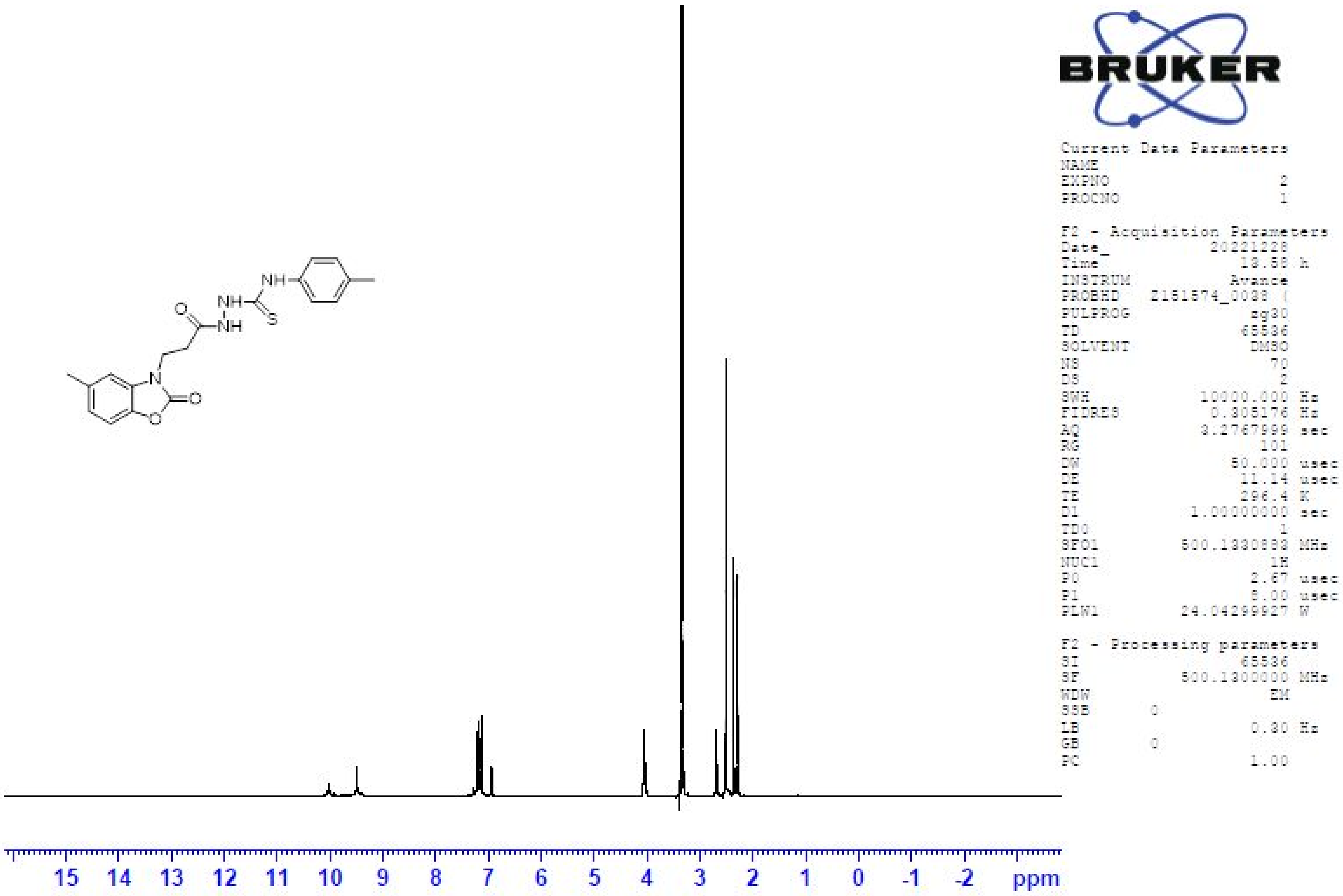

Figure S19. 1H-NMR spectrum of compound 4bh'

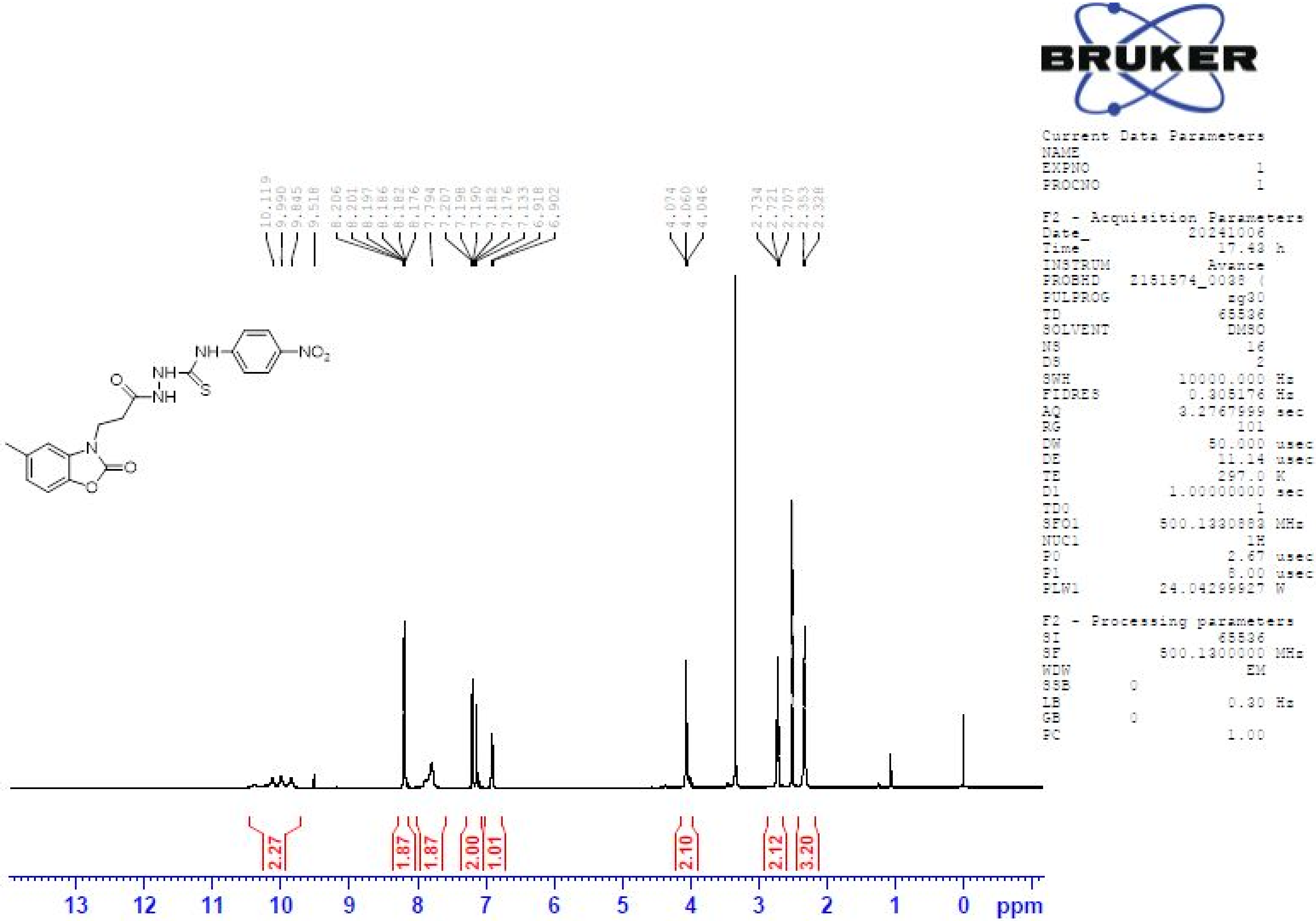

Figure S20. 1H-NMR spectrum of compound 4bi'

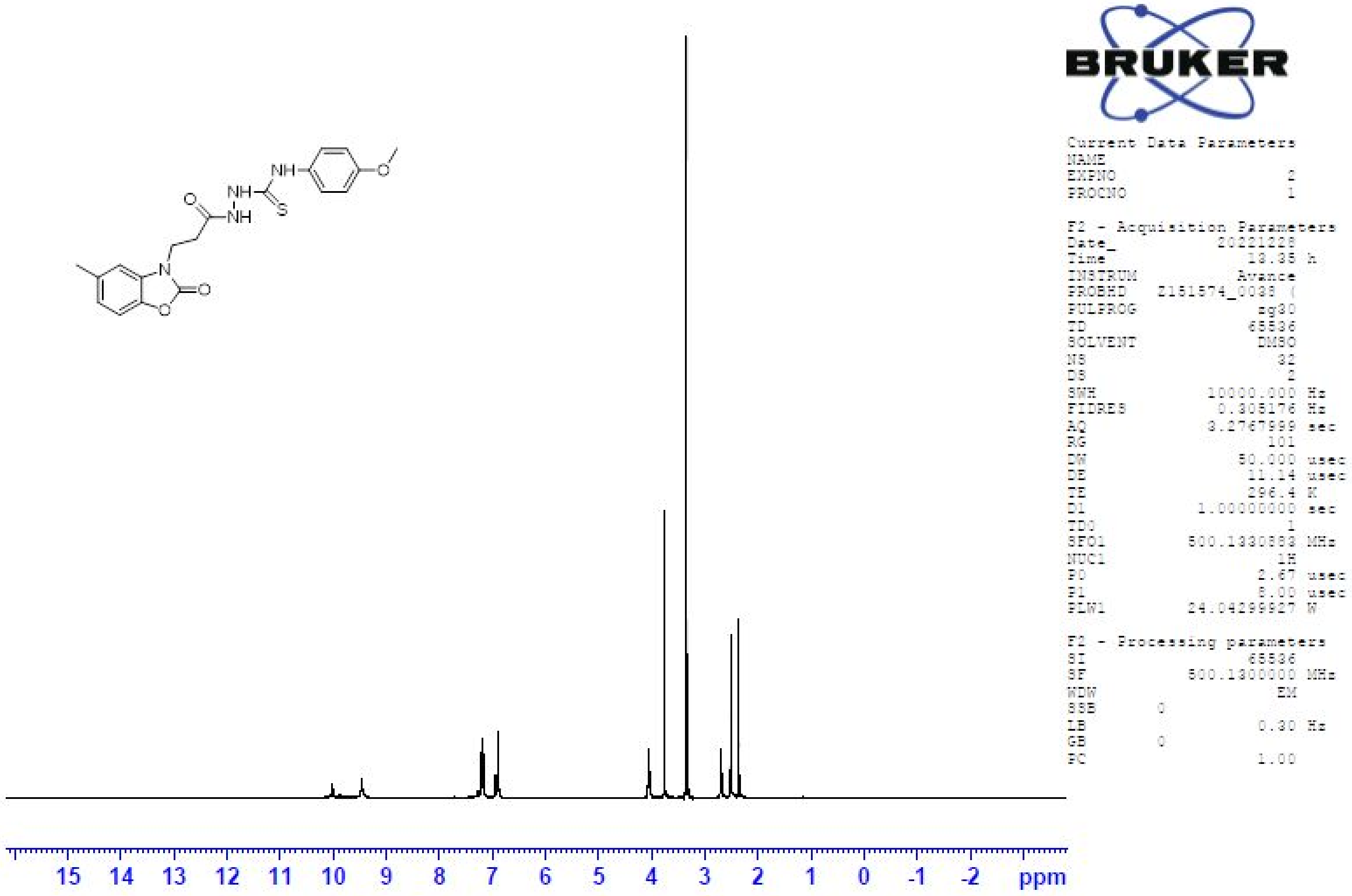

Figure S21. 1H-NMR spectrum of compound 4bj'

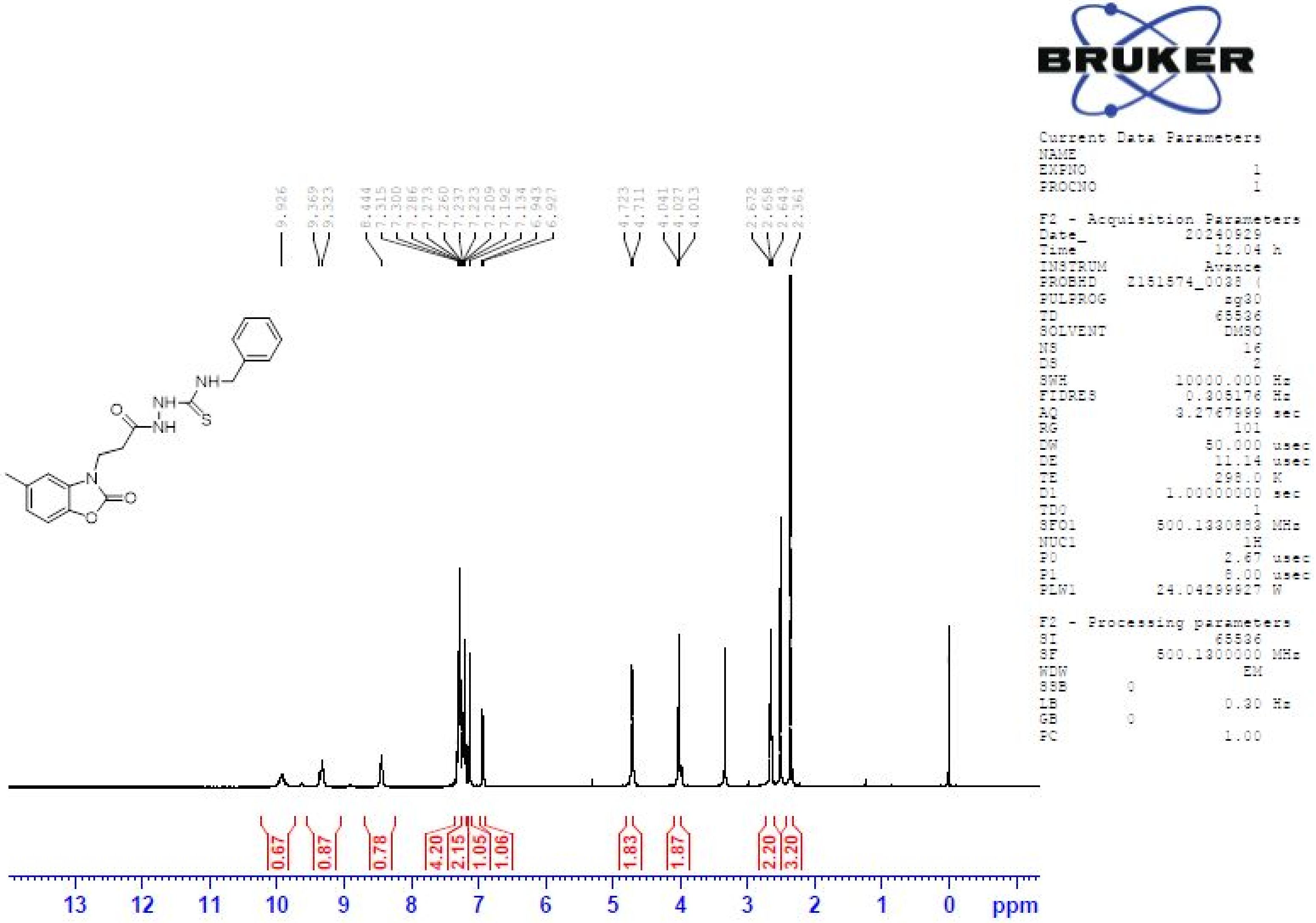

Figure S22. 1H-NMR spectrum of compound 4bk'

^{13}C-NMR spectra of 4aa'-k'/4ba'-k' compounds (S28-S49).

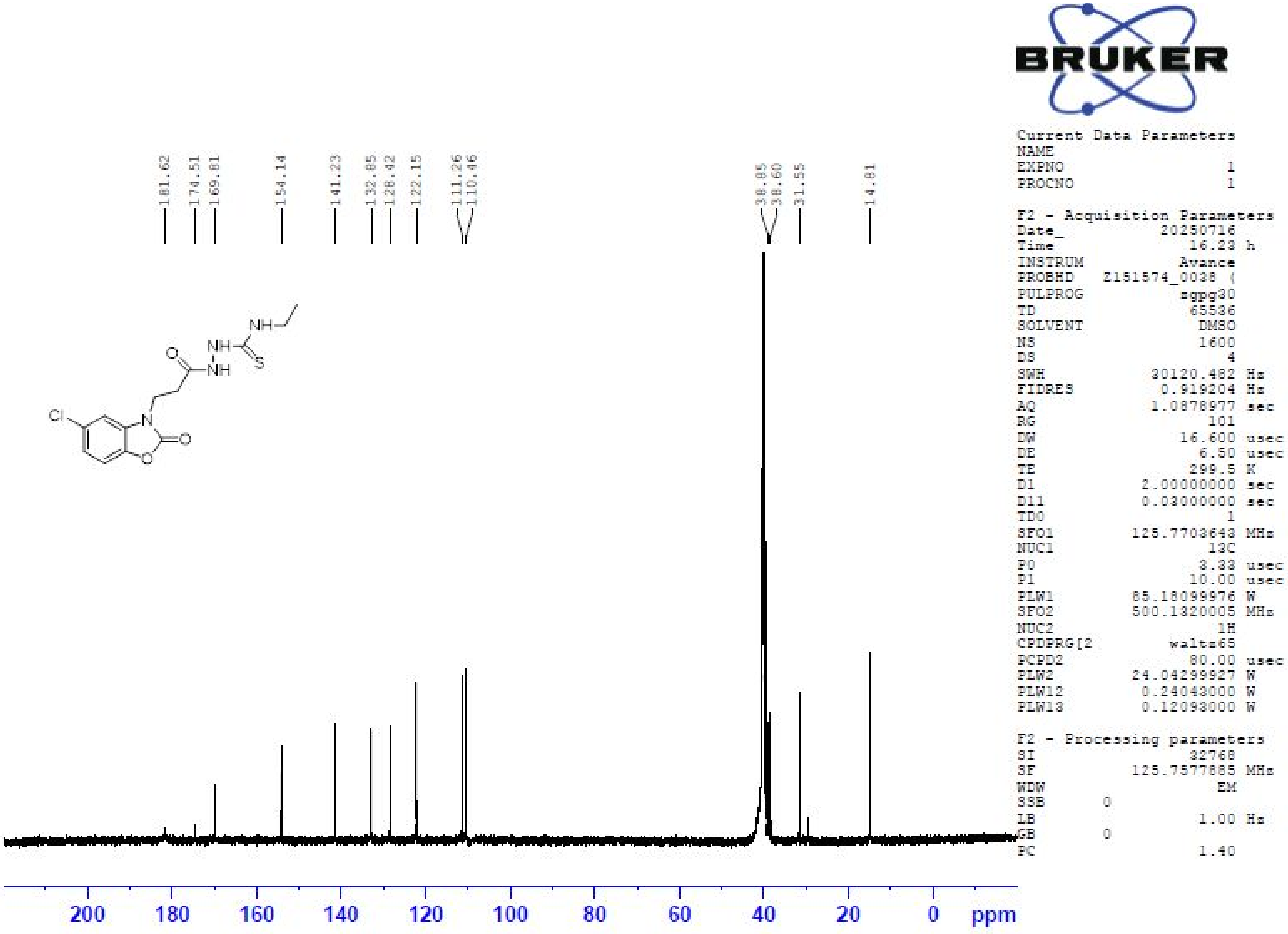

Figure S23. 13C-NMR spectrum of compound 4aa'

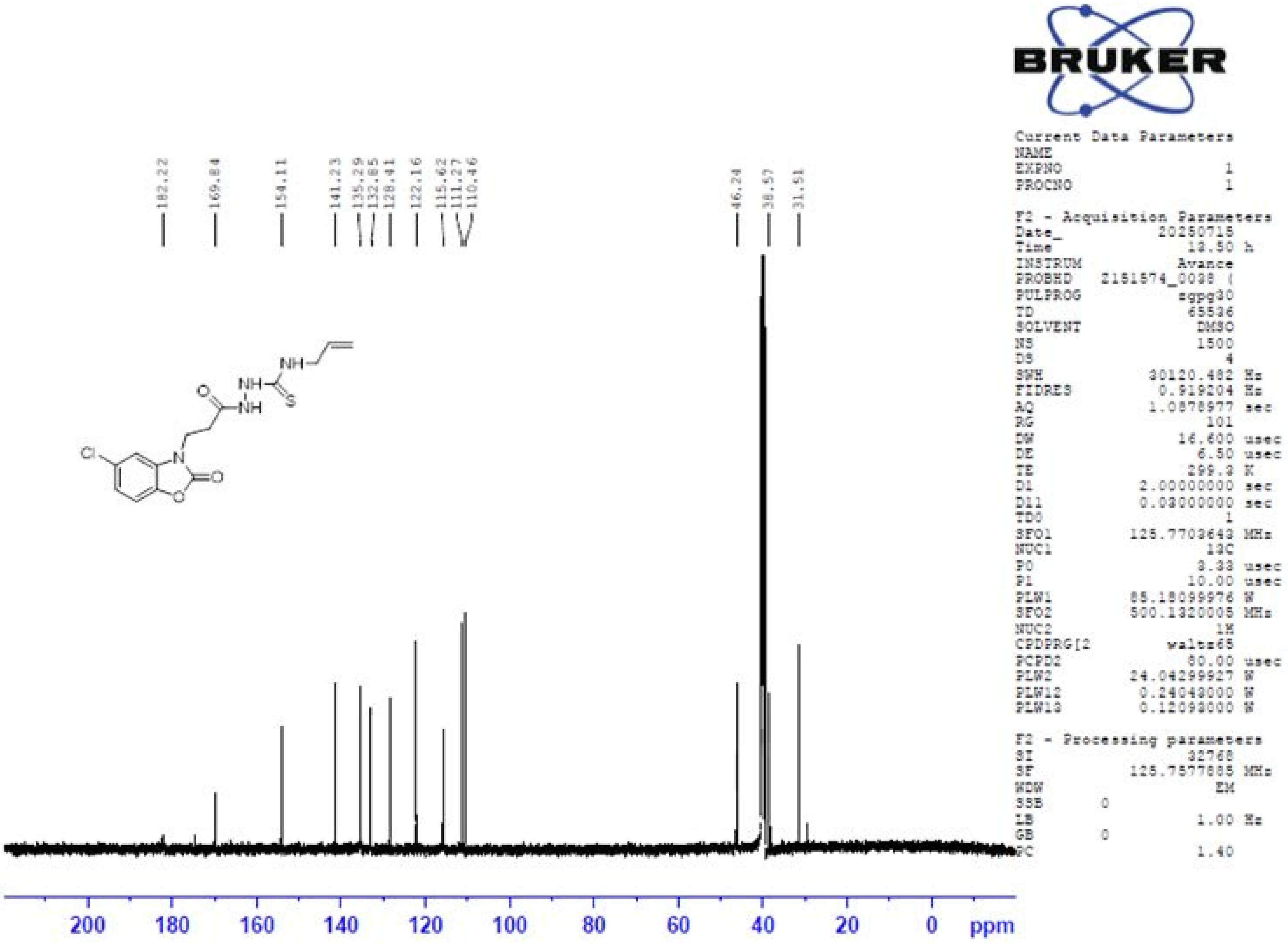

Figure S24. 13C-NMR spectrum of compound 4ab'

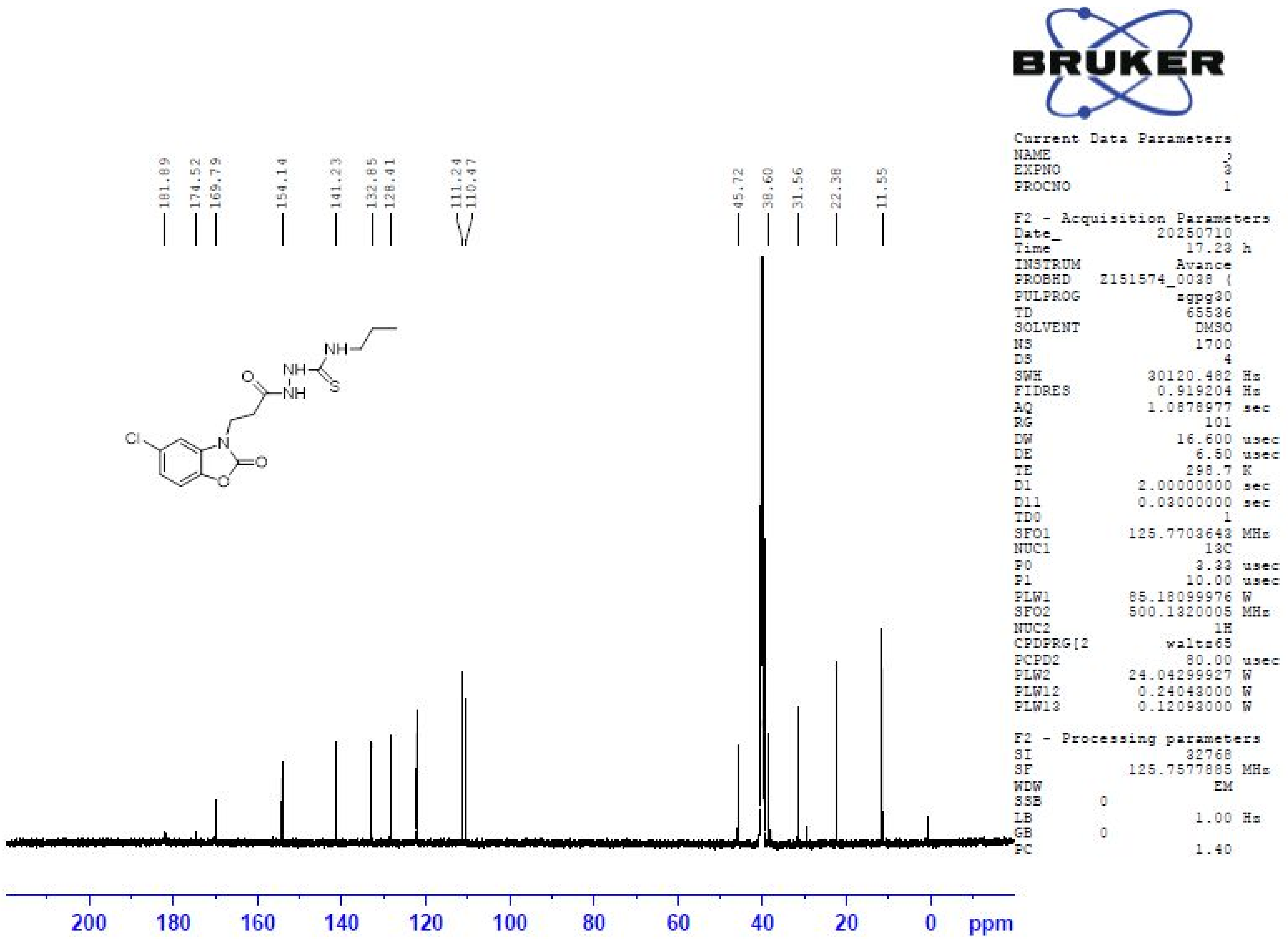

Figure S25. 13C-NMR spectrum of compound 4ac'

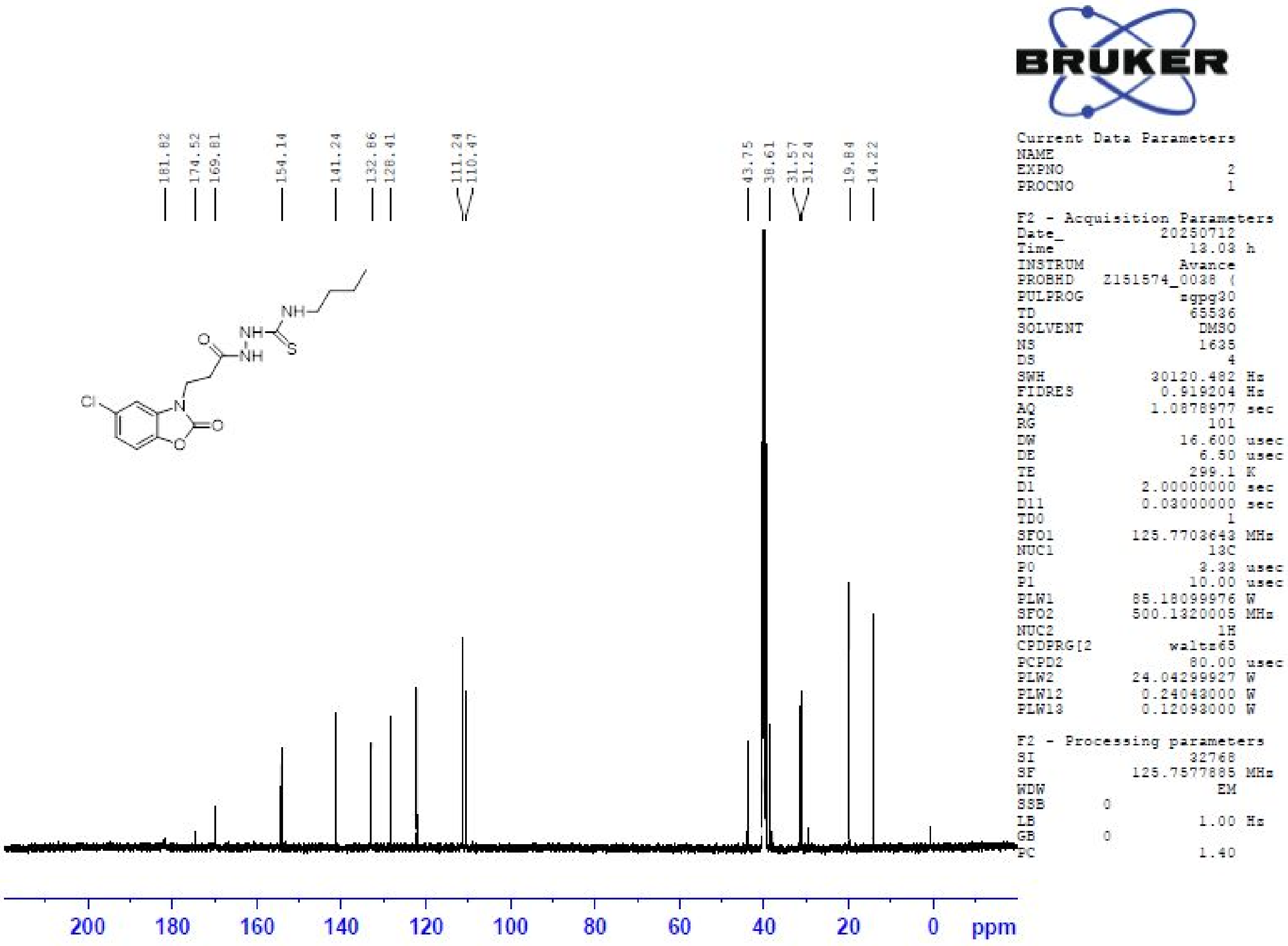

Figure S26. 13C-NMR spectrum of compound 4ad'

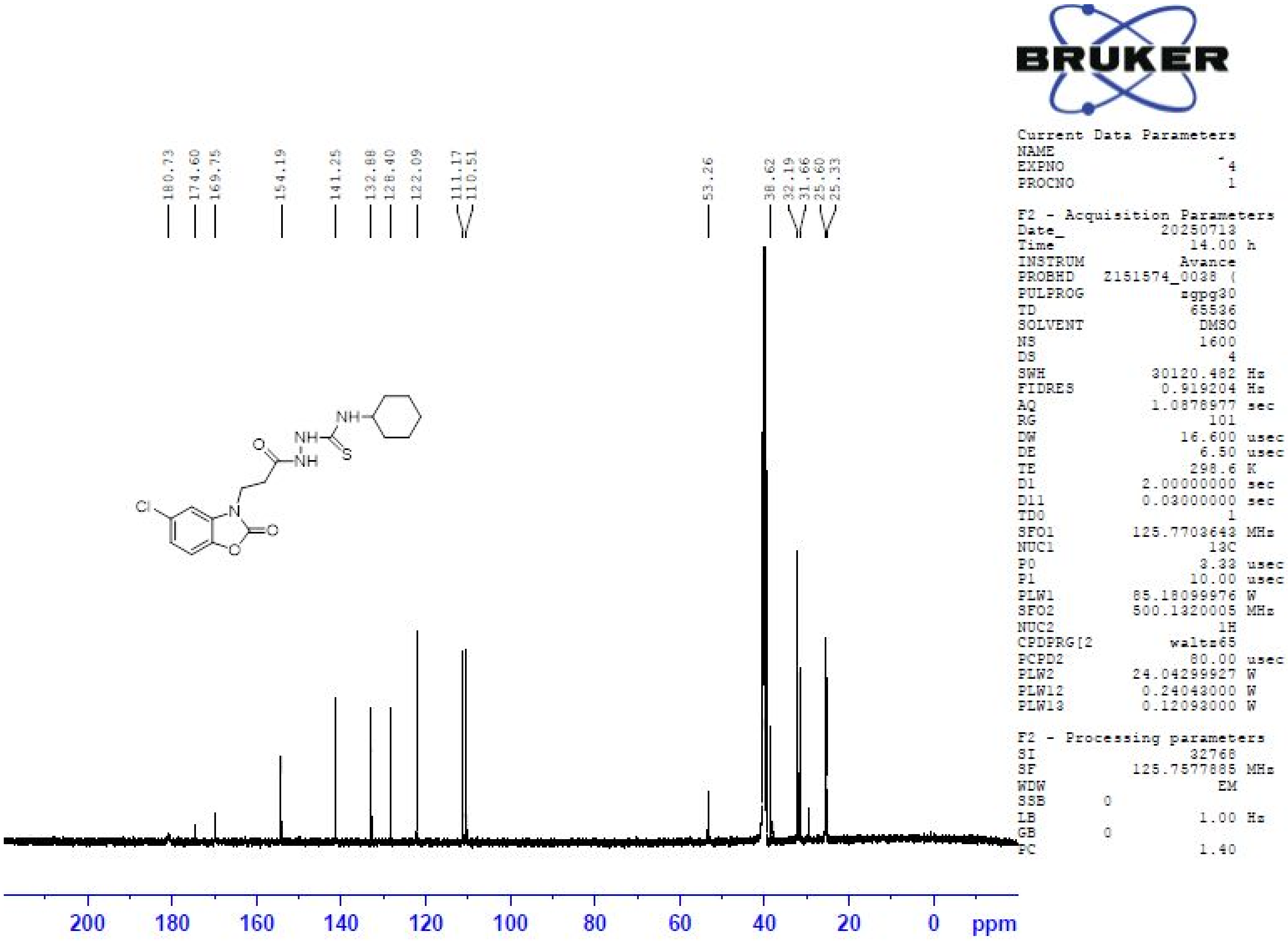

Figure S27. 13C-NMR spectrum of compound 4ae'

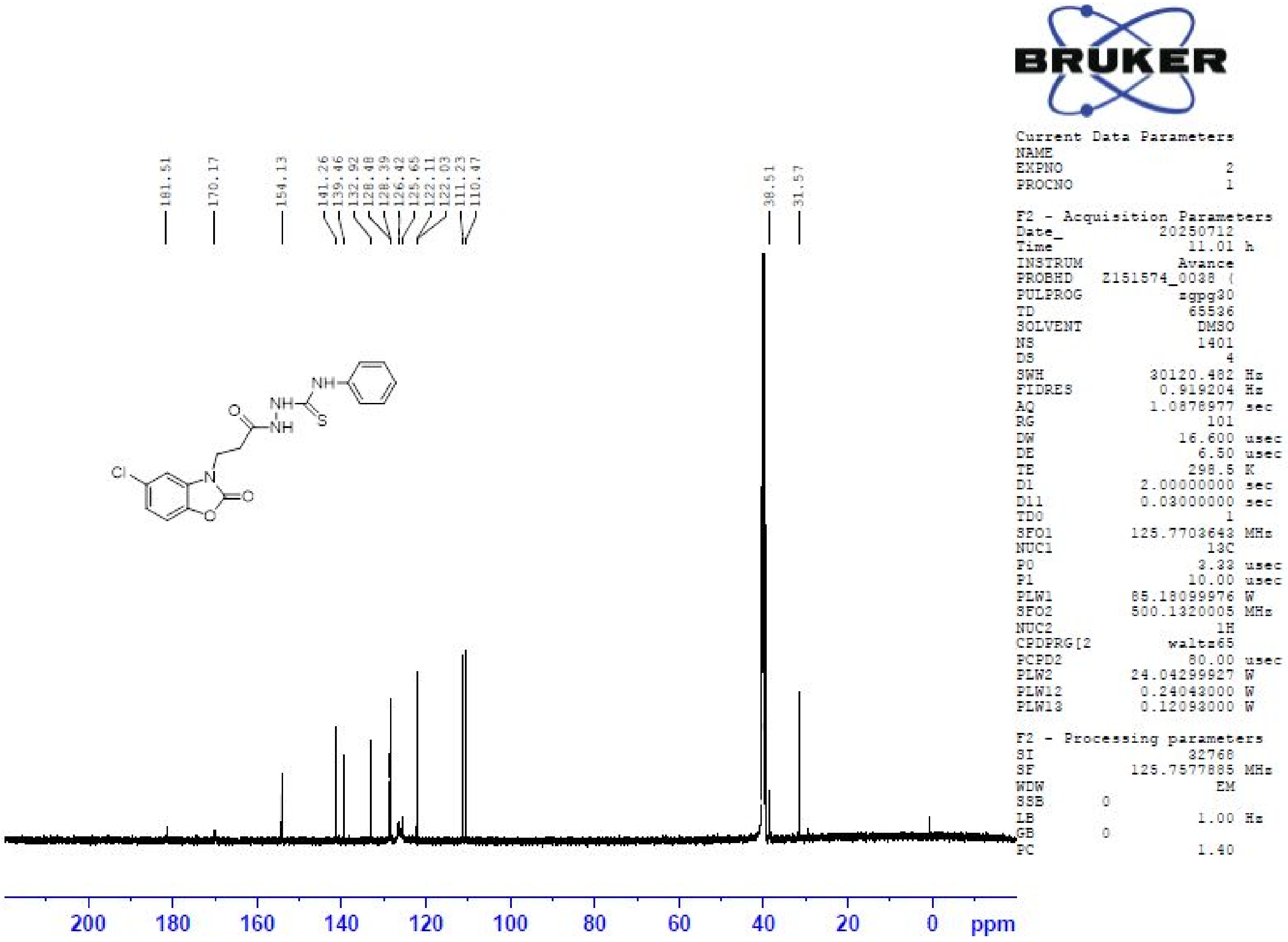

Figure S28. 13C-NMR spectrum of compound 4af'

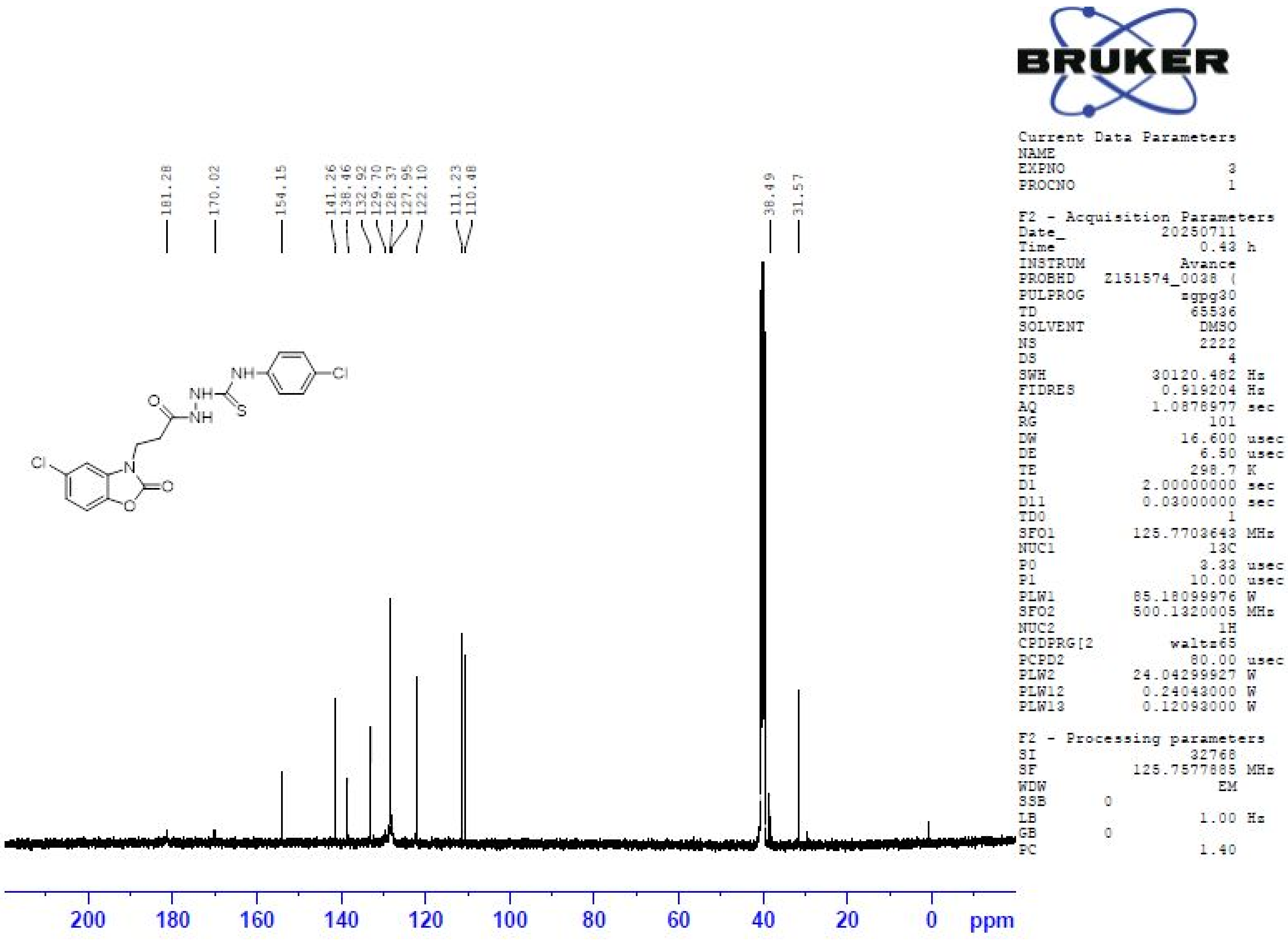

Figure S29. 13C-NMR spectrum of compound 4ag'

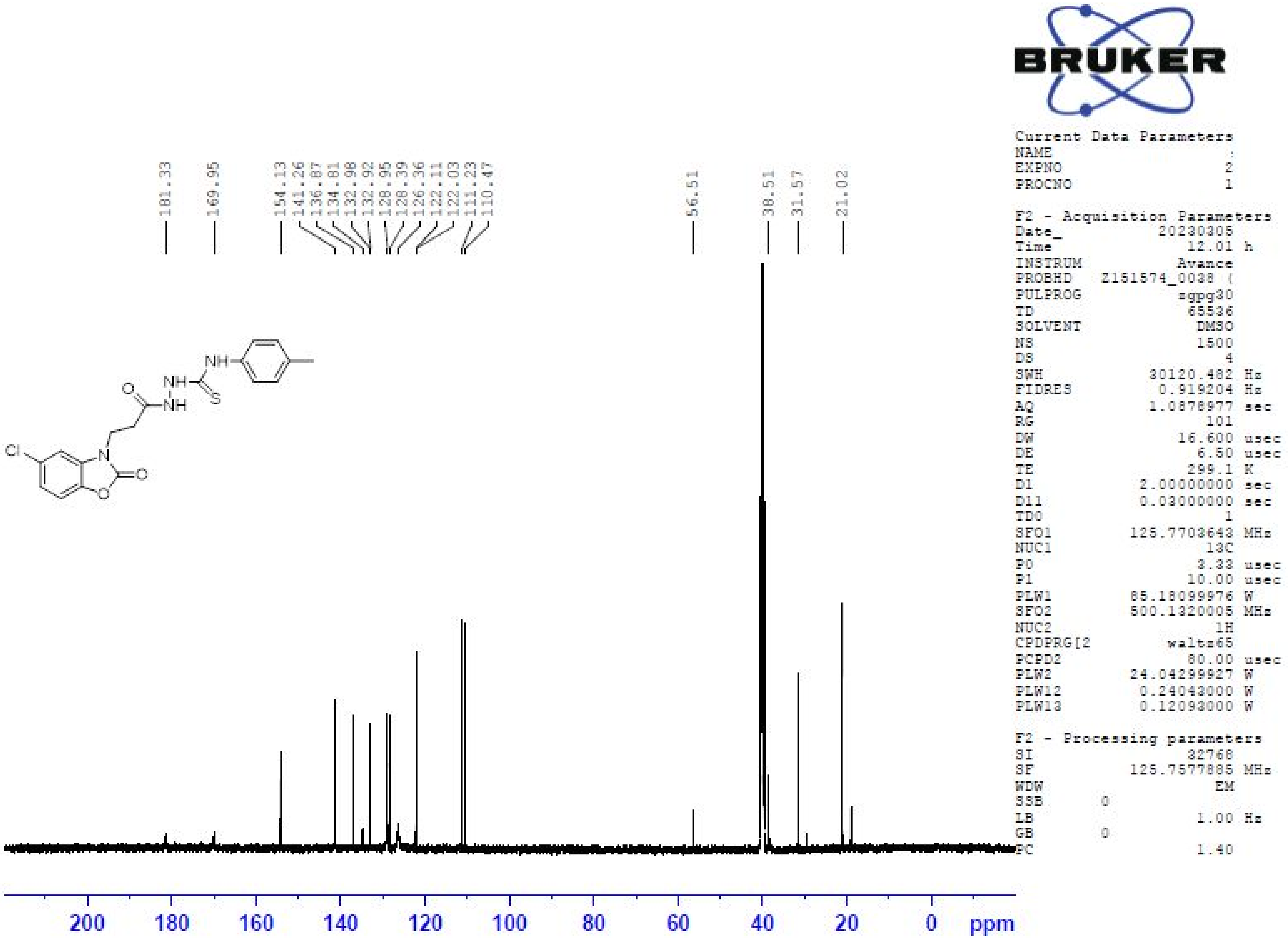

Figure S30. 13C-NMR spectrum of compound 4ah'

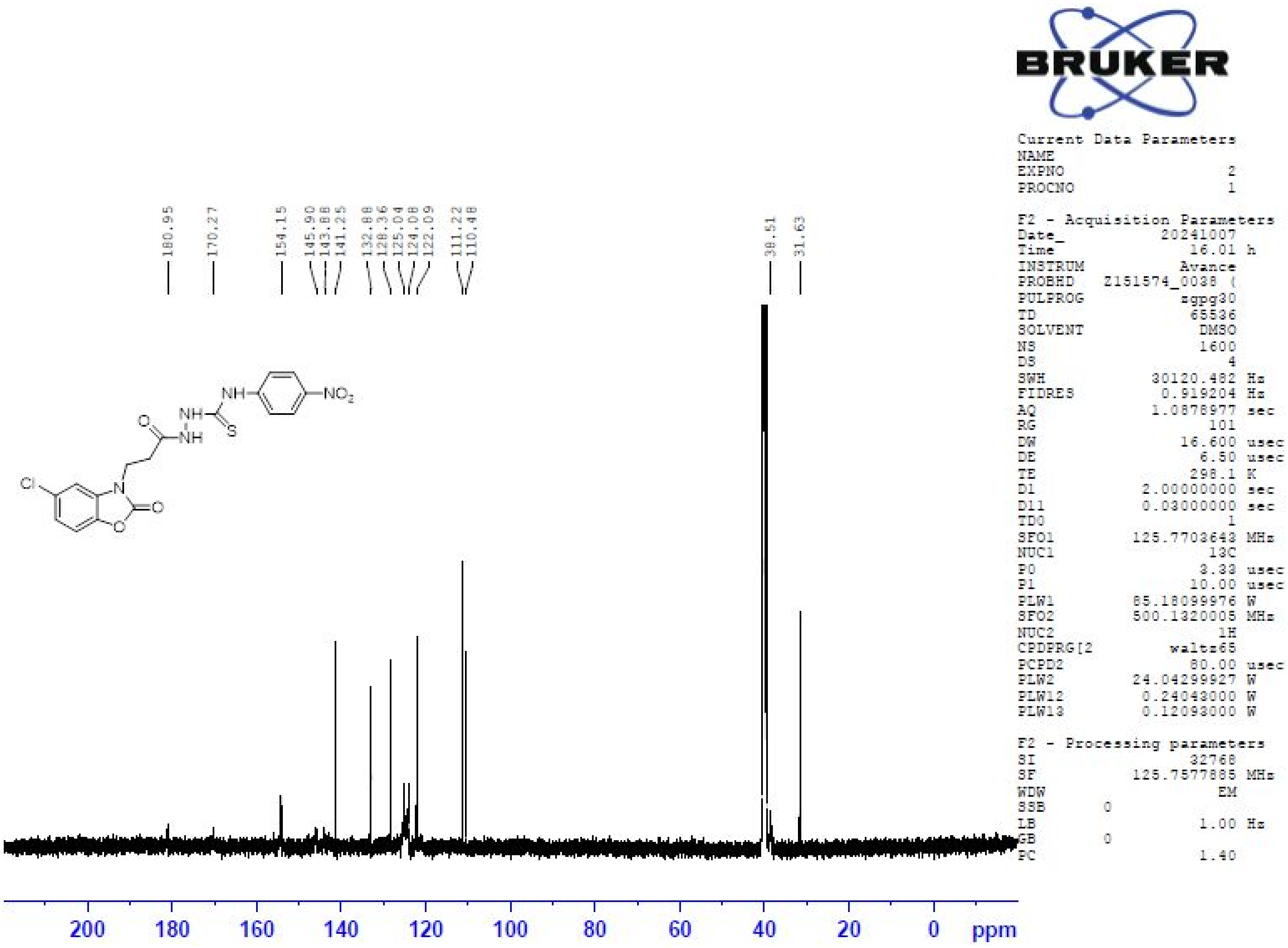

Figure S31. 13C-NMR spectrum of compound 4ai'

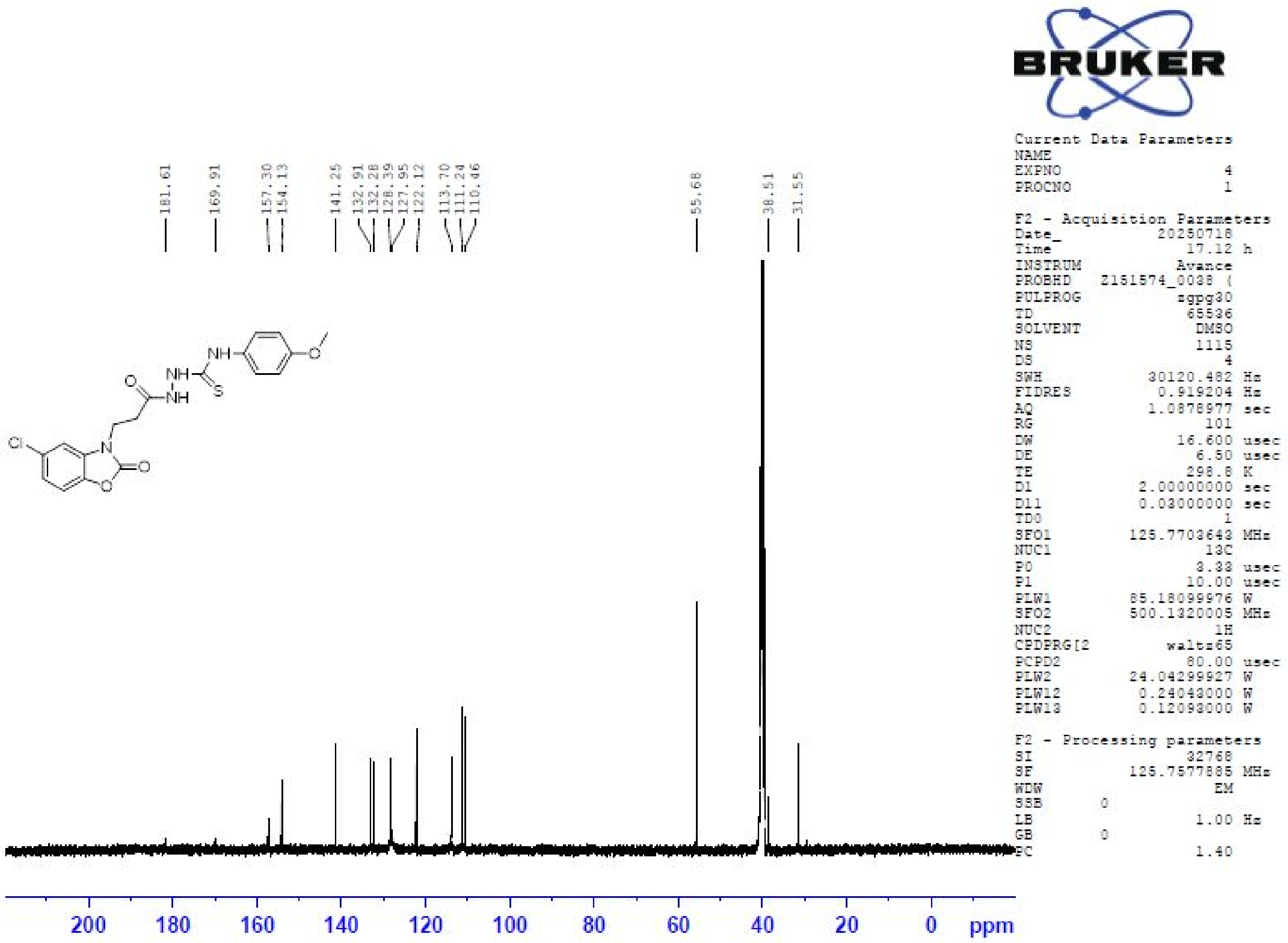

Figure S32. 13C-NMR spectrum of compound 4aj'

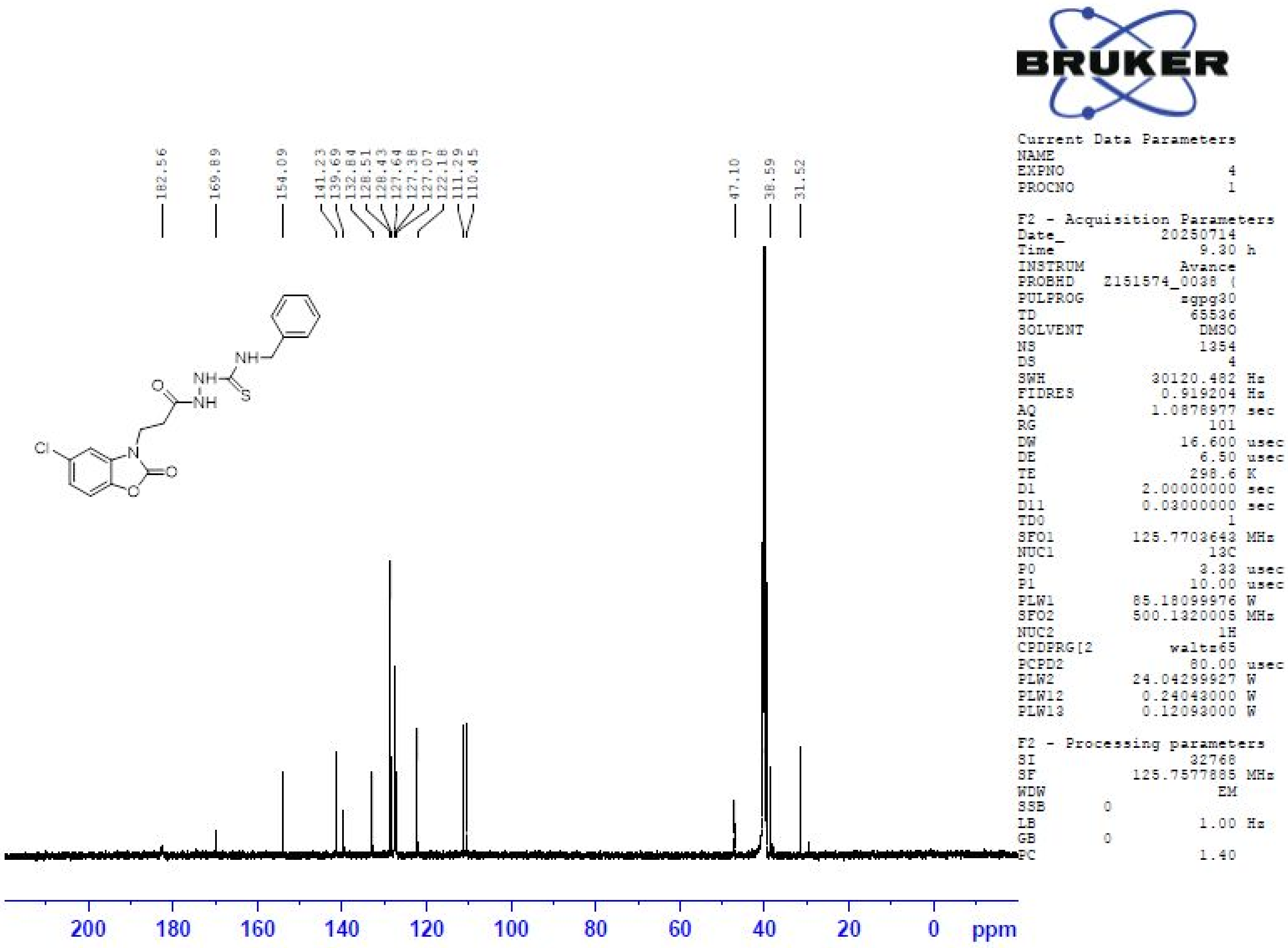

Figure S33. 13C-NMR spectrum of compound 4ak'

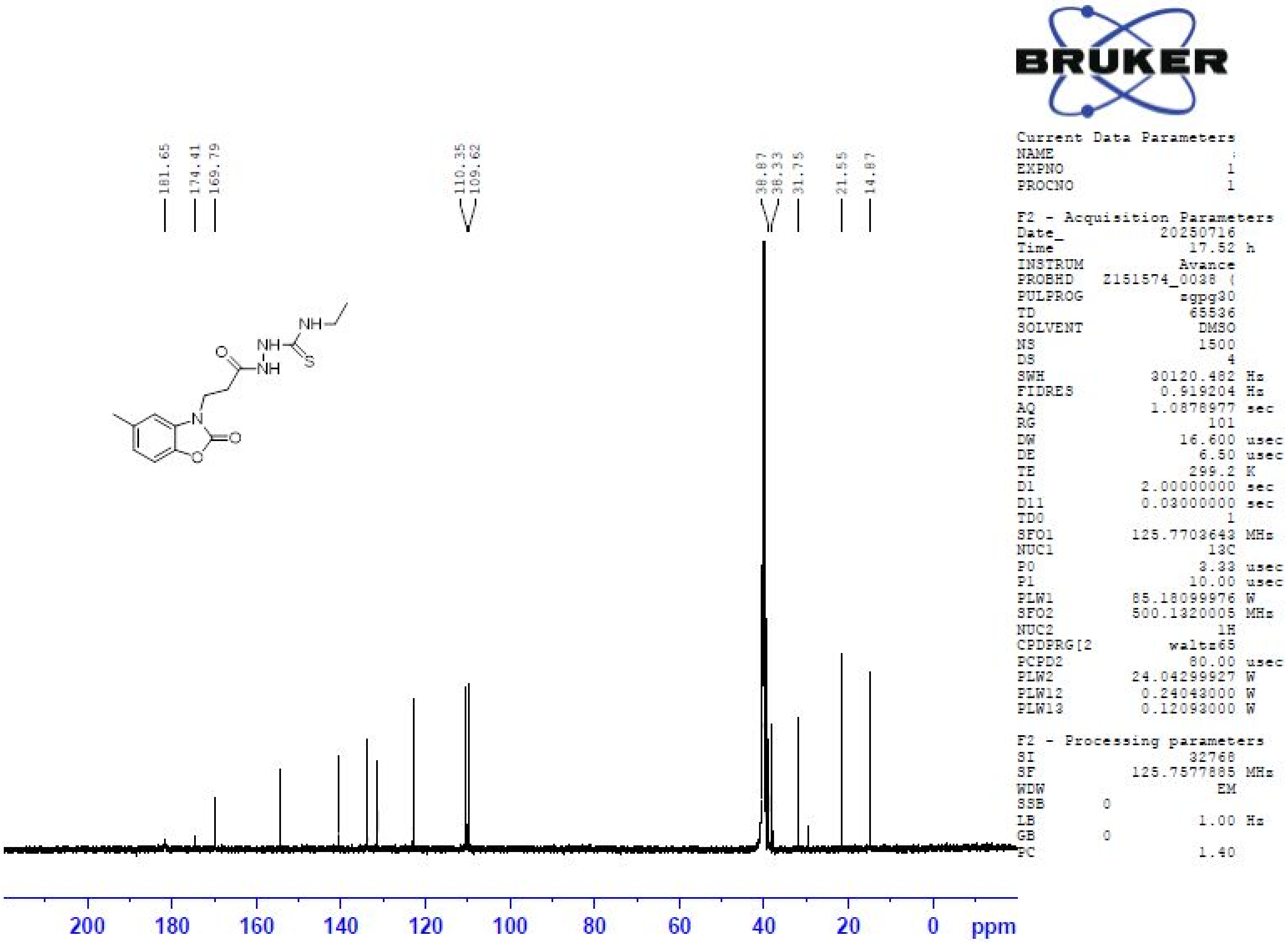

Figure S34. 13C-NMR spectrum of compound 4ba'

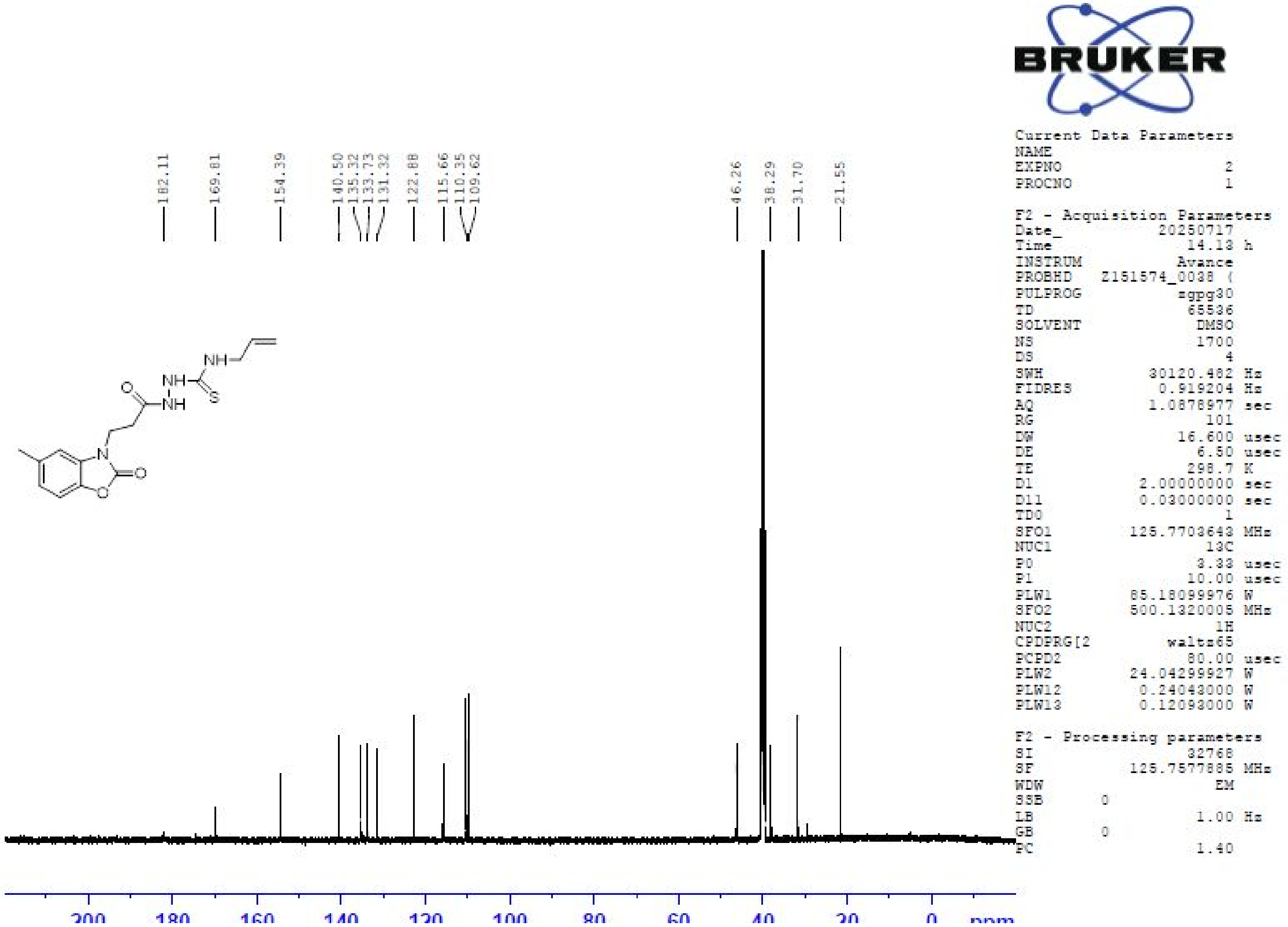

Figure S35. 13C-NMR spectrum of compound 4bb'

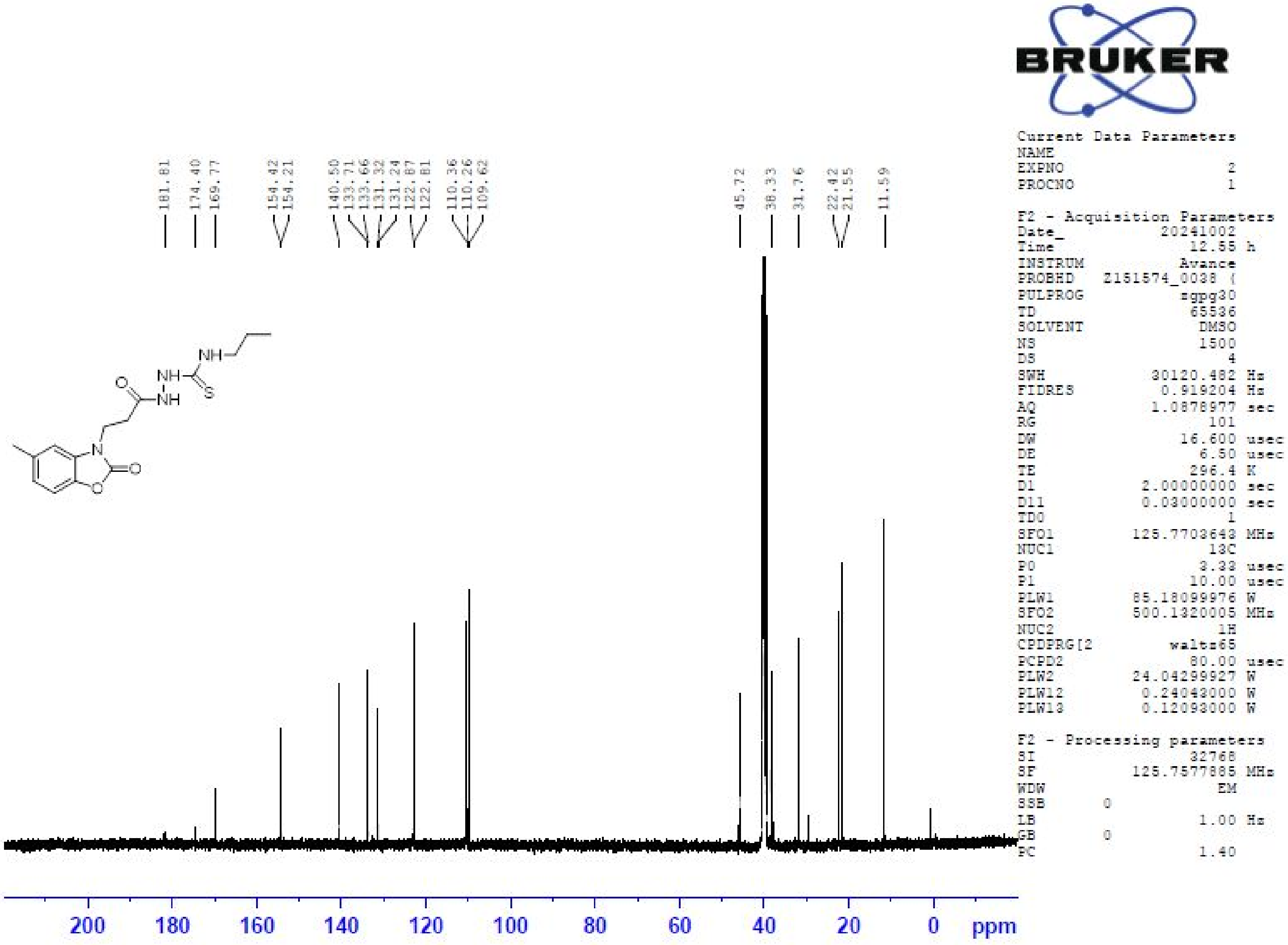

Figure S36. 13C-NMR spectrum of compound 4bc'

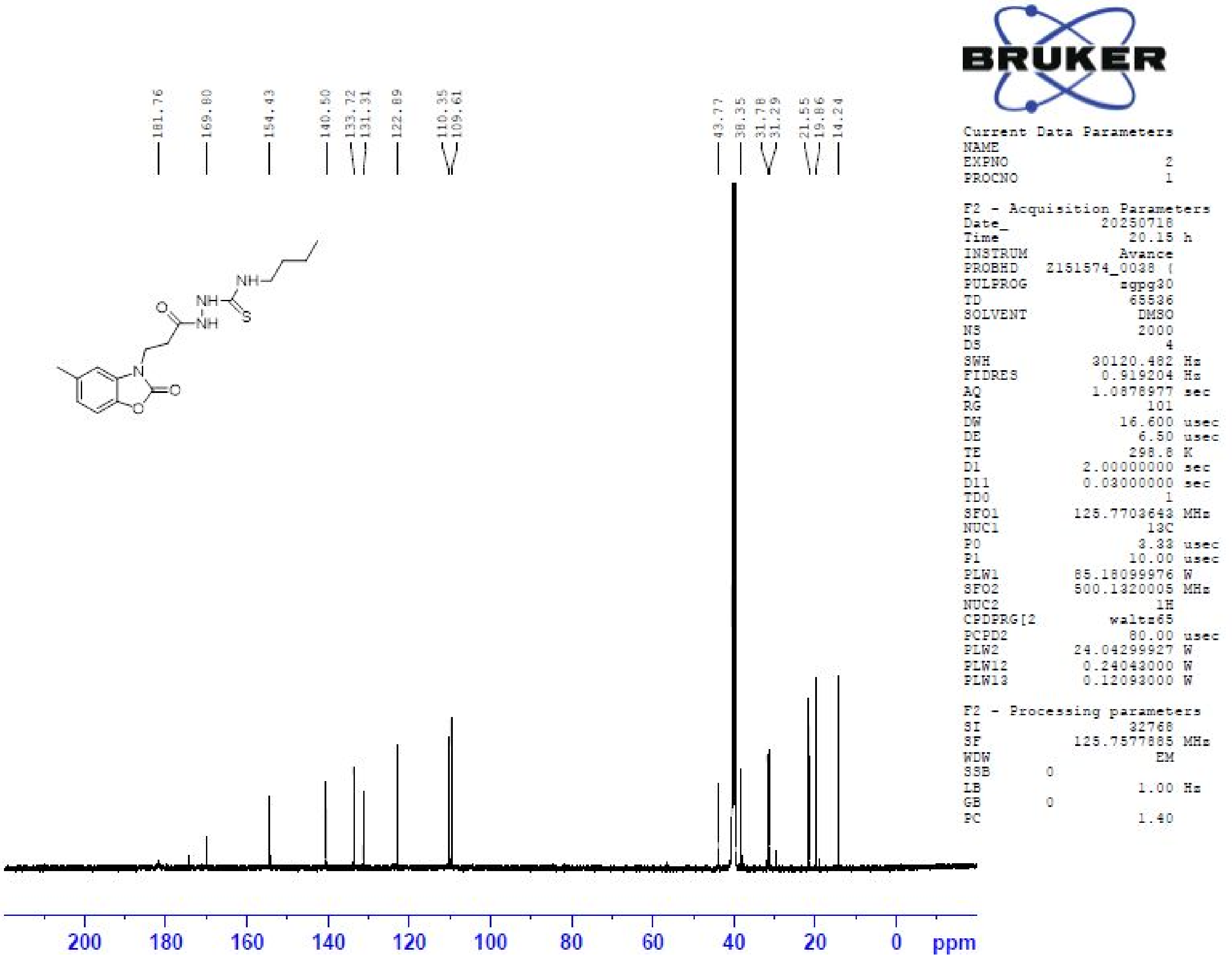

Figure S37. 13C-NMR spectrum of compound 4bd'

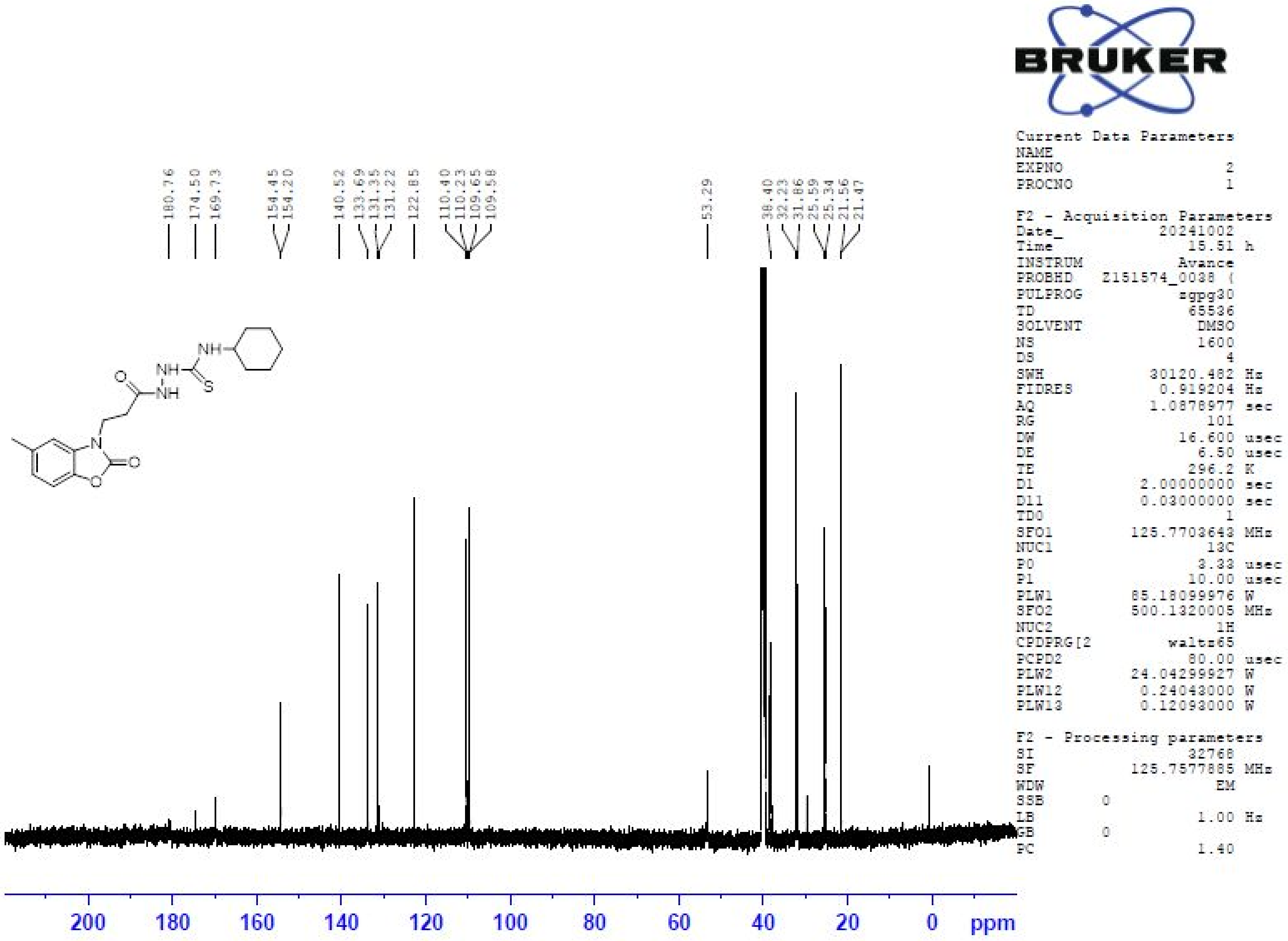

Figure S38. 13C-NMR spectrum of compound 4be'

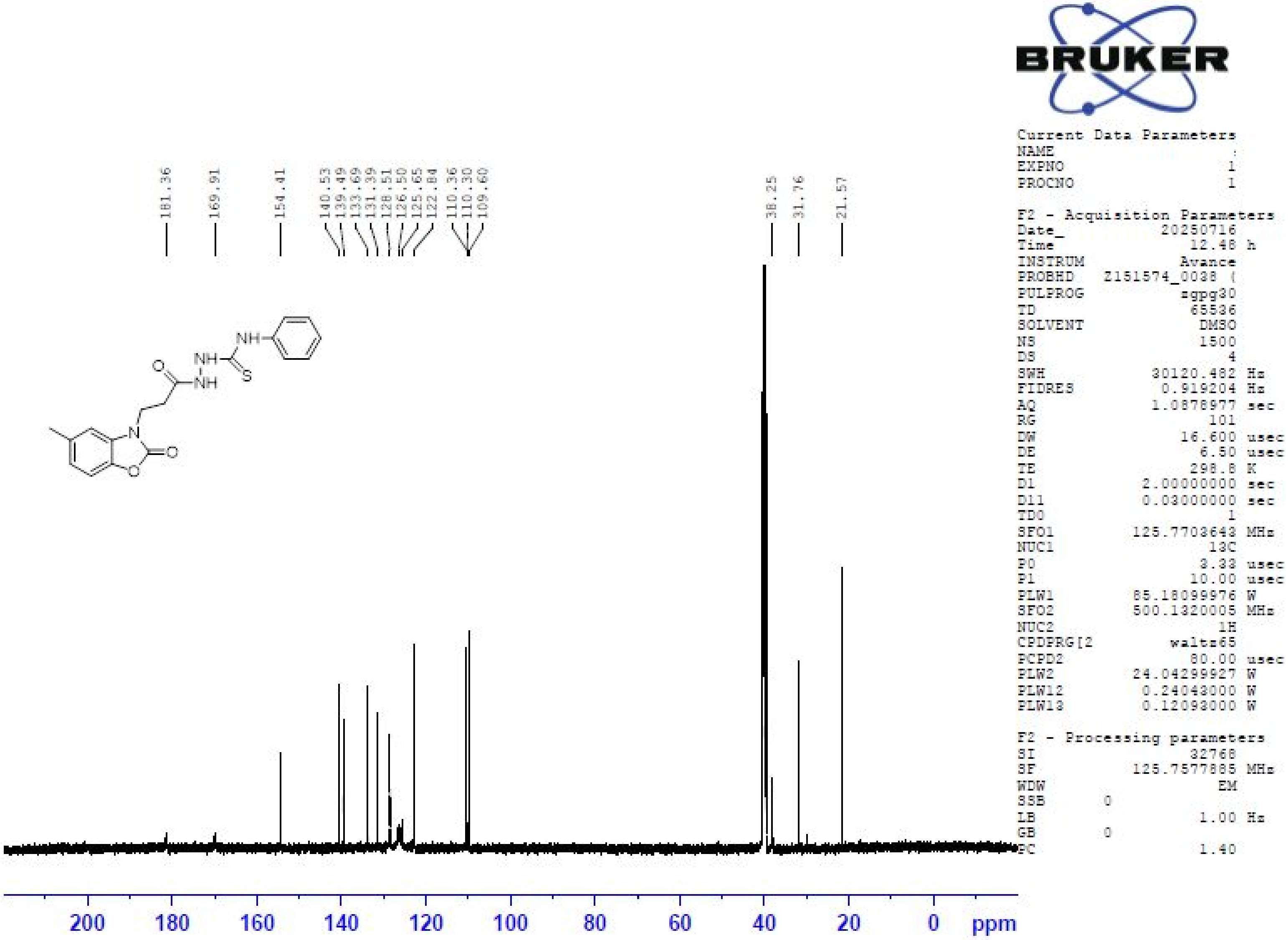

Figure S39. 13C-NMR spectrum of compound 4bf'

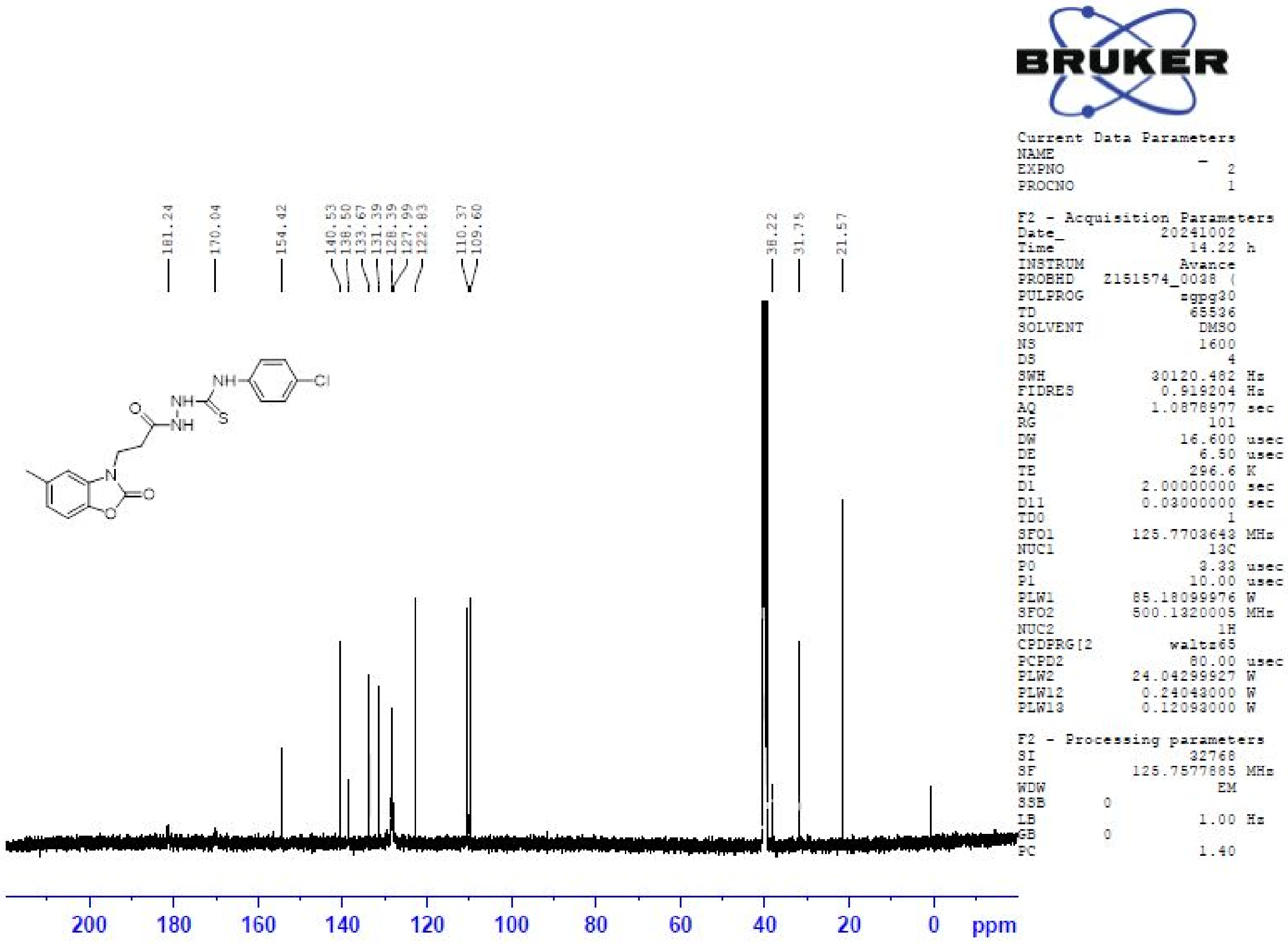

Figure S40. 13C-NMR spectrum of compound 4bg'

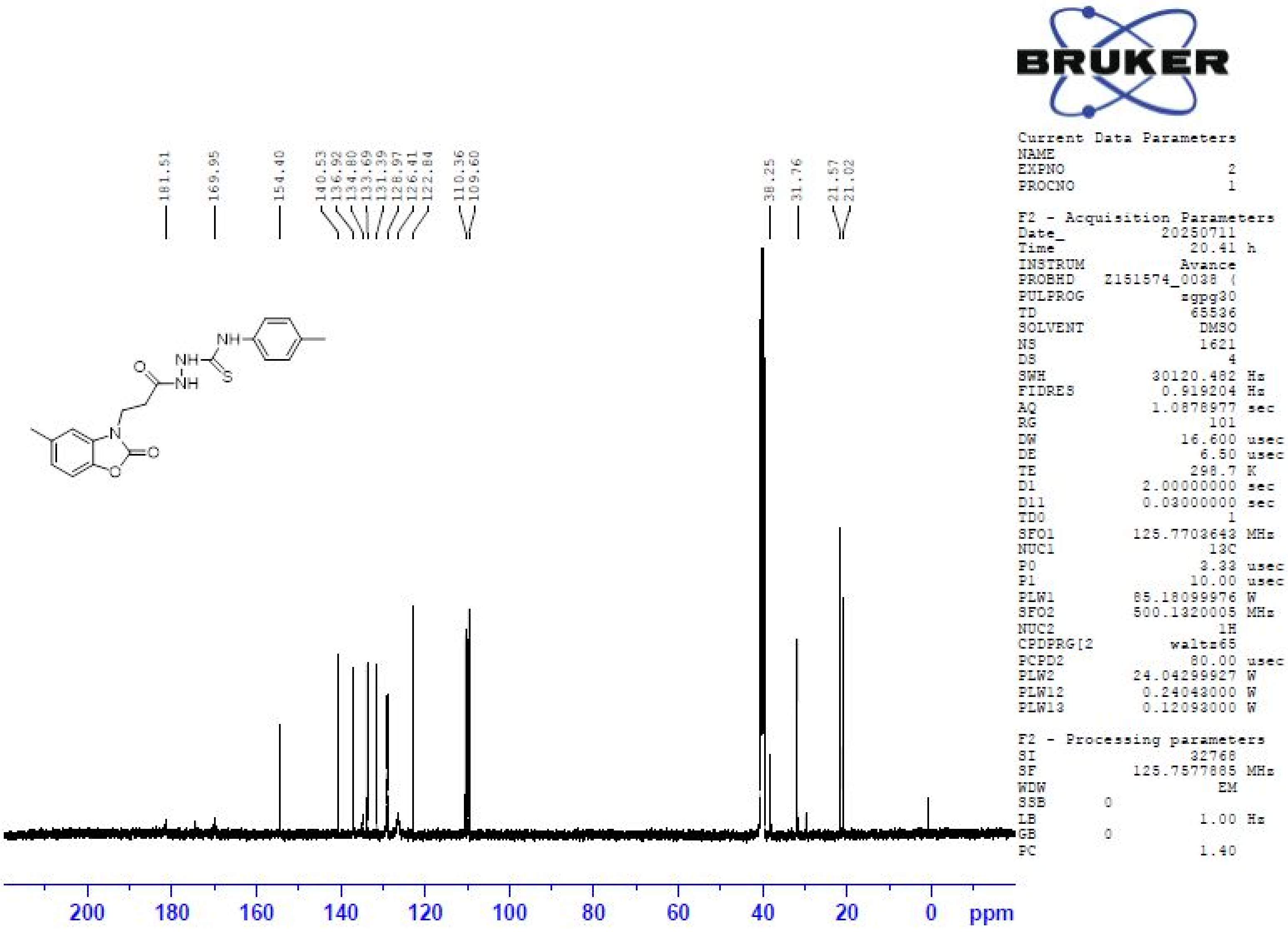

Figure S41. 13C-NMR spectrum of compound 4bh'

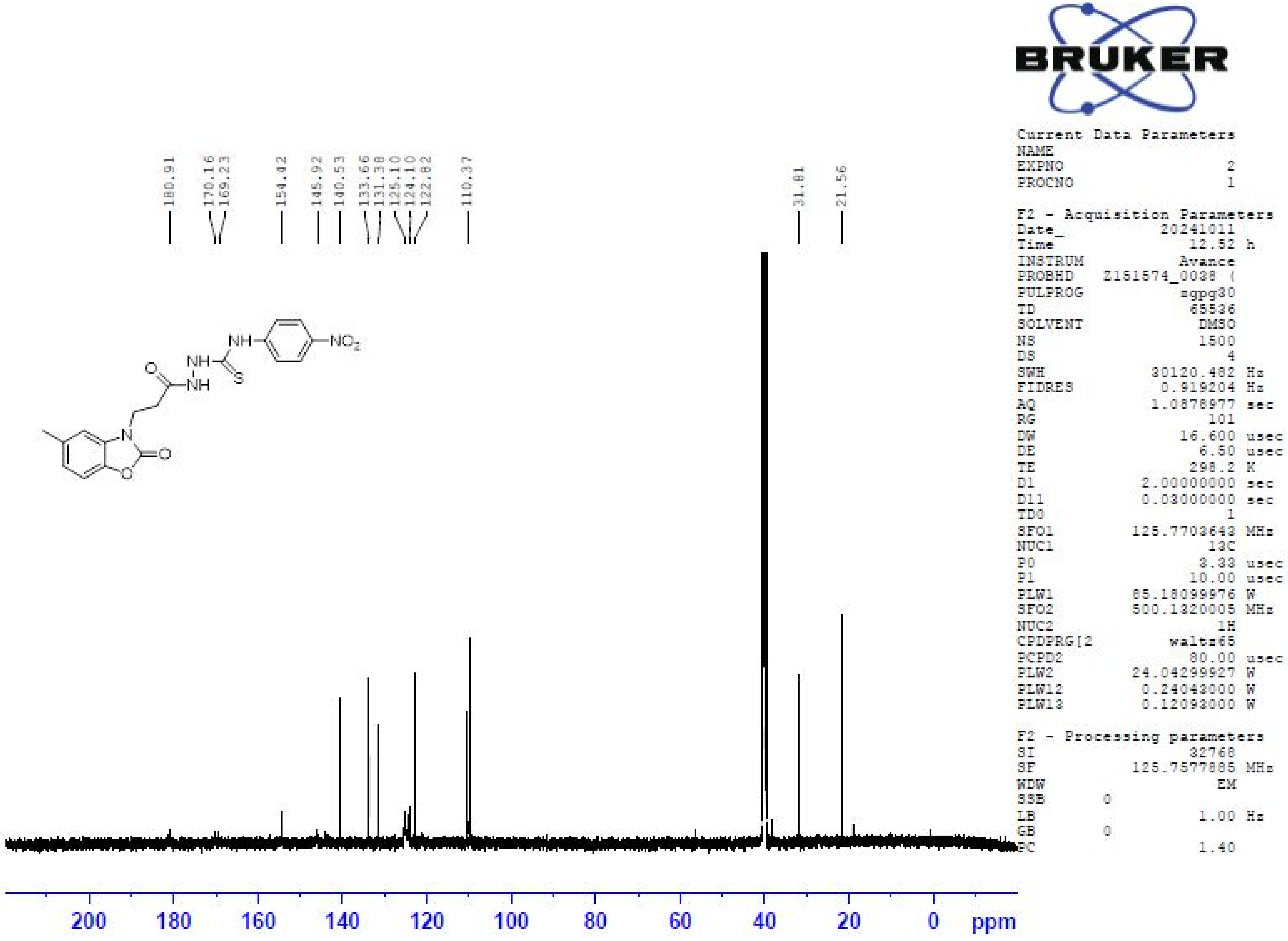

Figure S42. 13C-NMR spectrum of compound 4bi'

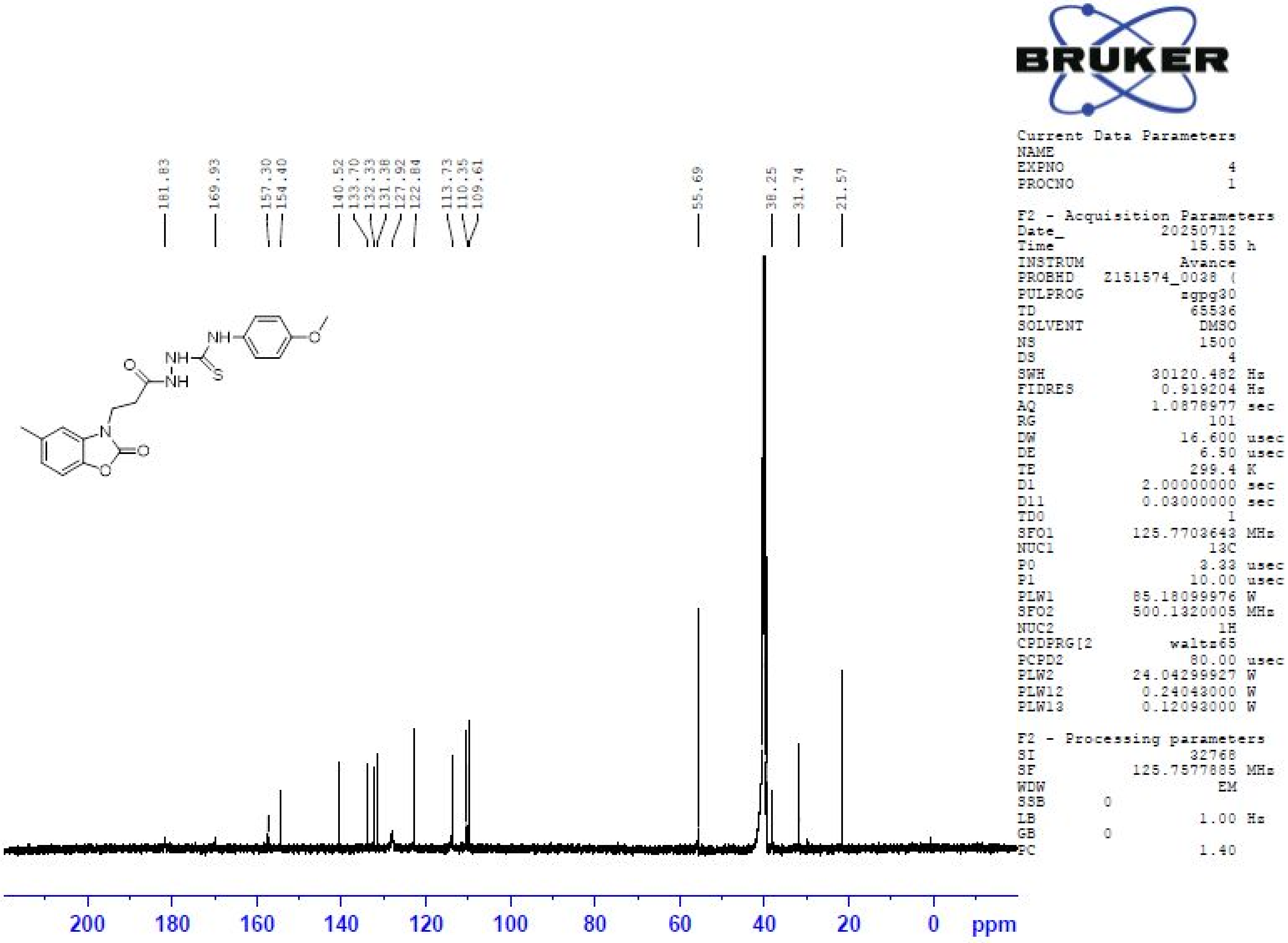

Figure S43. 13C-NMR spectrum of compound 4bj'

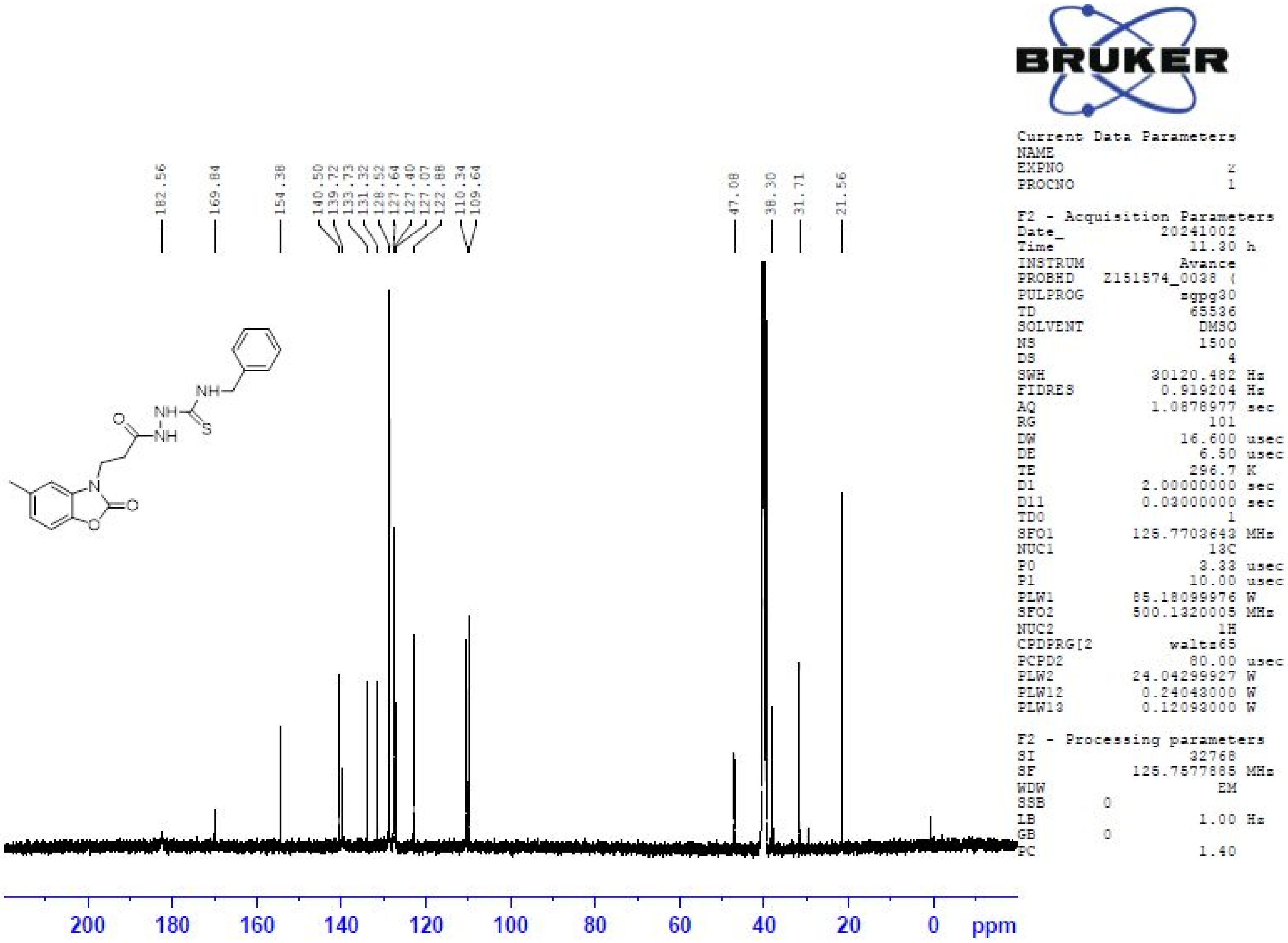

Figure S44. 13C-NMR spectrum of compound 4bk'

HRMS spectra of 4aa'-k'/4ba'-k' compounds (S50-S57).

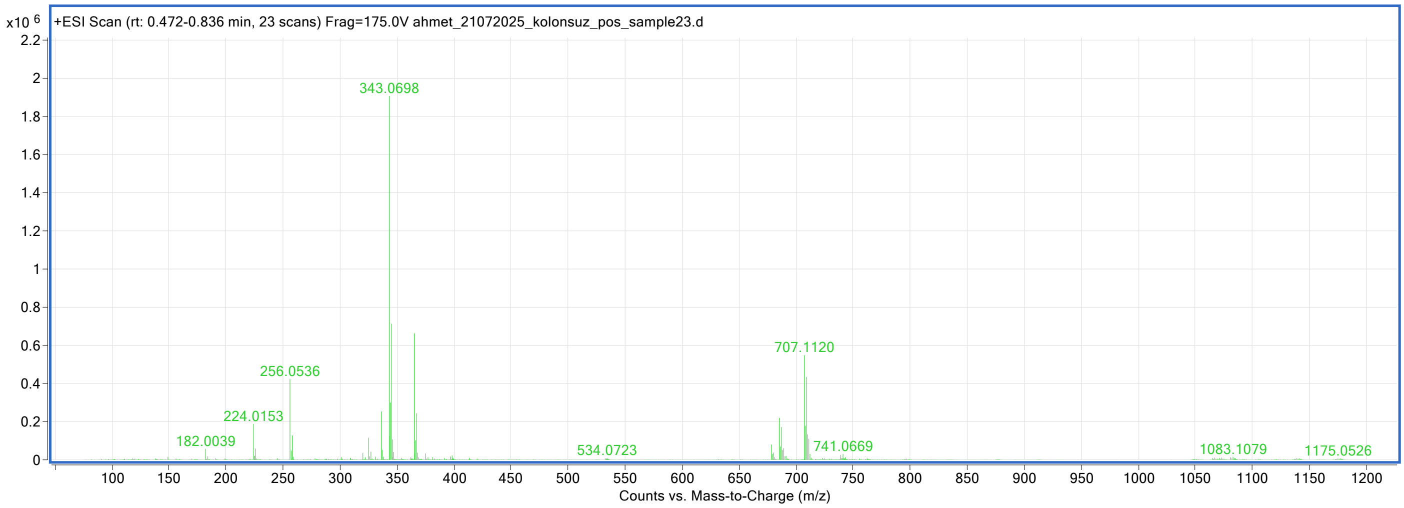

Figure S45. HRMS spectrum of compound 4aa'

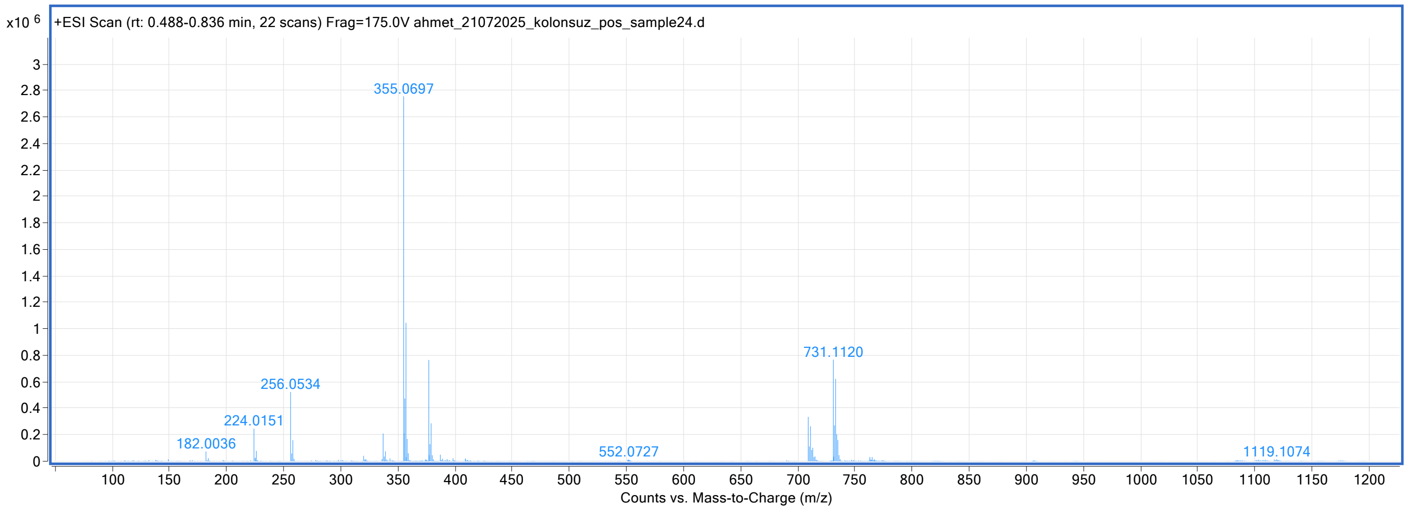

Figure S46. HRMS spectrum of compound 4ab'

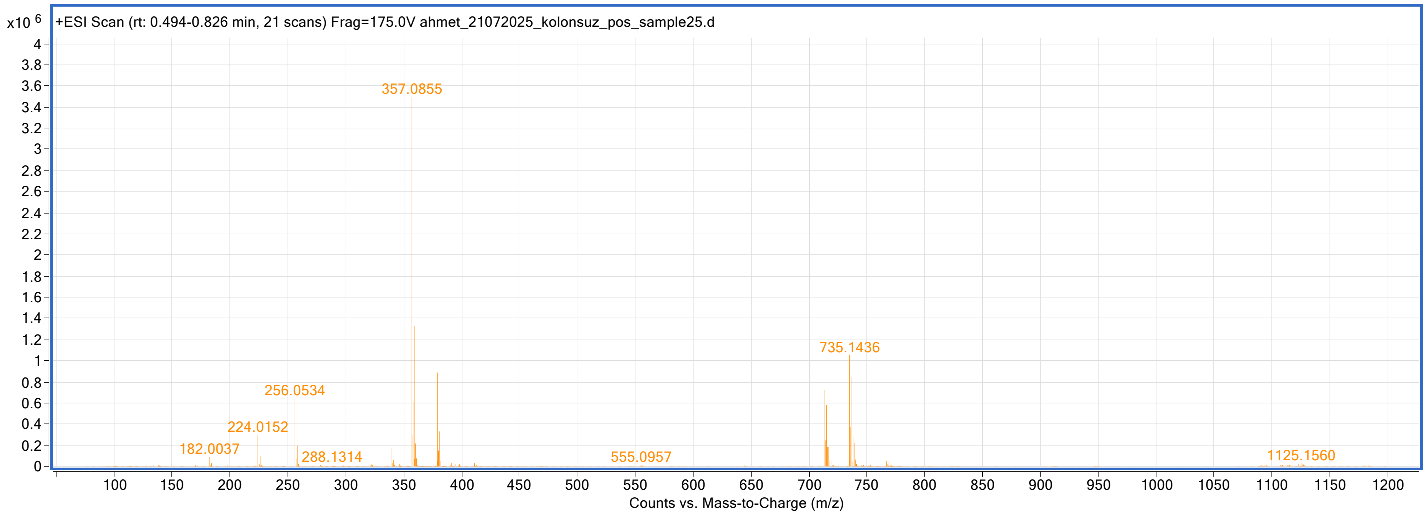

Figure S47. HRMS spectrum of compound 4ac'

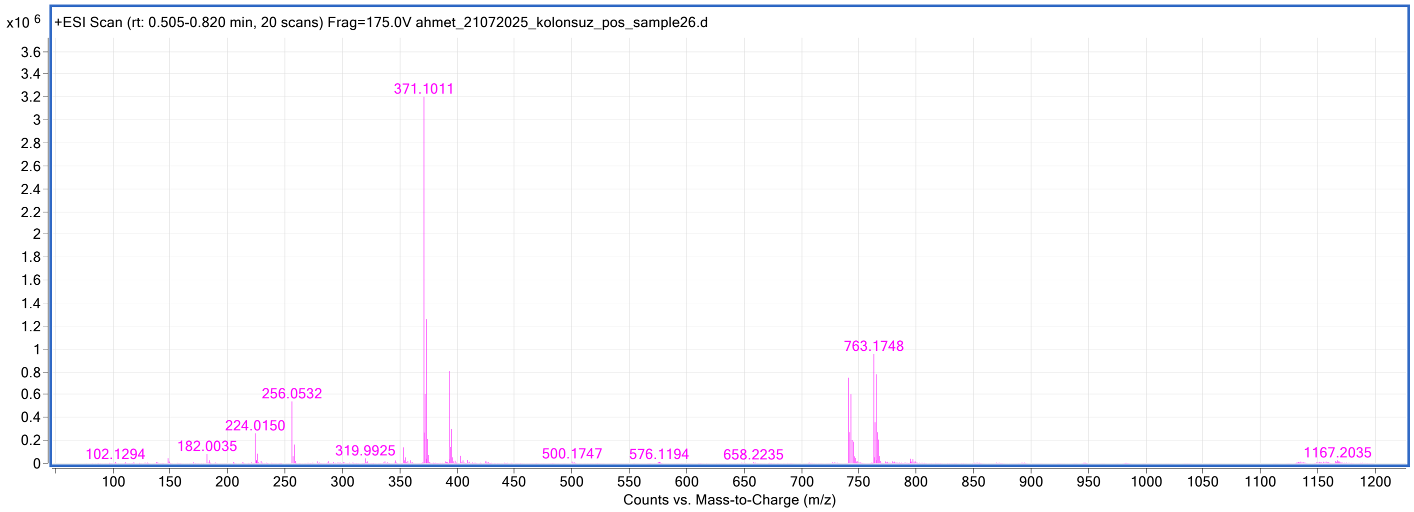

Figure S48. HRMS spectrum of compound 4ad'

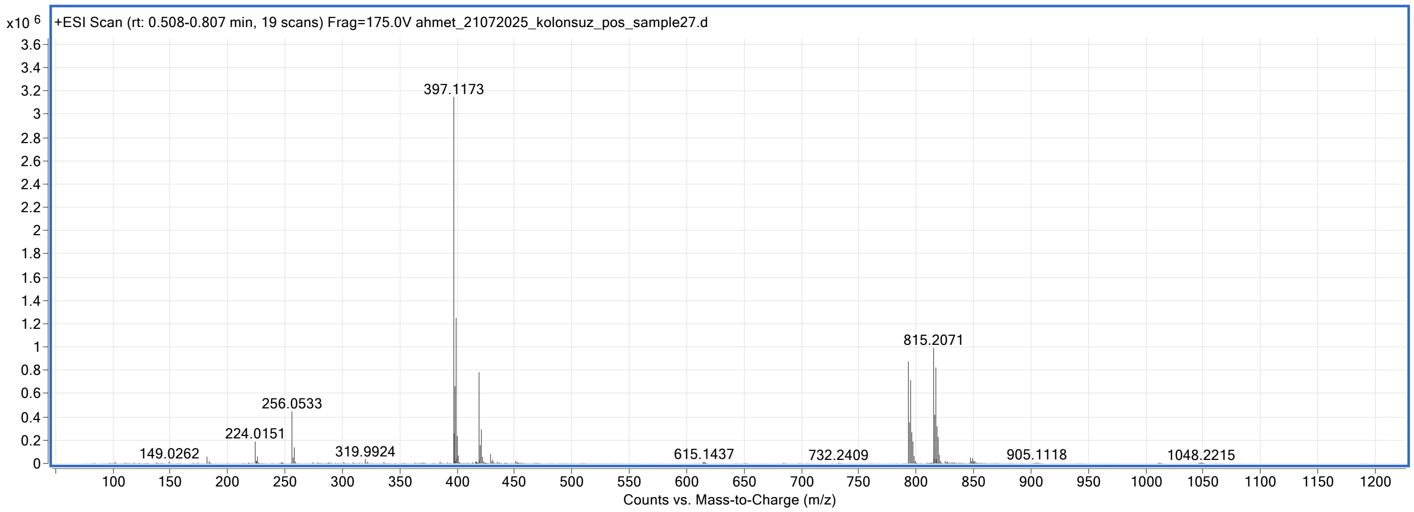

Figure S49. HRMS spectrum of compound 4ae'

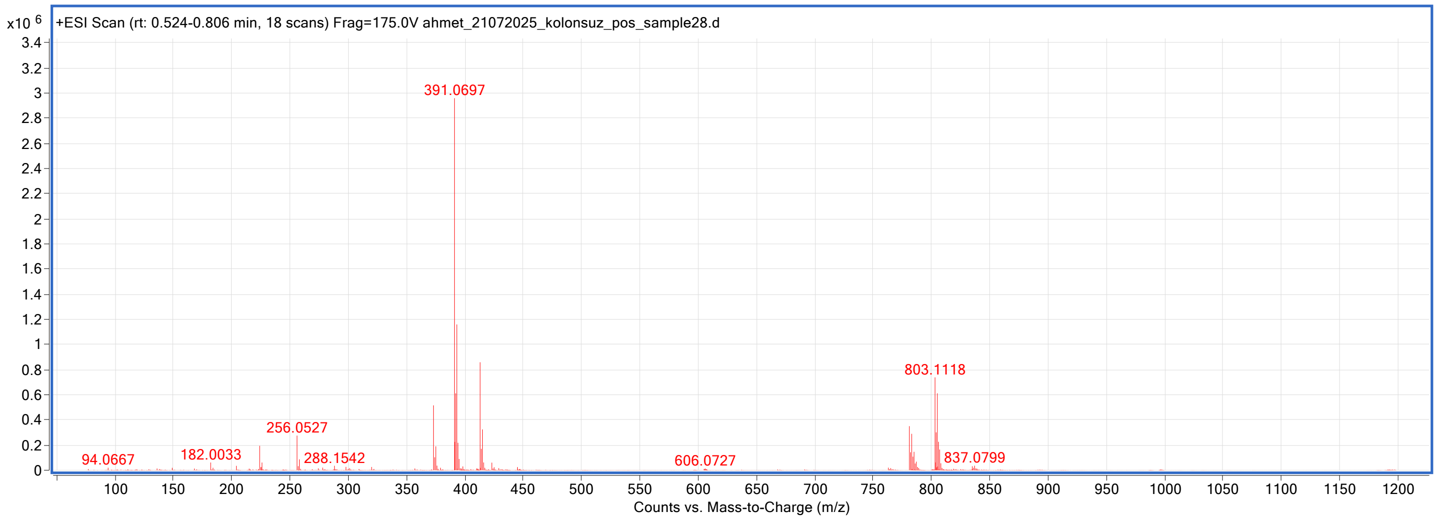

Figure S50. HRMS spectrum of compound 4af'

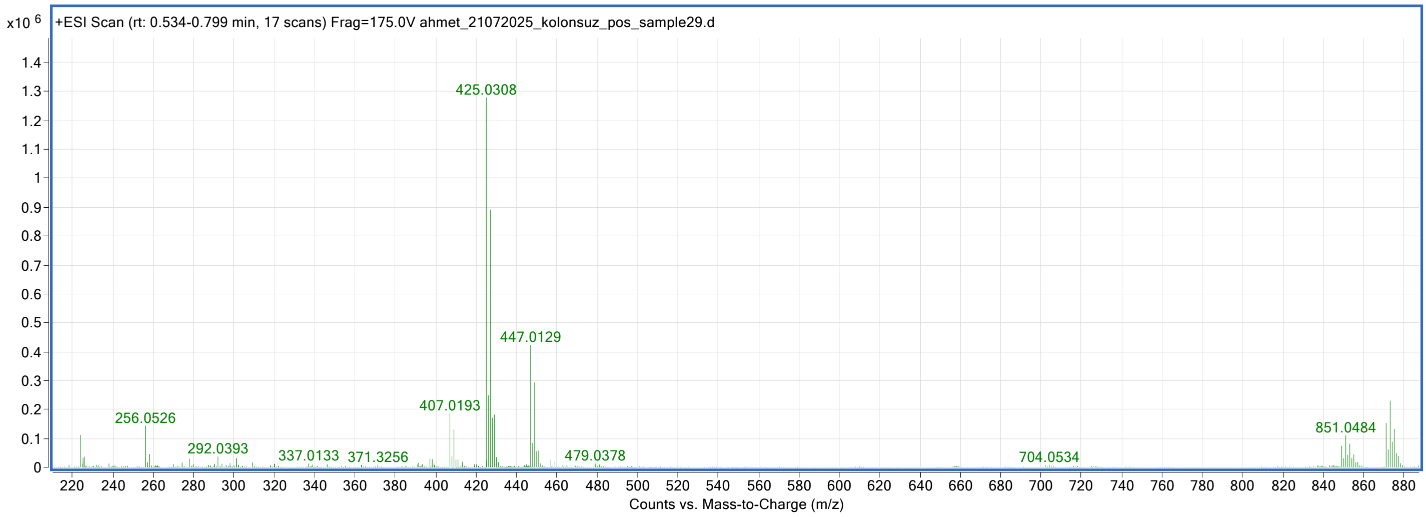

Figure S51. HRMS spectrum of compound 4ag'

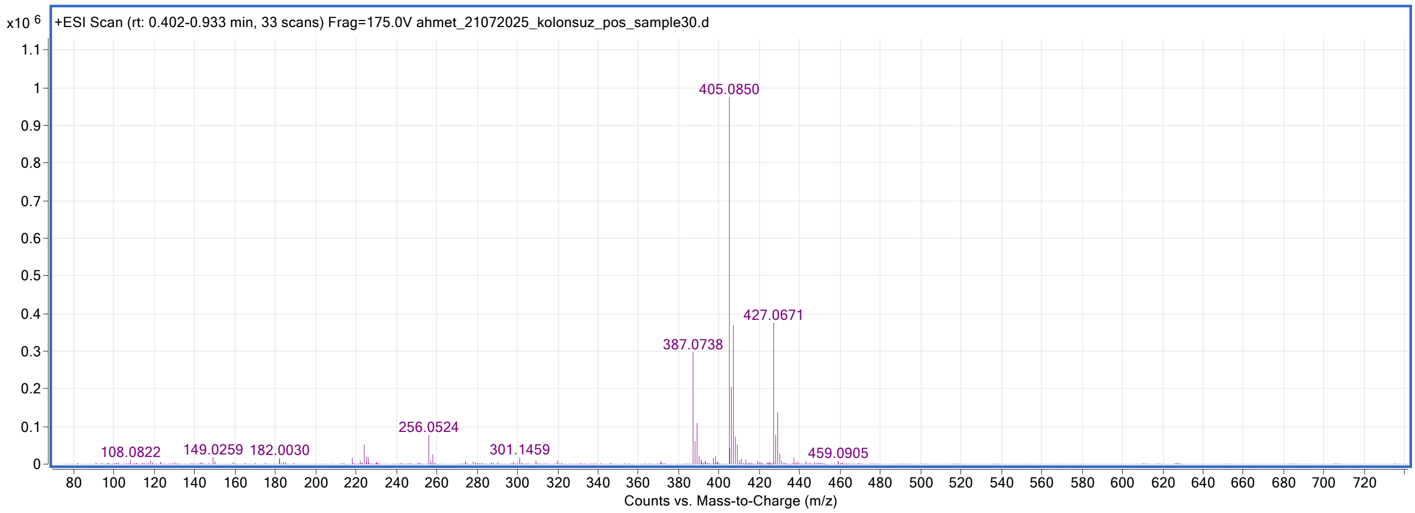

Figure S52. HRMS spectrum of compound 4ah'

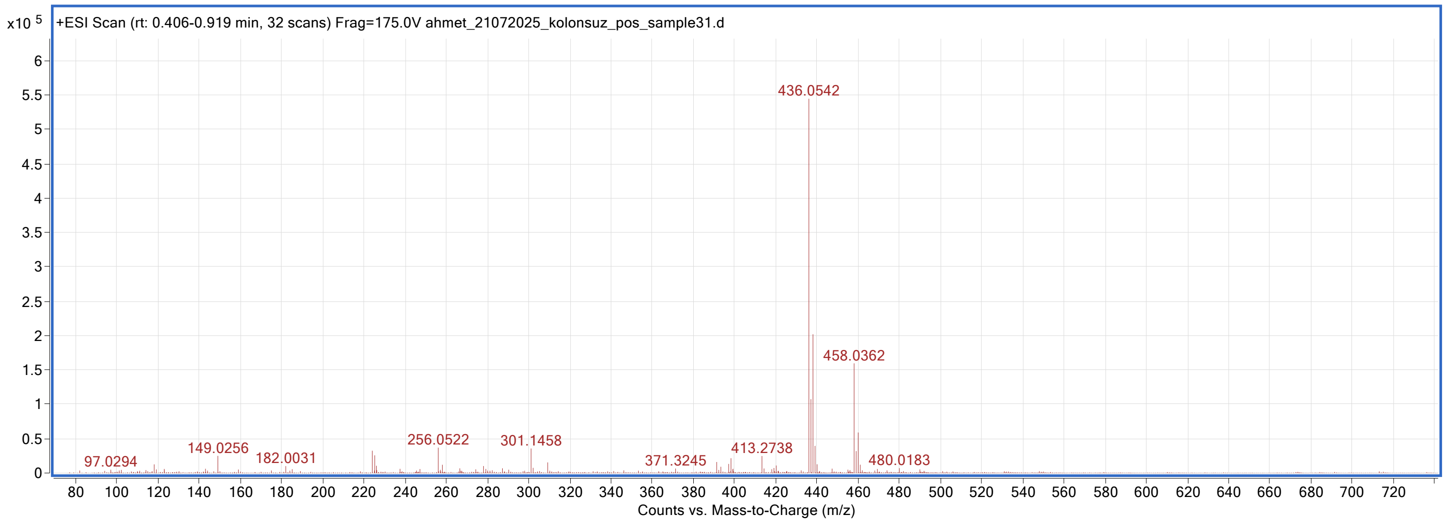

Figure S53. HRMS spectrum of compound 4ai'

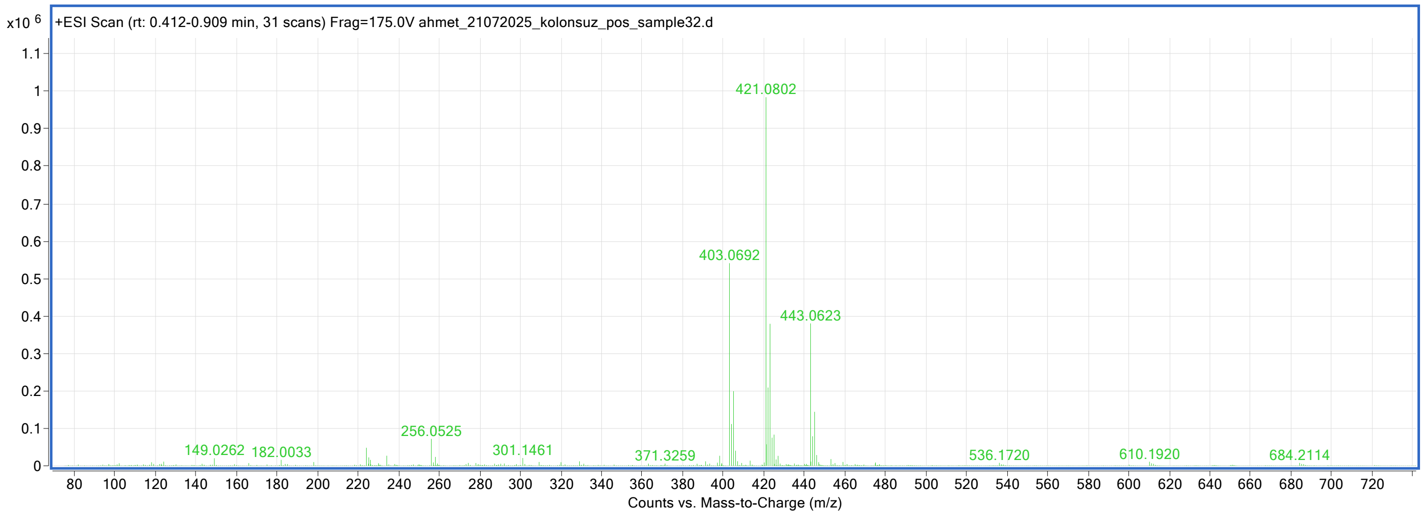

Figure S54. HRMS spectrum of compound 4aj'

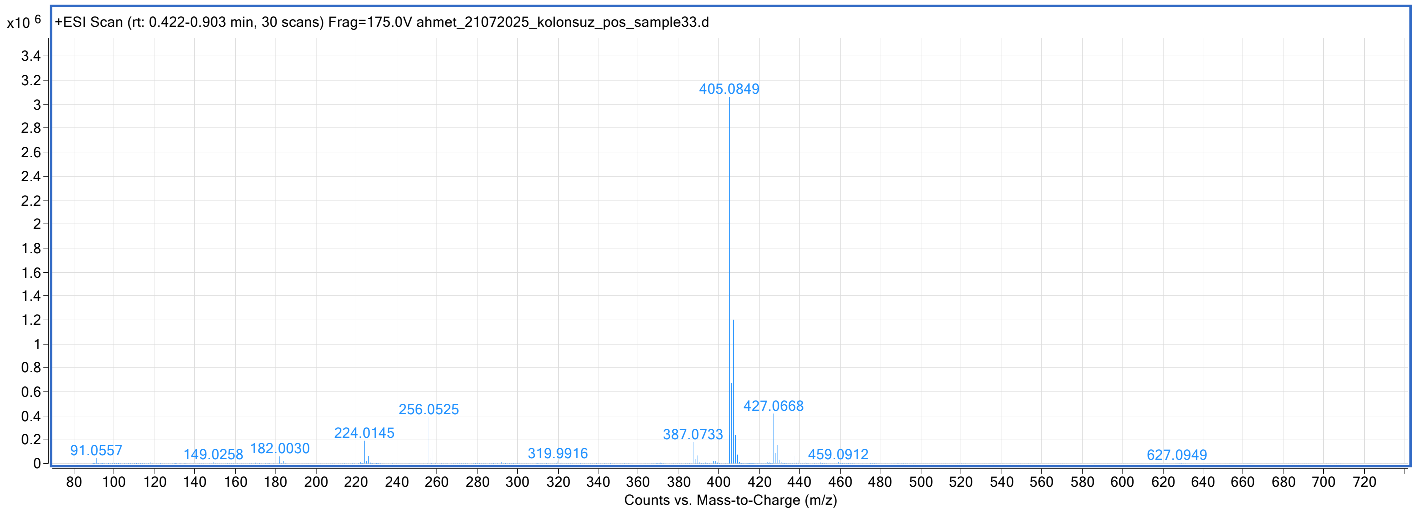

Figure S55. HRMS spectrum of compound 4ak'

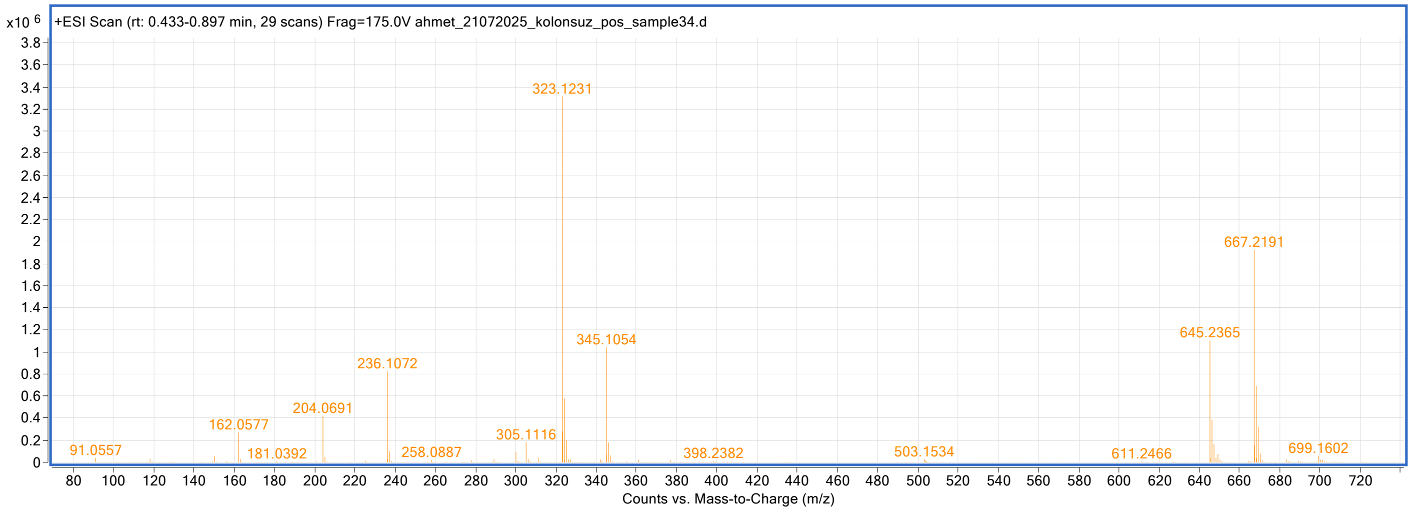

Figure S56. HRMS spectrum of compound 4ba'

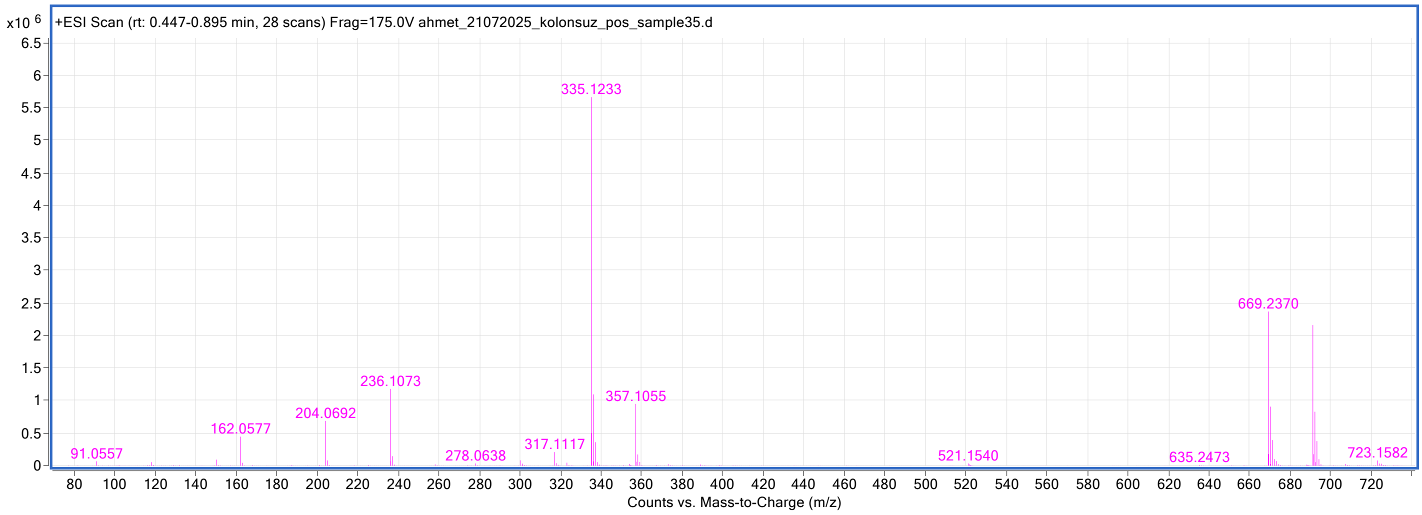

Figure S57. HRMS spectrum of compound 4bb'

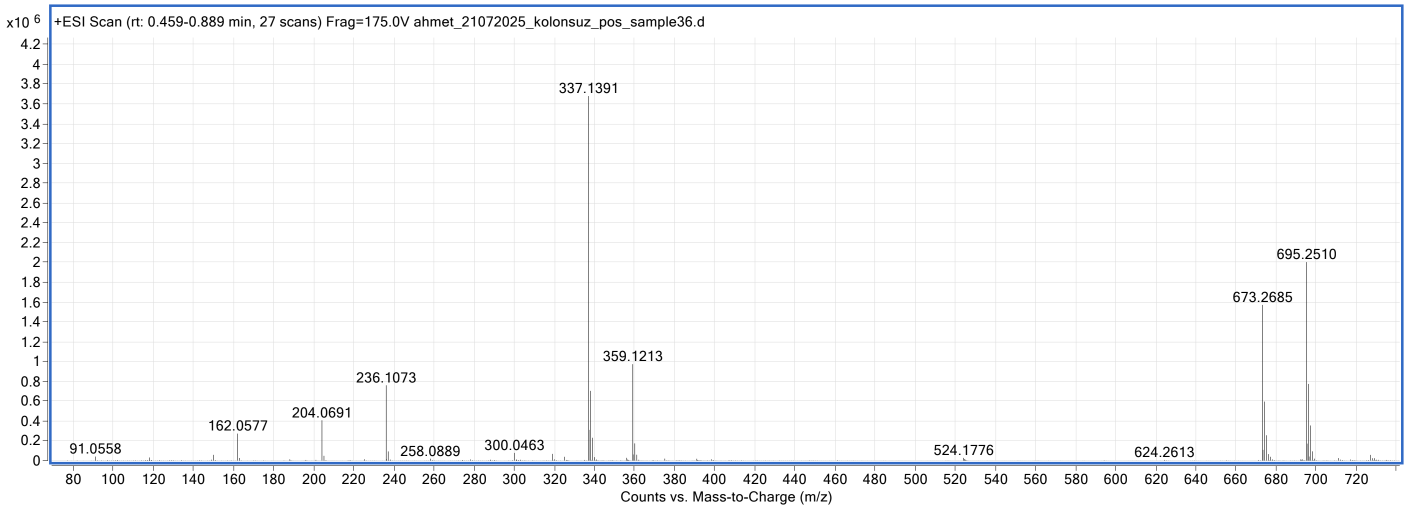

Figure S58. HRMS spectrum of compound 4bc'

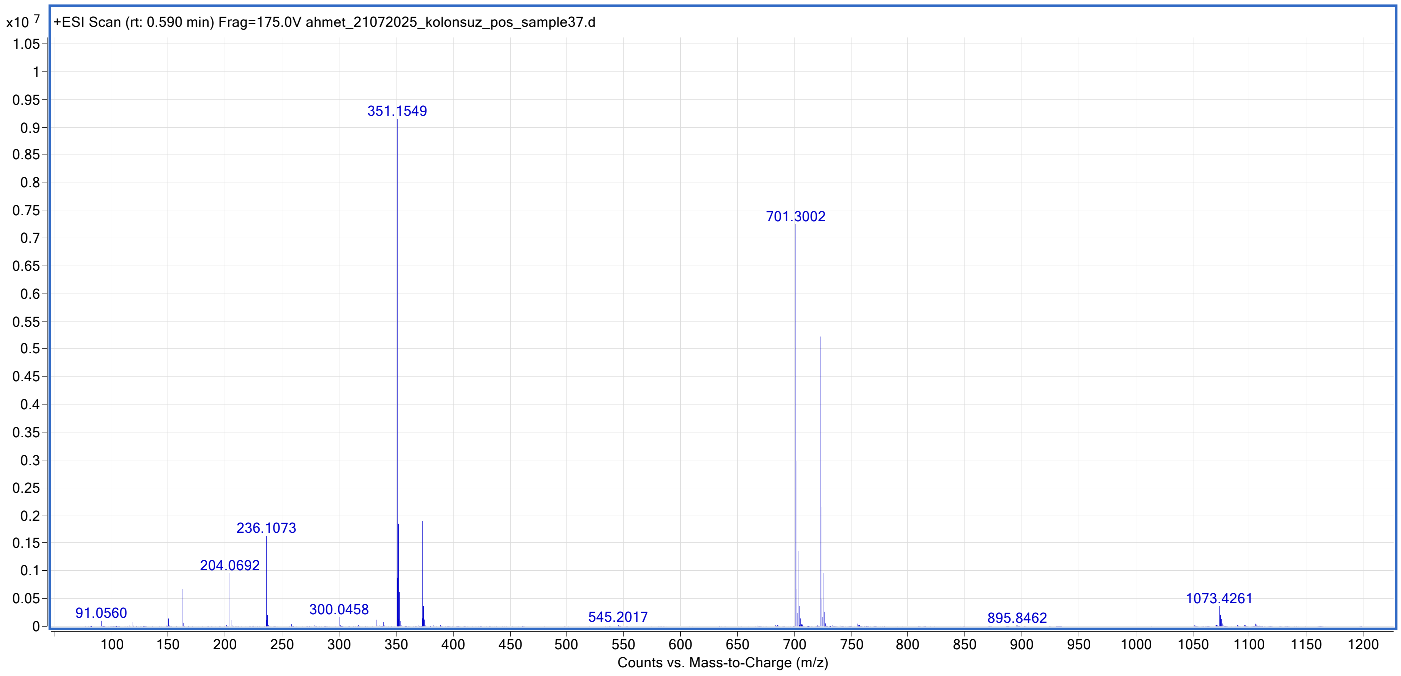

Figure S59. HRMS spectrum of compound 4bd'

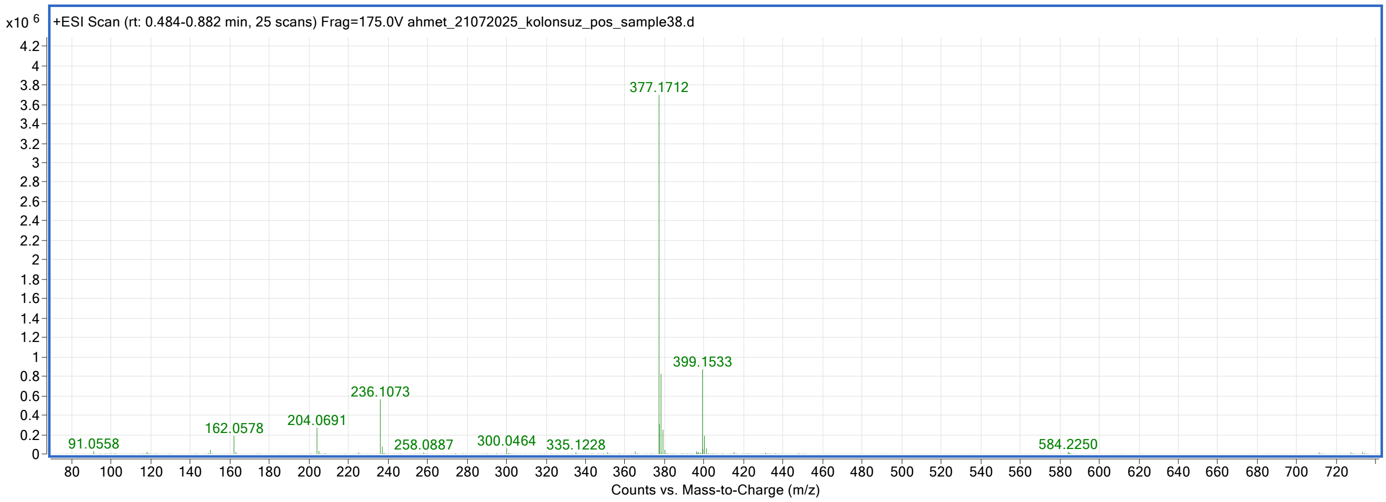

Figure S60. HRMS spectrum of compound 4be'

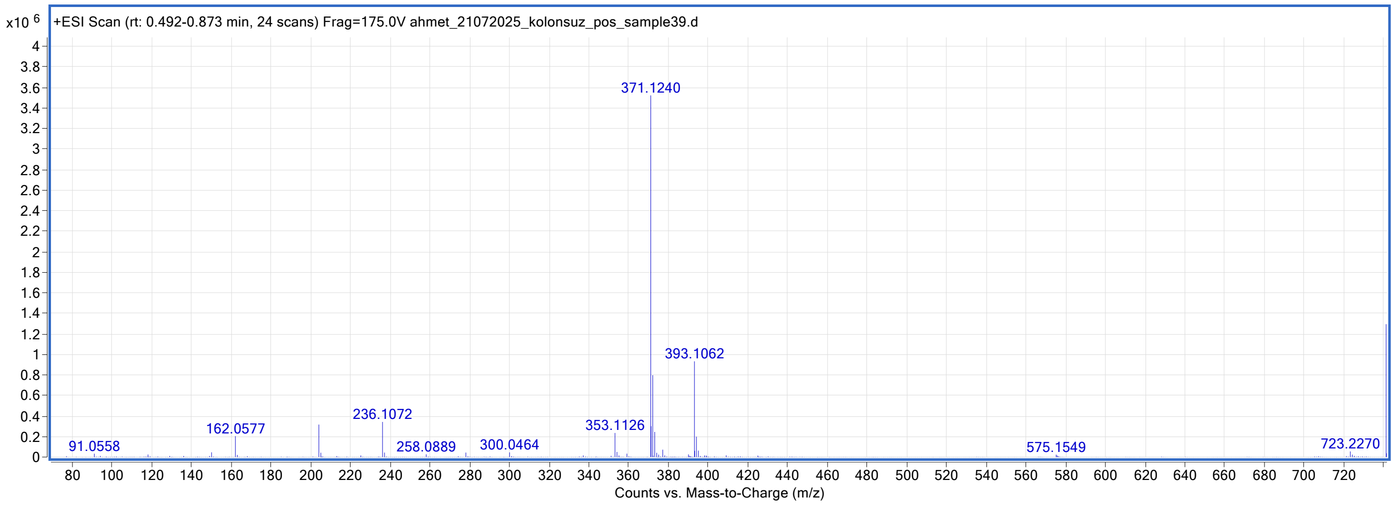

Figure S61. HRMS spectrum of compound 4bf'

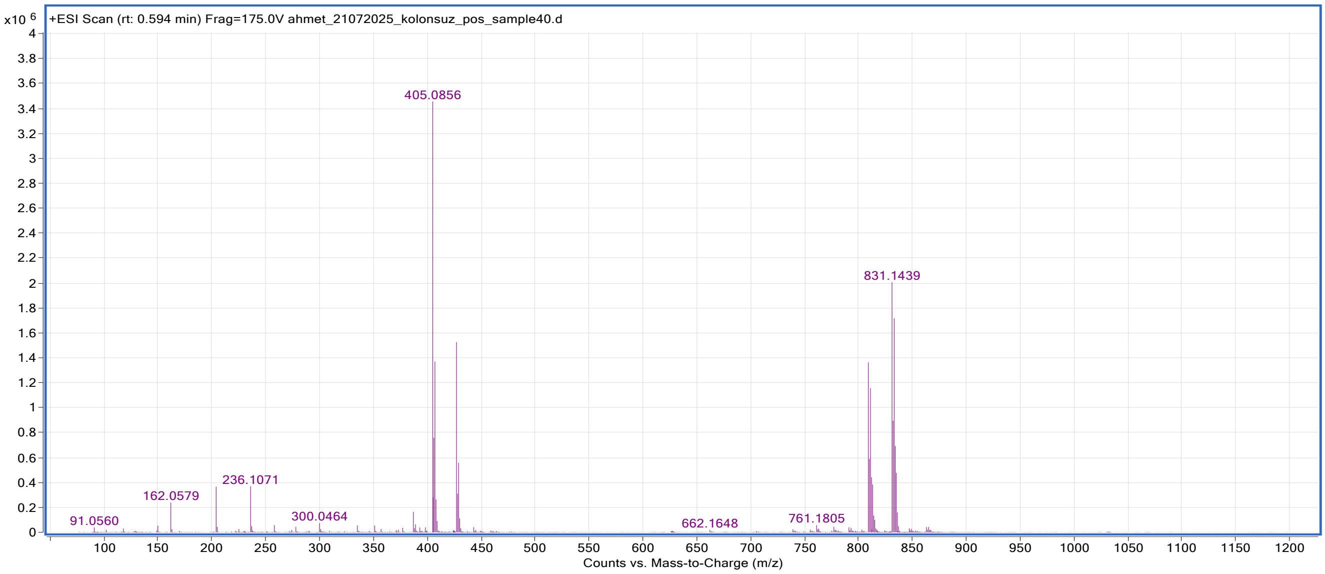

Figure S62. HRMS spectrum of compound 4bg'

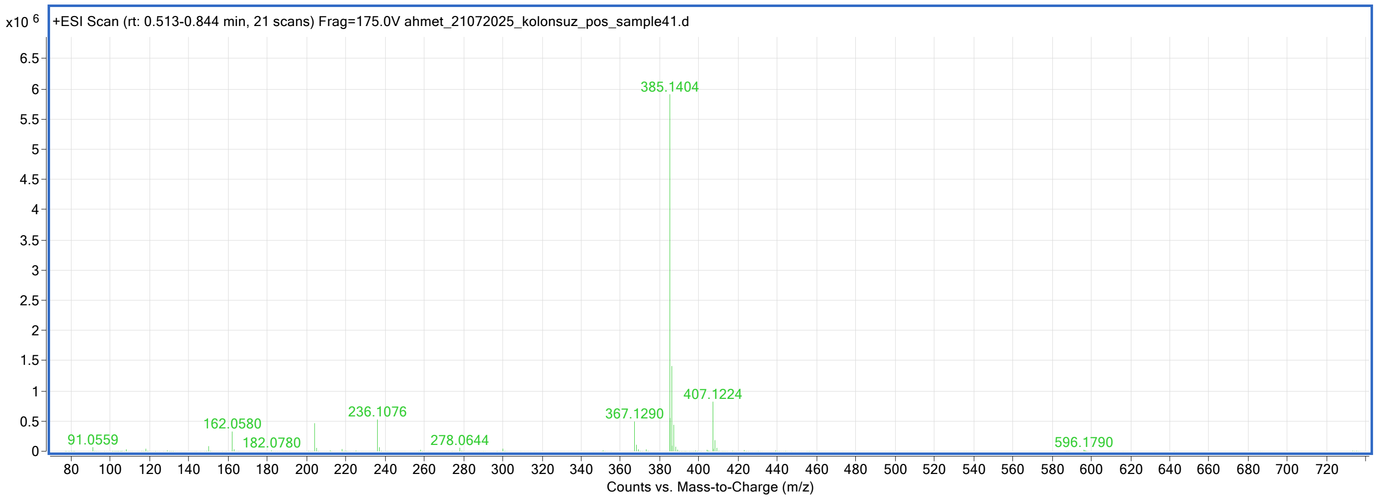

Figure S63. HRMS spectrum of compound 4bh'

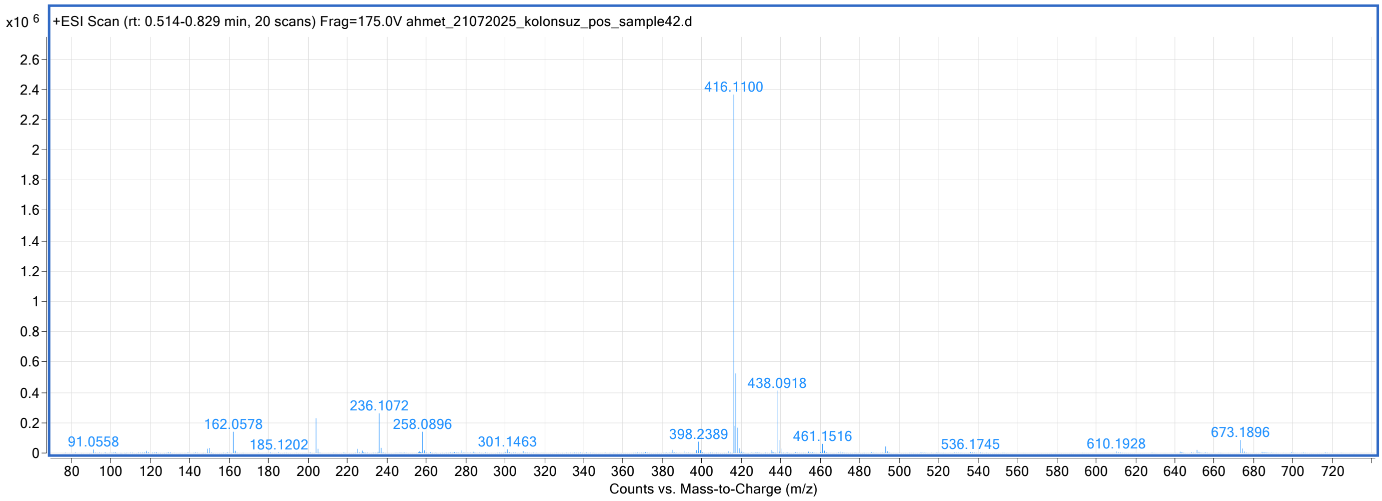

Figure S64. HRMS spectrum of compound 4bi'

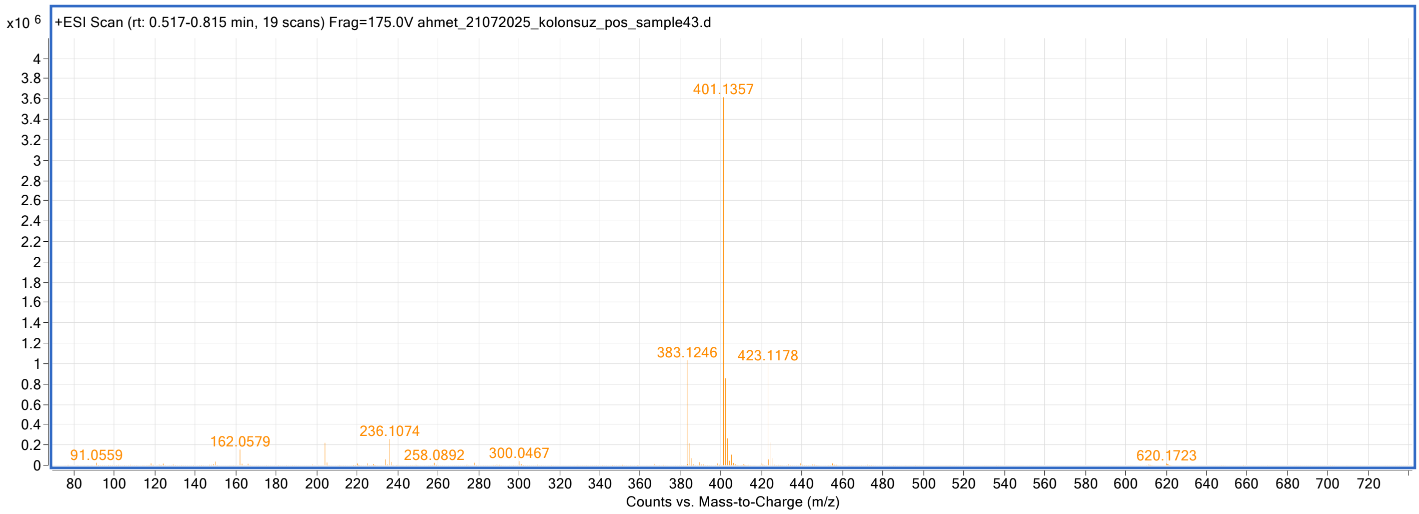

Figure S65. HRMS spectrum of compound 4bj'

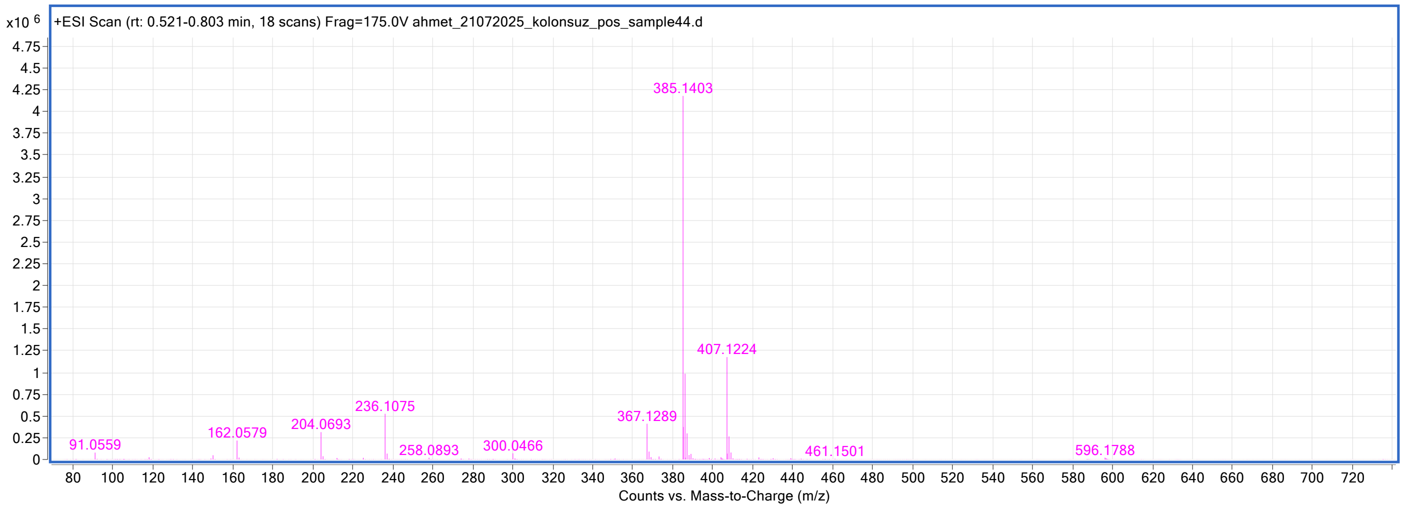

Figure S66. HRMS spectrum of compound 4bk'

Molecular Dynamic Studies

Additional images and tables were compiled.

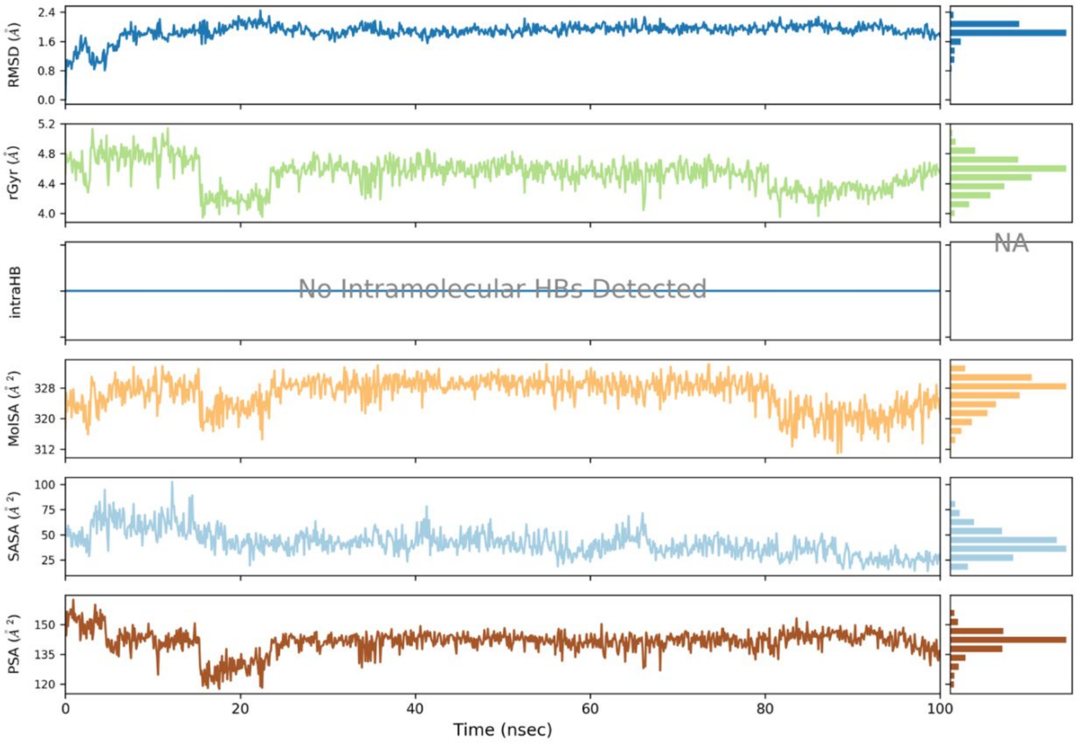

Figure S67. Molecular dynamic results of compound **4ac'–**AChE (PDB ID: 4EY7) complex.

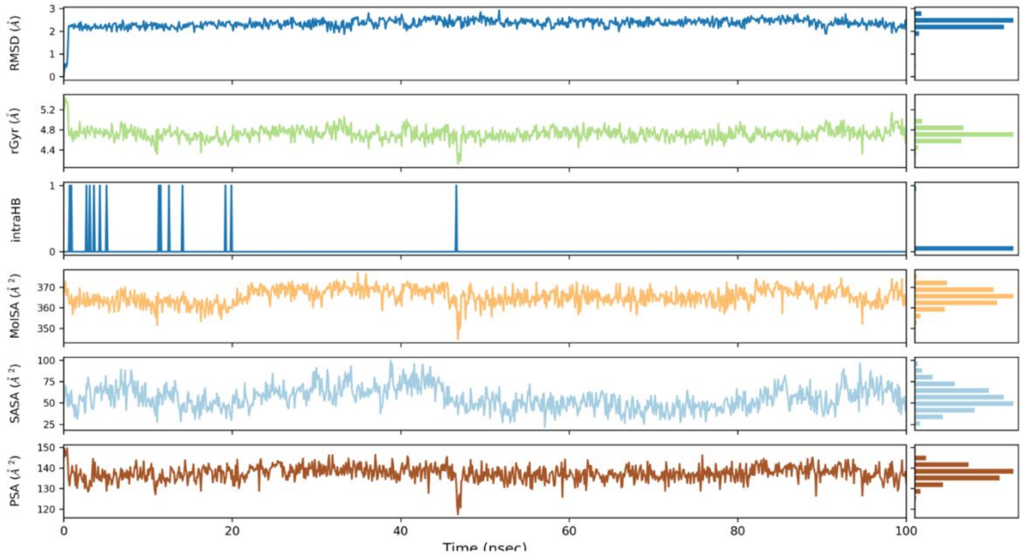

Figure S68. Molecular dynamic results of compound **4bk'–**AChE (PDB ID: 4EY7) complex.

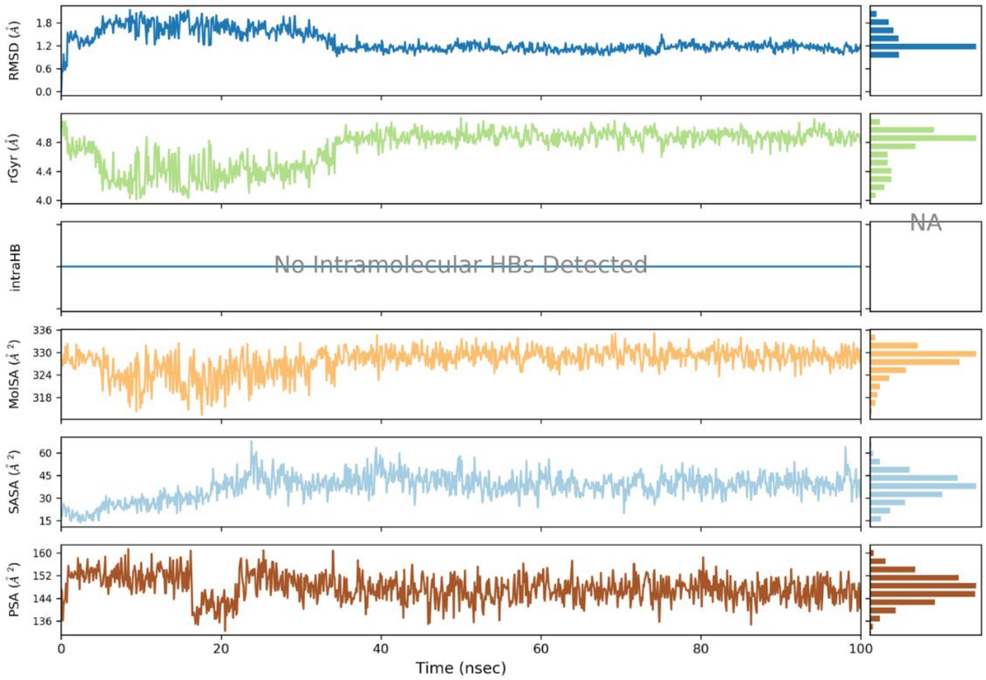

Figure S69. Molecular dynamic results of compound **4ac'**–MAO-B (PDB ID: 2V5Z) complex.

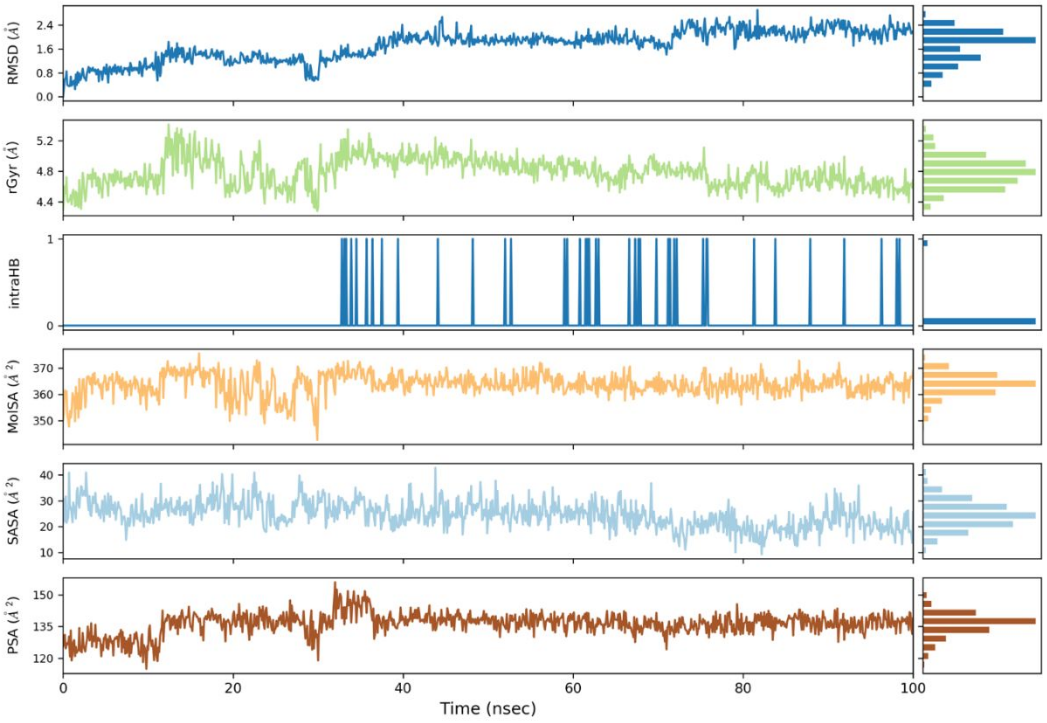

Figure S70. Molecular dynamic results of compound **4bk'**–MAO-B (PDB ID: 2V5Z) complex.

Table S1. Interacting amino acids and fractions for compounds **4ac'** and **4bk'** on AChE (PDB ID: 4EY7) enzyme.

<table>
<tr><th>Complex</th><th>Amino Acids Interacting Above 10%</th><th>Interaction Fractions</th></tr>
<tr><td rowspan="4">4ac'-4EY7</td><td rowspan="4">Tyr124 (H- bond: 34%)
Trp286 (π-π interaction: 23%)
Ser293 (Water mediated H-bond: 27%)
Phe295 (H- bond: 88%)
Arg296 (Water mediated H-bond: 42%)
Phe297 (Water mediated H-bond: 44%)
Tyr337 (Water mediated H-bond: 54%)</td><td>Water mediated H-bond (blue)
Tyr 72, Val73, Asp74, Thr75, Leu76, Gly82, Thr83, Trp86, Asn87, Gly121, Tyr124, Ser125, Gly126, Tyr133, Glu202, Trp286, Gln291, Glu292, Ser293, Val294, Phe295, Arg296, Phe297, Ser298, Tyr337, Tyr341, Tyr449</td></tr>
<tr><td>H-bond (green)
Thr83, Tyr124, Phe295, Tyr337, Tyr341</td></tr>
<tr><td>Ionic interaction (pink)
Ser125</td></tr>
<tr><td>Hydrophobic interaction (purple)
Trp86, Tyr124, Trp286, Leu289, Phe297, Tyr337, Phe338, Tyr341, Tyr449</td></tr>
<tr><td rowspan="4">4bk'-4EY7</td><td rowspan="4">Tyr72 (Water mediated H-bond: 10%)
Asp747 (H-bond: 20% and 77%)
Trp86 (π-π interaction: 24%)
Tyr124 (H- bond: 84%)
Trp286 (π-π interaction: 10%)
Phe295 (H- bond: 89%)
Arg296 (H-bond: 28%)
Tyr337 (H-bond: 22% and 29%)
Phe338 (π-π interaction: 19%)
Tyr341 (H-bond: 13%)</td><td>Water mediated H-bond (blue)
Tyr 72, Val73, Asp74, Leu76, Gly82, Thr83, Trp86, Asn87, Tyr124, Ser125, Leu289, Glu292, Ser293, Phe295, Arg296, Tyr337, Phe338, Tyr341, His447, Tyr449</td></tr>
<tr><td>H-bond (green)
Tyr72, Asp74, Tyr124, Ser125, Phe295, Arg296, Tyr337, Tyr341, Tyr449</td></tr>
<tr><td>Ionic interaction (pink)
Asp74, Trp86, Tyr124</td></tr>
<tr><td>Hydrophobic interaction (purple)
Tyr72, Trp86, Tyr124, Trp286, Phe297, Tyr337, Phe338, Tyr341, His447, Tyr449</td></tr>
</table>

Table S2. Interacting amino acids and fractions for compounds **4ac'** and **4bk'** on MAO-B (PDB ID: 2V5Z) enzyme.

Complex	Amino Acids Interacting Above 10%	Interaction Fractions
**4ac'-2V5Z**	Ile198 (H- bond: 71%)<br>Tyr326 (Water mediated H-bond: 21%)<br>Tyr398 (π-π interaction: 43%)<br>Tyr435 (π-π interaction: 45%)	***Water mediated H-bond (blue)***<br>Glu84, Pro102, Leu171, Ile198, Ile199, Ser200, Gln206, Thr314, Tyr326, Phe343, Tyr398, Tyr435
		H-bond (green)<br>Ile171, Ile198, Ile199, Ser200, Gln206, Tyr326, Tyr435
		Hydrophobic interaction (purple)<br>Leu88, Phe99, Phe103, Pro104, Leu171, Cys172, Tyr188, Ile198, Ile199, Ile316, Tyr326, Tyr398, Tyr435
**4bk'-2V5Z**	Phe168 (π-π interaction: 29%)<br>Cys172 (H-bond: 17%)<br>Tyr188 (H-bond: 13% and 32%)<br>Ile198 (H-bond: 62%)<br>Thr201 (Water mediated H-bond: 11%)<br>Gln206 (H-bond: 11% and 26%)<br>Tyr326 (Water mediated H-bond: 10%)<br>Tyr435 (π-π interaction: 12%)<br>Tyr435 (H-bond: 11%)	***Water mediated H-bond (blue)***<br>Phe99, Gly101, Phe103, Cys172, Tyr188, Ile199, Ser200, Thr201, Thr202, Gln206, Tyr326, Thr399, Gly434
		H-bond (green)<br>Cys172, Tyr188, Ile198, Ile199, Ser200, Gln206, Tyr326, Tyr435
		Hydrophobic interaction (purple)<br>Tyr60, Pro104, Trp119, Leu164, Leu167, Phe168, Leu171, Cys172, Tyr188, Ile198, Ile199, Ser200, Thr202, Gln206, Ile316, Tyr326, Leu328, Phe343, Tyr398, Tyr435
